# Enantioselective synthesis of β-aryl-γ-lactam derivatives via Heck–Matsuda desymmetrization of *N*-protected 2,5-dihydro-1*H*-pyrroles

**DOI:** 10.3762/bjoc.20.84

**Published:** 2024-04-29

**Authors:** Arnaldo G de Oliveira, Martí F Wang, Rafaela C Carmona, Danilo M Lustosa, Sergei A Gorbatov, Carlos R D Correia

**Affiliations:** 1 Department of Organic Chemistry, Chemistry Institute, University of Campinas, Rua Josué de Castro, 13083-970 Campinas, São Paulo, Brazil

**Keywords:** desymmetrization, enantioselective Heck–Matsuda reaction, lactam synthesis, *N,N*-ligands, palladium

## Abstract

We report herein an enantioselective palladium-catalyzed Heck–Matsuda reaction for the desymmetrization of *N*-protected 2,5-dihydro-1*H*-pyrroles with aryldiazonium salts, using the chiral *N*,*N*-ligand (*S*)-PyraBox. This strategy has allowed straightforward access to a diversity of 4-aryl-γ-lactams via Heck arylation followed by a sequential Jones oxidation. The overall method displays a broad scope and good enantioselectivity, favoring the (*R*) enantiomer. The applicability of the protocol is highlighted by the efficient enantioselective syntheses of the selective phosphodiesterase-4-inhibitor rolipram and the commercial drug baclofen as hydrochloride.

## Introduction

**Scheme 1:**
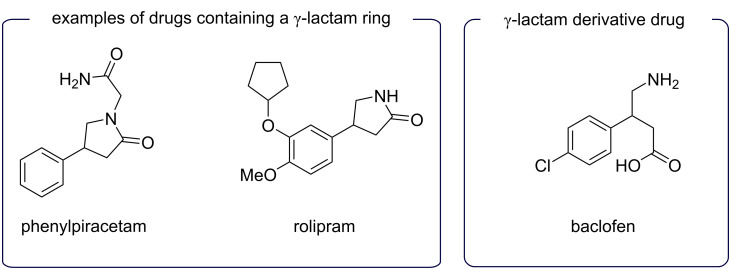
Examples of drugs containing a γ-lactam and derivative.

Desymmetrization reactions consist in the modification of a molecule with the loss of one or more symmetry elements, such as those which preclude chirality as in the transformation of a prochiral molecular entity into a chiral one [1]. It is a powerful and elegant strategy in asymmetric synthesis [2], which combined with the use of chiral ligands and transition-metal catalysts enabled many valuable transformations to increase molecular complexity in a synthetic route. The palladium-catalyzed coupling of arenediazonium salts with olefins, the Heck–Matsuda reaction, has been instrumental in this strategy involving the desymmetrization of cyclic systems [3], especially five-membered substrates [4–7]. As we have demonstrated previously, key five-membered olefins bearing heteroatoms can provide direct access to chiral sulfones, sulfoxides, phosphine oxides [8], phthalides, isochromanones, and lactones [9] in a very efficient and convenient manner. Despite our previous results in this area, the desymmetrization of 2,5-dihydro-1*H*-pyrroles posed some challenges due to substrate instability and undesirable side reactions. In 2003, we reported the Heck–Matsuda arylation of *N*-protected 2,5-dihydro-1*H*-pyrroles [10] to obtain 4-aryl-γ-lactams in a racemic manner [11], thus demonstrating the feasibility of this transformation. The γ-lactam ring is a privileged scaffold widely present in drugs and natural products [12–14], as shown in Scheme 1.

**Scheme 2:**
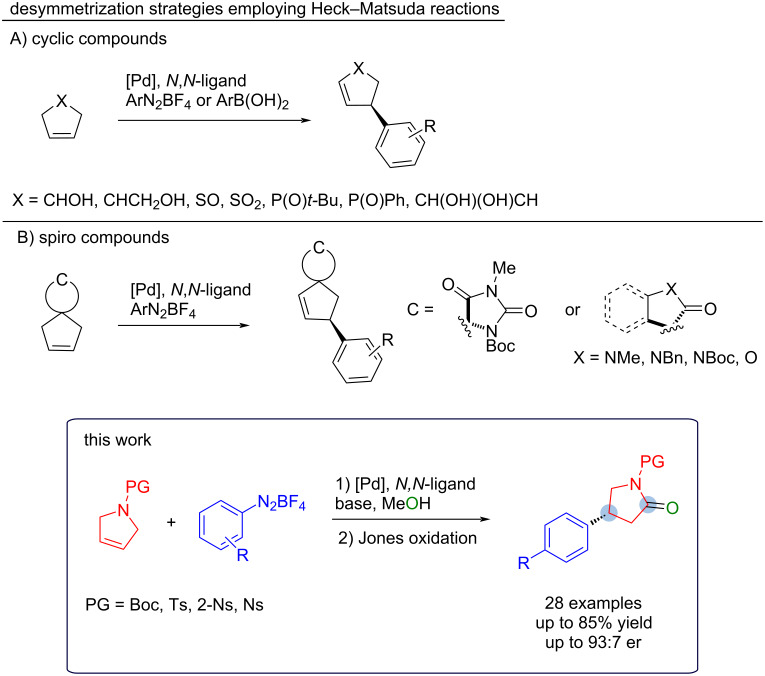
Desymmetrization strategies employing Heck-Matsuda reactions.

Herein, we report the effective desymmetrization strategy of *N*-protected 2,5-dihydro-1*H*-pyrroles using aryldiazonium salts and the chiral *N*,*N*-ligand (*S*)-PyraBox (Scheme 2). The obtained Heck adducts (methyl *N*,*O*-acetals) were efficiently converted into several arylated γ-lactams by a simple oxidation procedure (Jones oxidation). To demonstrate the applicability of the strategy, two of the chiral aryl-lactams were further derivatized to provide the selective phosphodiesterase-4 inhibitor (*R*)-rolipram (**5b**) [15], and the commercial drug (*R*)-baclofen hydrochloride (**6**), used to treat muscle spasticity from spinal cord injury and multiple sclerosis [16].

## Results and Discussion

### Desymmetrization of *N*-protected 2,5-dihydro-1*H*-pyrroles

#### Some initial results and reaction optimization

**Scheme 3:**
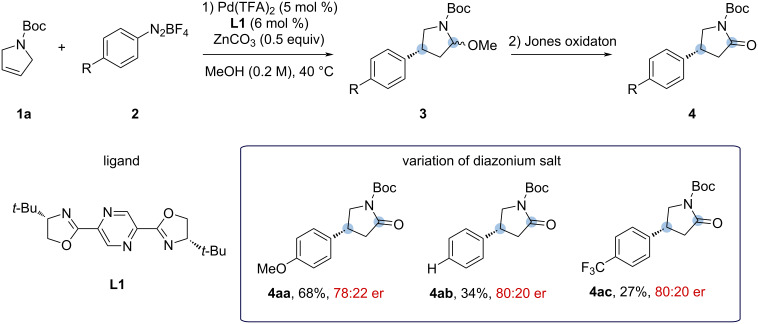
Heck–Matsuda reaction (1) and Jones oxidation (2) of the *N*-Boc-protected 2,5-dihydro-1*H*-pyrrole **1a**. Reaction conditions: 1) pyrroline **1a** (0.30 mmol, 1.0 equiv), aryldiazonium salt **2** (0.60 mmol, 2.0 equiv), Pd(TFA)_2_ (5 mol %), **L1** (*S*)-PyraBox (6 mol %), ZnCO_3_ (0.15 mmol, 0.5 equiv), and MeOH (1.5 mL, 0.2 M) at 40 °C, 4 h; 2) 1.0 mL of the Jones solution 2.5 M, 6 mL of acetone/water 3:1 (v/v), 1.5 h. Isolated yields were calculated from an average of two runs. Enantiomeric ratios (er) were determined by high-performance liquid chromatography (HPLC) analysis of the purified compounds.

Based on our previous results regarding the desymmetrization of hidantoins [17], we started this study with the *N*-Boc-protected dihydropyrrole **1a** using different electronic-demanding aryldiazonium salts and the standard reaction conditions for similar Heck–Matsuda reactions (Scheme 3), i.e., Pd(TFA)_2_ as the palladium source in combination with the pyrazinebisoxazoline ligand, (*S*)-PyraBox (**L1**), zinc carbonate as base, and methanol as solvent at 40 °C.

These initial conditions furnished 2-methoxypyrrolidines arylated at the 4-position, compound **3**, as Heck products as illustrated in Scheme 3. The presence of a methoxy group after the Heck–Matsuda reaction indicates methanolysis after arylation. Given the importance of the lactam rings, we envisioned a sequential Jones oxidation protocol without isolation of the methyl *N*,*O*-acetal products to obtain the corresponding lactams **4**. As observed in previous works [18], the oxidation step is practical and high-yielding, and the overall yield can be reported based on the isolated lactams.

By evaluating the electronics of the diazonium salt, we observed that the electron-donating *p*-OMe substituent performed better (**4aa**, 68% yield) when compared to neutral (**4ab**, 34% yield) and electron-withdrawing (**4ac**, 27% yield) ones, but no significant changes in the enantiomeric ratio were observed (Scheme 3).

**Figure 1:**
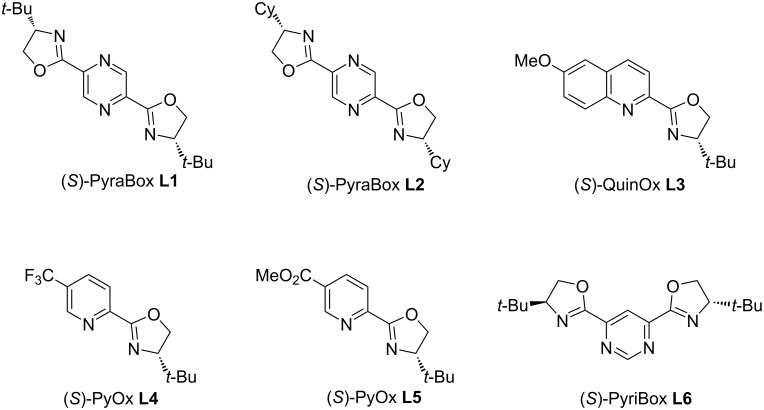
*N*,*N*-Ligands evaluated in this work.

Despite the formation of the hemiaminal ether as the major product, the formation of a minor *N*-Boc pyrrole was also observed as a side-product. To circumvent this side reaction, we envisioned that a more electron-withdrawing protecting group could reduce the tendency of the starting olefin to oxidation. Therefore, the *N*-tosylated 2,5-dihydro-1*H*-pyrrole **1b** was evaluated under the same reaction conditions with the same three aryldiazonium salts used before. Before exploring the reactivity of the olefin towards other aryldiazonium salts, we performed a brief optimization of the process by testing several other *N*,*N*-ligands. Therefore, five other *N*,*N*-ligands were evaluated as follows: PyraBox (**L2**), QuinOx (**L3**), PyOx **L4** and **L5**, and PyriBOx (**L6**) (Figure 1).

However, neither one of these new ligands performed better than **L1** (see Table 1 below). In an attempt to enhance the protocol performance, we also evaluated the palladium source as indicated in Table 1. Switching Pd(TFA)_2_ by Pd(OAc)_2_ led to a minor increase in the yield, but without any changes in the er. Pd(acac)_2_ and Pd(MeCN)_2_(OTs)_2_ were also tested without significant improvements.

**Table 1:** Optimization of the reaction conditions with tosyl-protected pyrroline **1b**.^a^

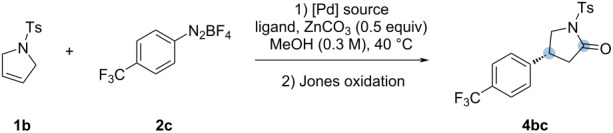				

Entry	Ligand	[Pd] source	**4bc** %^b^	er^c^

**1**	**L1** ^d^	**Pd(TFA)** **_2_**	**62**	**85:15**
2	L2^d^	Pd(TFA)_2_	56	57:43
3	L3^e^	Pd(TFA)_2_	49	56:44
4	L4^e^	Pd(TFA)_2_	57	77:23
5	L5^e^	Pd(TFA)_2_	54	72:28
6	L6^d^	Pd(TFA)_2_	51	69:31
7	L1^d^	Pd(OAc)_2_	65	85:15
8	L1^d^	Pd_2_dba_3_	64	84:16
9	L1^d^	Pd(acac)_2_	68	84:16
10	L1^d^	Pd(MeCN)_2_(OTs)_2_	62	83:17

^a^Reaction conditions: pyrroline **1b** (0.30 mmol, 1.0 equiv), 4-(trifluoromethyl)benzenediazonium tetrafluoroborate (**2c**, 0.60 mmol, 2.0 equiv), Pd(TFA)_2_ (5 mol %), ligand, ZnCO_3_ (0.15 mmol, 0.5 equiv), MeOH (1.0 mL, 0.3 M), 40 °C, 4 h. Jones conditions: 1.0 mL Jones solution 2.5 M, 6 mL of acetone/water 3:1 (v/v), 1.5 h. ^b^NMR yields. ^c^Determined by HPLC analysis. ^d^Ligand: 6 mol %. ^e^Ligand: 11 mol %.

**Scheme 4:**
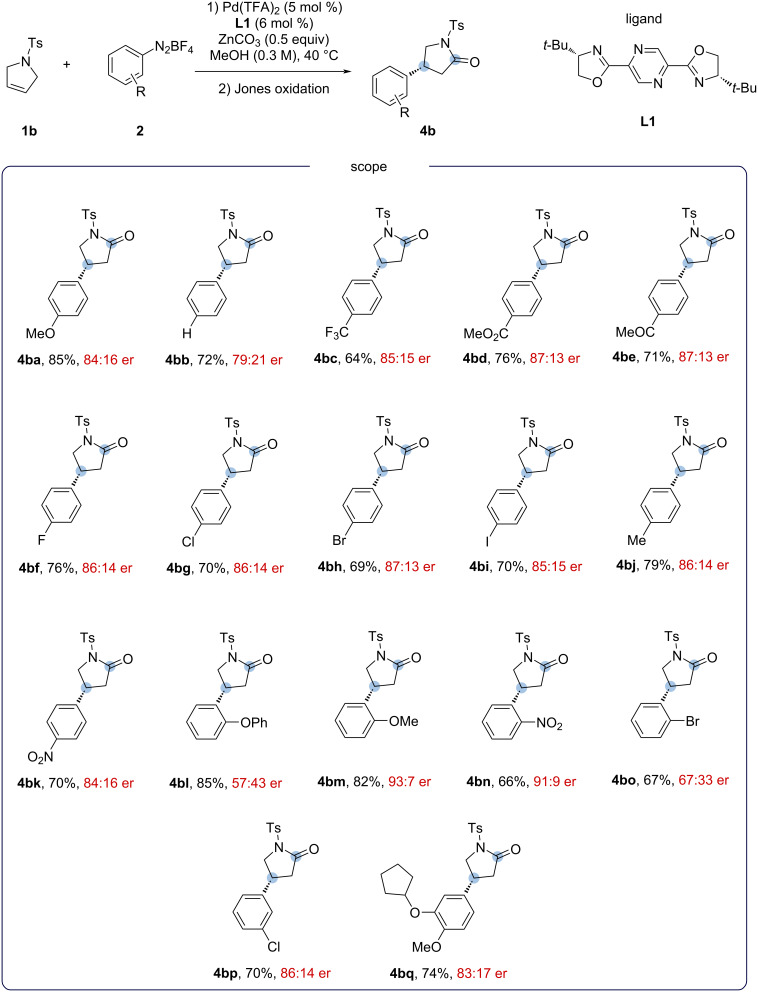
Heck–Matsuda reaction of *N*-tosyl-2,5-dihydro-1*H*-pyrrole (**1b**). Reaction conditions: 1) pyrroline **1b** (0.30 mmol, 1.0 equiv), aryldiazonium salts **2** (0.60 mmol, 2.0 equiv), Pd(TFA)_2_ (5 mol %), **L1** (6 mol %), ZnCO_3_ (0.15 mmol, 0.5 equiv), MeOH (1.0 mL, 0.3 M), 40 °C, 4 h. 2) 1.0 mL Jones solution 2.5 M, 6 mL of acetone/water 3:1 (v/v), 1.5 h. Isolated yields were calculated from an average of two runs. Enantiomeric ratio (er) determined by high-performance liquid chromatography (HPLC) analysis of the purified compounds.

Despite the fact that palladium acetate slightly better performed as shown in Table 1, we decided to continue with palladium trifluoroacetate due to its higher reactivity in forming palladium complexes with *N*,*N*-ligands. Therefore, we decided to maintain our initial conditions using Pd(TFA)_2_ and proceeded to the evaluation of the scope of the Heck–Matsuda arylation as shown in Scheme 4. Gratifyingly, the new reaction conditions with the tosylpyrroline **1b** showed significant improvements in yield and enantioselectivities (**4ba** and **4bc** in Scheme 4). Somewhat surprisingly, no enhancement in the enantiomeric ratio was observed for the lactam **4bb**.

With the optimized conditions in hand, we evaluated the scope of the method by varying the aryldiazonium salts. For aryldiazonium salts bearing *p*-substituted groups, there is very little influence in the enantiomeric ratios, although electron-donating groups performed slightly better in terms of yield, as observed before for the *N*-Boc-protected pyrrolines. Weak electron-donating groups such as the methyl group furnished compound **4bj** in a higher yield and good er. Carbonyl-containing electron-withdrawing groups such as methyl ester and ketone did not show much of an effect in the outcome of the reaction, providing compounds **4bd** and **4be** in high yields and good er. On the other hand, product **4bq** was obtained in a higher yield but with a lower er. The *p*-halogen-containing derivatives **4bf**, **4bg**, **4bh**, and **4bi** were all obtained in high yields and good er*.* We also evaluated the change of some substituents to the *ortho* position. This change furnished compound **4bm** in higher yield and excellent er. However, when the bulkiness of the substituent was increased, as in compounds **4bl** and **4bo** (*o*-phenoxy and *o*-bromo group, respectively), the er dropped considerably. Finally, a strong electron-withdrawing group in the *ortho* position such as nitro (**4bn**) was met with a decrease in yield (66%), but with a higher er.

During the development of the scope, the hemiaminal ethers (Heck–Matsuda products) were found to be somehow unstable when concentrated to dryness during work-up. We hypothesize that a possible cause of such instability might consist in the formation of a highly electrophilic iminium ion upon protonation of the hemiaminal ether by silica or glassware acidity and further elimination of methanol favored by the evaporation process. The instability of hemiaminal ethers was previously described in literature [19] during work-up. We then found that careful control of the drying conditions, thus avoiding complete drying of the crude mixture prevents degradation of the Heck products. We then established a robust protocol consisting of successive additions of acetone to the crude mixture, followed by careful rotaevaporation. This procedure gradually removes most of the methanol, allowing the sequential Jones oxidation step to take place without any significant losses (see Supporting Information File 1 for details).

**Scheme 5:**
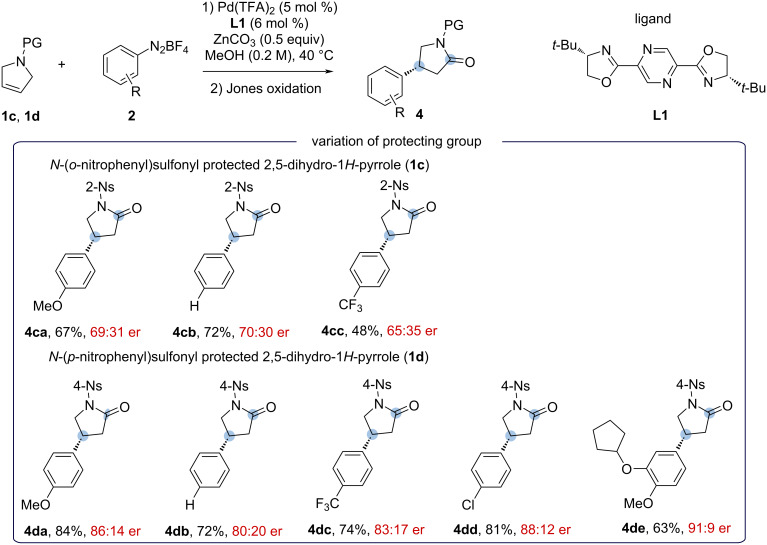
Heck–Matsuda reaction of the protected 2,5-dihydro-1*H*-pyrrole with Ns and 2-Ns groups (pyrrolines **1c**, **1d**). Reaction conditions: 1) pyrroline **1c** or **1d** (0.30 mmol, 1.0 equiv), aryldiazonium salts **2** (0.60 mmol, 2.0 equiv), Pd(TFA)_2_ (5 mol %), **L1** (6 mol %), ZnCO_3_ (0.15 mmol, 0.5 equiv), MeOH (1.5 mL, 0.2 M), 40 °C, 4 h. 2) 1.0 mL Jones solution 2.5 M, 6 mL of acetone/water 3:1 (v/v), 1.5 h. Isolated yields were calculated from an average of two runs. Enantiomeric ratio (er) determined by high-performance liquid chromatography (HPLC) analysis of the purified compounds.

Given the presence of the 4-aryl-γ-lactam motif in the phosphodiesterase-4 inhibitor rolipram, and in a synthetic intermediate for the baclofen drug, the Heck products **4bg** and **4bq** were used as starting material for their syntheses. *N*-Tosylated lactams **4bg** and **4bq** were then submitted to deprotection protocols as described in the literature [20–21]. However, the removal of the tosyl group of pyrroline **1b** proved to be a challenging task. After several unsuccessful attempts to remove the tosyl group, we decided to evaluate the (*p*-nitrophenyl)sulfonyl (Ns) and (*o*-nitrophenyl)sulfonyl (2-Ns) as alternative protecting groups of 2,5-dihydro-1*H*-pyrrole (Scheme 5). Although the results with the 2-Ns protecting group were somewhat disappointing, the results with 4-Ns group were more promising, even with a welcome increase in the enantiomeric ratio in some cases (**4dd** and **4de**).

#### Synthesis of (*R*)-baclofen hydrochloride (**6**) from **4dd** and (*R*)-rolipram (**5b**) from **4de**

**Scheme 6:**
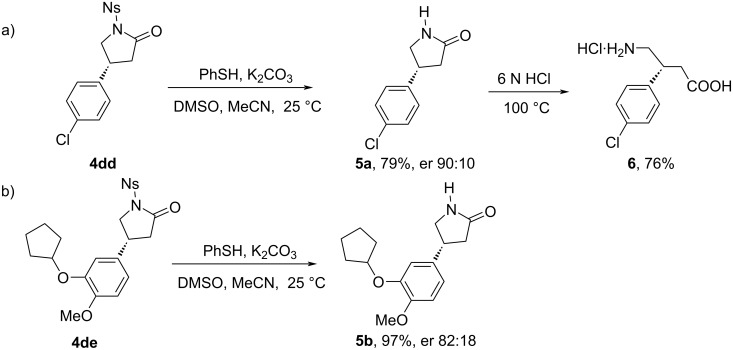
Synthesis of (*R*)-baclofen hydrochloride (**6**) from **4dd** and (*R*)-rolipram (**5b**) from **4de**. Reaction conditions: a) **4dd** (0.20 mmol, 1.0 equiv), PhSH (0.30 mmol, 1.5 equiv), K_2_CO_3_ (0.40 mmol, 2 equiv), MeCN (2 mL), DMSO (0.75 mL), 25 °C, 2 h, then 6 N HCl (0.5 mL), 100 °C, 10 h. Reaction conditions: b) **4de** (0.105 mmol, 1.0 equiv), PhSH (0.16 mmol, 1.5 equiv), K_2_CO_3_ (0.21 mmol, 2 equiv), MeCN (1 mL), DMSO (0.4 mL), 25 °C, 2 h. Enantiomeric ratio (er) determined by high-performance liquid chromatography (HPLC) analysis of the purified compounds.

To further demonstrate the applicability of this method, the aryl-lactams **4dd** and **4de** were successfully converted into the selective phosphodiesterase-4 inhibitor (*R*)-rolipram (**5b**) [15], and the commercial drug (*R*)-baclofen for the treatment of muscle spasticity from spinal cord injury and multiple sclerosis [16]. Among all the sulfonyl-protecting groups used in this work, the removal of the *N*-nosyl group required milder conditions [22]. Deprotection of *N*-nosylated **4dd** and **4de** with thiophenol and K_2_CO_3_ at room temperature gave the NH-unprotected γ-lactam **5a** and the drug (*R*)-rolipram **5b** in 79% and 97% yields, respectively, with excellent enantioselectivity. Hydrolysis of γ-lactam **5a** in 6 N HCl aqueous solution at 100 °C for 10 hours then led to the formation of (*R*)-baclofen hydrochloride (**6**) in 76% yield (Scheme 6). The total yields were determined to be 49% for (*R*)-baclofen hydrochloride (**6**) and 61% (*R*)-rolipram (**5b**) from starting pyrrolidine **1d**.

#### Determination of the absolute stereochemistry of the Heck adducts/lactams and rationalization of the enantioselectivity

**Scheme 7:**
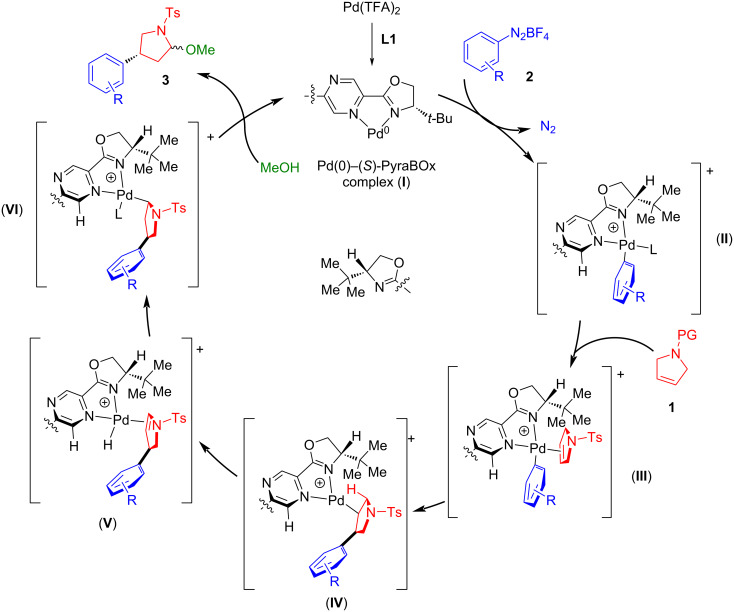
A rationale for the catalytic cycle for the Heck–Matsuda reaction of the protected 2,5-dihydro-1*H*-pyrroles with aryldiazonium salts catalyzed by a (*S*)-PyraBox–palladium complex.

**Figure 2:**
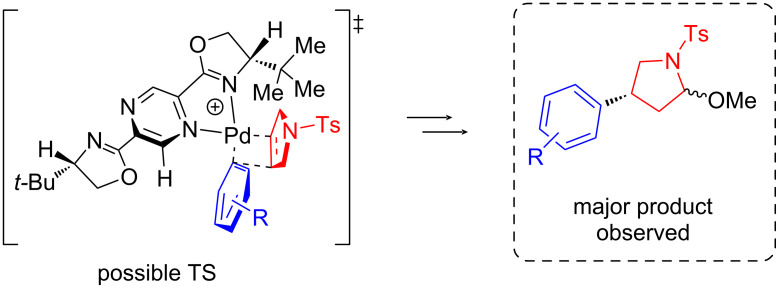
Rationalization of the enantioselectivity obtained in the Heck–Matsuda reaction of protected 2,5-dihydro-1*H*-pyrrole with aryldiazonium salts catalyzed by the (*S*)-PyraBox–palladium complex.

The absolute stereochemistry of the products was determined by the correlation of their optical rotations with that of the previously reported aryl-lactam **4bb** [23], and its deprotected analog [24], as well as with the intermediates **5b**, **5a**, and **6** in the (*R*)-rolipram (**5b**) and (*R*)-baclofen hydrochloride (**6**) [25] syntheses. Assignment of the stereochemistry of all other lactams as *R* was done by analogy. The assignment of the absolute stereochemistry allowed us to propose a rationale for the Heck–Matsuda reaction (Scheme 7). Upon activation of the catalyst (**I**), oxidative addition of aryldiazonium salt and subsequent nitrogen release generates the cationic palladium(II)–*N,N-*ligand complex (**II**), to which the pyrrolidine substrate coordinates (**III**). Next, migratory insertion takes place generating the alkylpalladium species (**IV**), which upon a sequence of β-elimination (**V**) and hydride insertion leads to alkylpalladium intermediate (**VI**). Finally, upon methanolysis, the hemiaminal ether product **3** is formed. We hypothesize that the enantioselectivity-determining step consists of the migratory insertion of the aryl group bonded to palladium to the pyrroline. The steric effect of the *t*-Bu group favors the coordination of the pyrroline with the protecting group upward, therefore creating an asymmetric center with absolute configuration (*R*), in accordance with experimental results. A rationalization for the transition state that would lead to the observed outcome is depicted in Figure 2.

## Conclusion

The palladium-catalyzed Heck–Matsuda desymmetrization of *N*-protected 2,5-dihydro-1*H*-pyrroles with aryldiazonium salts was successfully accomplished. The synthetic protocol employed the *N,N*-ligand (*S*)-PyraBox to provide several 4-substituted γ-lactams in an enantioselective fashion, with broad scope and good enantioselectivities, with yields up to 85% and er up to 93:7. The methodology was shown to be robust, allowing the use of different protecting groups at the nitrogen of the 4-pyrroline substrate. We also report straightforward synthetic routes to obtain (*R*)-rolipram (**5b**, 61% overall yield, 3 steps, 82:18 er) and (*R*)-baclofen hydrochloride (**6**, 49% overall yield, 4 steps, 90:10 er) using the Heck–Matsuda reaction as a key step for constructing the stereogenic center.

## Supporting Information

File 1 Experimental procedures and characterization data for the new compounds.

## Data Availability

All data that supports the findings of this study is available in the published article and/or the supporting information to this article.
