# Supplementary material for: Enantioselective synthesis of β-aryl-γ-lactam derivatives via Heck–Matsuda desymmetrization of N-protected 2,5-dihydro-1H-pyrroles
Source: Beilstein J Org Chem. 2024 Apr 29;20:940–9. doi: 10.3762/bjoc.20.84 (PMC11070958; doi:10.3762/bjoc.20.84)
Supplement: File 1 — Experimental procedures and characterization data for the new compounds. [file Beilstein_J_Org_Chem-20-940-s001.pdf]

BEILSTEIN JOURNAL OF ORGANIC CHEMISTRY

Supporting Information

for

Enantioselective synthesis of β-aryl-γ-lactam derivatives via Heck–Matsuda desymmetrization of *N*-protected 2,5-dihydro-1*H*-pyrroles

Arnaldo G. de Oliveira Jr., Martí F. Wang, Rafaela C. Carmona, Danilo M. Lustosa, Sergei A. Gorbatov and Carlos R. D. Correia

Beilstein J. Org. Chem. **2024,** *20,* 940–949. doi:10.3762/bjoc.20.84

Experimental procedures and characterization data for the new compounds

License and Terms: This is a supporting information file under the terms of the Creative Commons Attribution License (https://creativecommons.org/licenses/by/4.0). Please note that the reuse, redistribution and reproduction in particular requires that the author(s) and source are credited and that individual graphics may be subject to special legal provisions.
The license is subject to the *Beilstein Journal of Organic Chemistry* terms and conditions: (https://www.beilstein-journals.org/bjoc/terms)

1. General information

Heck–Matsuda reactions were carried out in a 4-mL screw-top vial under air with no precautions taken to exclude moisture. Reaction temperatures are reported as the temperature of the heat transfer medium surrounding the vessel. Yields refer to isolated compounds, unless stated otherwise. Solvents used for chromatography were technical grade and were distilled prior to use. Aryldiazonium tetrafluoroborate salts were prepared according to the literature. Ligands[1–4] and olefins[5–8] were prepared according to the literature. Commercially available chemicals were purchased and used as received, unless otherwise noted. Racemic samples were prepared following general procedures but without the use of any ligand.

Thin layer chromatography (TLC) was performed employing Merck® Silica gel 60 F254 fluorescent treated plates. Visualization was accomplished with UV light (254 nm), $KMnO_4$, *p*-anisaldehyde, ceric ammonium molybdate staining solution followed by heating, or Dragendorff reagent.

Flash column chromatography (FCC) purifications were performed by flash column chromatography using Merck® Silica gel 60 (230-400 mesh) as stationary phase and on a Biotage-Isolera One flash purification system employing Biotage® SNAP Ultra 10 g, 25 g or 50 g as stationary phase operating in gradient mode (EtOAc/hexanes).

Optical rotation data were measured on a Perkin Elmer 341 polarimeter with a sodium lamp using a 1.0 cm quartz glass cell and are reported as follows: $[\alpha]^{T}_{D}$ (°C) (*c* (g/100 mL), solvent).

NMR spectra were recorded at various field strengths, as indicated, using Bruker DPX250 (250 MHz for ^{1}H NMR and 62.5 MHz for ^{13}C NMR), Bruker Avance 400 (400 MHz for ^{1}H NMR and 100 MHz for ^{13}C NMR), Bruker Avance 500 (500 MHz for ^{1}H and 125 MHz for ^{13}C NMR), or Bruker Avance 600 (600 MHz for ^{1}H and 150 MHz for ^{13}C NMR) spectrometers. 1,3-Bis(trifluoromethyl)-5-bromobenzene was used as internal standard for the determination of chemical yields by ^{1}H NMR. Chemical shifts (δ) are reported in ppm using residual undeuterated solvent as an internal standard ($CHCl_3$ at 7.26 ppm for ^{1}H NMR spectra and $CDCl_3$ at 77.16 ppm for ^{13}C NMR spectra). Multiplicity data are reported as follows: s = singlet, d = doublet, t = triplet, q = quartet, quint = quintet, sext = sextet, br s = broad singlet, dd = doublet of doublets, dt = doublet of triplets, ddd = doublet of doublet of doublets, ddt = doublet of doublet of triplets, dtd = doublet of triplet of doublets, dqd = doublet of quartet of doublets, and m = multiplet. The multiplicity is followed by the coupling constant(s) in Hz and integration.

High-resolution mass spectra (HRMS) was recorded using electrospray ionization (ESI) on a Waters Xevo Q-Tof, Bruker qTOF Impact II and Q-Exactive Plus-Thermo Fisher Scientific.

Enantiomeric ratios (er) were calculated through integration of enantiomers corresponding signals, set by racemic samples. The products were analyzed through high-performance liquid chromatography (HPLC) on an

Agilent Technologies 1260 Infinity with a DAD detector equipped with Daicel Chiralpak® chiral columns as stationary phase and hexanes:iPrOH mixtures as mobile phase, or through supercritical fluid chromatography (SFC) performed on an Agilent Technologies 1260 SFC Infinity with an DAD detector equipped with Daicel Chiralpak® chiral columns as the stationary phase and CO_2:MeOH mixtures as the mobile phase.

2. Preparation and characterization of substrates and reagents

Ligands **L1**, **L3**, **L4**, **L5**, and **L6** were prepared according to previously reported procedures [1-4].

Olefins **1a**, **1b**, **1c**, and **1d** were prepared according to previously reported procedures [5-8]. They are also commercially available.

2.1 Preparation of ligand **L2**

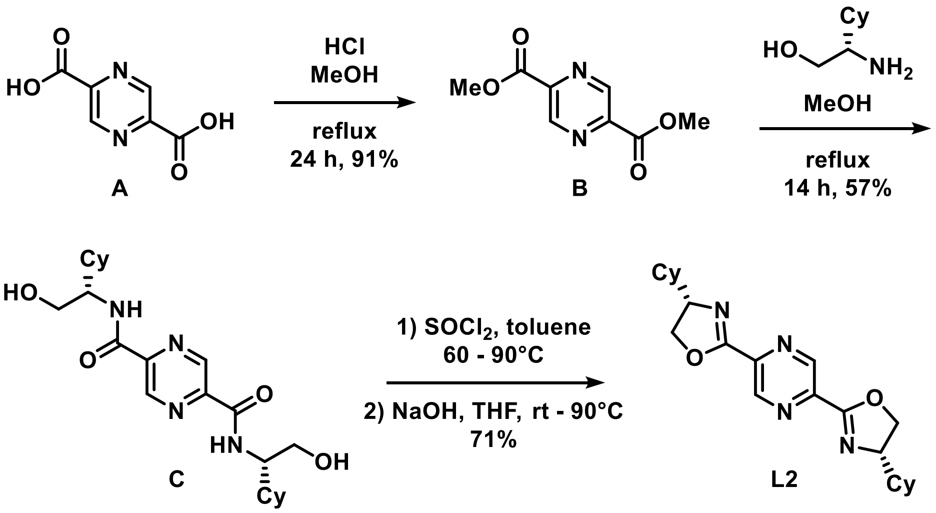

Figure SI1 – Synthesis of ligand **L2**.

Esterification: The diacid A (1.00 g, 5.95 mmol) was dissolved in 17 mL of MeOH. Then, 0.5 mL HCl 37% (w/w) was added and the mixture was heated to reflux for 24 h. At the end of this period, the solvent was evaporated and the residue dissolved in 25 mL of saturated $NaHCO_3$ solution. This solution was washed with 3 × 100 mL of dichloromethane and after drying with Na_2SO_4, filtration and evaporation provided 1.06 g (5.4 mmol, 91%) of diester B. This crude material was used directly in the next step.

Bisamide synthesis: A solution of the diester B (1.06 g, 5.4 mmol) and (*S*)-2-amino-2-cyclohexylethan-1-ol (1.62 g, 11.34 mmol) in 20 mL of MeOH was heated to reflux for 14 h. Next, the solvent was evaporated, the residue was rinsed with 2 × 15 mL of MeOH to furnish the bisamide C in 57% yield (1.29 g, 3.08 mmol). This crude material was used directly in the next step.

Oxazoline synthesis: The bisamide C (1.29 g, 3.08 mmol) was dissolved in 30 mL dry toluene under nitrogen atmosphere. After 5 minutes of stirring, distilled $SOCl_2$ (1.468 g, 12.32 mmol, 0.90 mL) was added slowly. The temperature was adjusted to 60 °C and the reaction was stirred for 1 h, followed by 4 h at 110 °C. After cooling to room temperature, the solvent was evaporated, and the crude was dissolved in dichloromethane

and 15 mL of an aqueous solution of KOH 20% were added under stirring. The organic phase was stored, and the aqueous phase was further extracted with dichloromethane. The combined organic phases were washed with brine, dried over Na_2SO_4 and the solvent was removed under vacuum. The crude was then dissolved in 60 mL of THF, followed by addition of an ethanolic solution of NaOH (0.300 g in 16 mL of ethanol) and the reaction was stirred for 30 minutes at room temperature and for more 3 h at 90 °C. After evaporation of the solvent, the crude was redissolved in dichloromethane, and the organic phase was washed with $NaHCO_{3(sat)}$ solution and then separated in a separation funnel. The aqueous phase was washed with dichloromethane, and the combined organic phases were washed with brine, dried over Na_2SO_4 and concentrated in vacuum. The product was purified by column chromatography (AcOEt) and ligand **L2** was obtained as a yellowish solid (0.842 g, 2.20 mmol, 71%).

2,5-Bis((*S*)-4-cyclohexyl-4,5-dihydrooxazol-2-yl)pyrazine (L2)

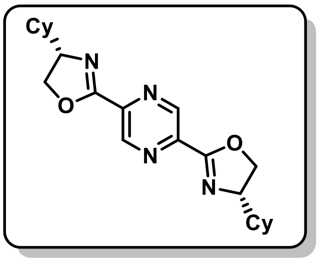

842 mg, 2.20 mmol, 37% yield. Yellowish solid.

^{1}H NMR (600 MHz, $CDCl_3$) δ 9.27 (s, 2H), 4.52 (dd, J = 9.7, 8.2 Hz, 2H), 4.26 (t, J = 8.5 Hz, 2H), 4.23 – 4.16 (m, 2H), 2.16 – 0.90 (m, 22H).

^{13}C NMR (63 MHz, $CDCl_3$) δ 160.45, 144.45, 143.50, 72.60, 71.29, 42.78, 29.74, 28.99, 26.53, 26.07.

$[\alpha]^{20}_D$ = -48 (c 0.10, MeOH)

HRMS (ESI+) *m*/*z* calculated for $C_{22}H_{30}N_4O_2+H^+$ $[M+H]^+$ 383.24415, found 383.24403.

mp: 144-145°C.

3. General procedure for the enantioselective Heck–Matsuda reactions and characterization

3.1 Considerations on the experimental procedure

During the development of the scope, the hemiaminal ethers (Heck–Matsuda products) were found to be somehow unstable when concentrated to dryness during work-up. We hypothesize that a possible cause of such instability might consist in the formation of a highly electrophilic iminium ion upon protonation of the hemiaminal ether by silica or glassware acidity and further elimination of methanol favored by the evaporation process. Although we found that careful control of the drying conditions avoids complete drying of the crude mixture prevents degradation of the Heck products, we established a robust protocol consisting of successive additions of acetone to the crude mixture, followed by careful rotaevaporation. This procedure gradually removes most of the methanol, allowing the sequential Jones oxidation step to take place without any significant losses. For clarity, the steps of the procedure are depicted in Figure SI2 to SI7.

NOTE: General Procedure (i) and **(ii)** differ only in the total volume of methanol added to the reaction (1.0 and 1.5 mL, respectively).

3.2 General procedure (i) – from olefins **1a** and **1b**

$Pd(TFA)_2$ (4.99 mg, 5 mol %, 0.015 mmol), ligand (*S*)-PyraBox (**L1**, 5.95 mg, 6 mol %, 0.018 mmol), and methanol (0.7 mL) were added to a 4 mL screw-top vial containing a magnetic stirrer. The resulting light-orange solution was then stirred at 40 °C for 15 min to form the catalyst complex. After cooling to room temperature, it was added $ZnCO_3$ (0.15 mmol, 18.8 mg, 0.5 equiv), the olefin **1a** or **1b** (0.30 mmol), the diazonium salt (0.60 mmol, 2 equiv) and the vial rinsed with MeOH (0.3 mL). The vial was then capped (not tightly to allow the release of N_2), and stirred for 4 h at 40 °C. The reaction vessel was then cooled to room temperature, and the mixture was poured onto a pad of silica gel (230–400 mesh; column height ≈ 4 cm; diameter ≈ 2 cm) previously washed with EtOAc/hexanes 1:1 mixture. The silica pad was then eluted with about 50 mL of 1:1 EtOAc/hexanes. The crude was then concentrated to approximately 0.5 mL, followed by the addition of 30 mL of acetone. The crude was concentrated again to ≈ 0.5 mL, followed by another addition of 30 mL of acetone. Finally, the crude was concentrated again to ≈ 0.5 mL, transferred to a 15 mL vial, the crude into dissolved in 6 mL of acetone:H_2O 3:1 ratio. Next, 1.0 mL of the Jones' reagent (2.5 M) was added and the reaction stirred for 90 minutes at rt. After this period, 2.0 mL of iPrOH were added and the reaction stirred for another 15 minutes. The solvent was removed under reduced pressure and the reaction mixture was poured onto a pad of silica gel (230–400 mesh; column height ≈ 1.5 cm; diameter ≈ 2 cm) having anhydrous Na_2SO_4 on top of it (≈ 1.5 cm). The pad was then washed with ≈ 50 mL of 1:1 EtOAc/hexanes. The solvent was then rotaevaporated, and the crude product was purified by column chromatography on silica gel with EtOAc/ hexanes as the eluent to provide the corresponding lactams.

3.3 General procedure (ii) – from olefins **1c** and **1d**

The same procedure as above, but with a slight difference in its first part regarding the volume of MeOH needed to solubilize the nosylated pyrrolines **1c**, and **1d**, as follows: $Pd(TFA)_2$ (4.99 mg, 5 mol %, 0.015 mmol), ligand

(S)-PyraBox (**L1**, 5.95 mg, 6 mol %, 0.018 mmol), and methanol (0.7 mL) were added to a 4 mL screw-top vial containing a magnetic stirrer. The resulting light-orange solution was then stirred at 40 °C for 15 min to form the catalyst complex. After cooling to room temperature, it was added $ZnCO_3$ (0.15 mmol, 18.8 mg, 0.5 equiv), the olefin **1a** or **1b** (0.30 mmol), the diazonium salt (0.60 mmol, 2 equiv) and the vial rinsed with MeOH (0.8 mL).

3.4 Illustration of the general procedures:

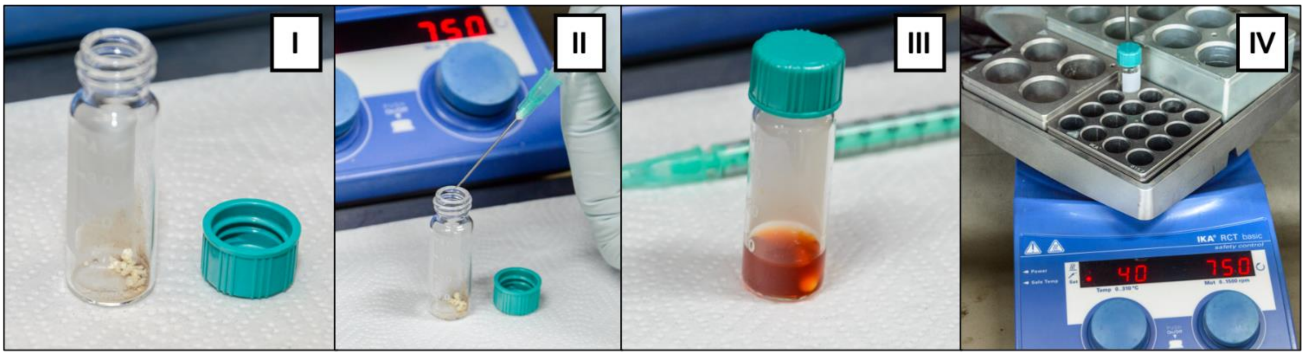

Figure SI2 - (I) Weighting of $Pd(TFA)_2$ and (S)-PyraBox ligand (**L1**); (II) addition of MeOH; (III) light-orange solution resulting from solubilization of $Pd(TFA)_2$ and ligand **L1**; (IV) formation of the catalyst complex upon heating at 40 °C for 15 min.

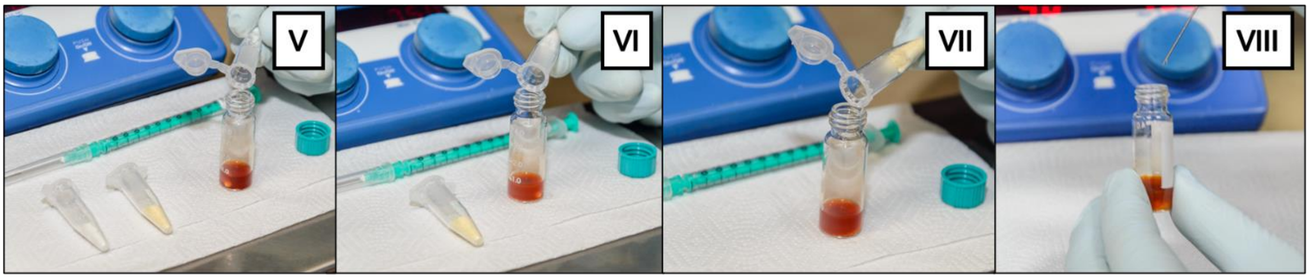

Figure SI3 - (V) Addition of $ZnCO_3$ to the catalyst solution; (VI) addition of the olefin; (VII) addition of diazonium salt; (VIII) rinsing the vial with MeOH.

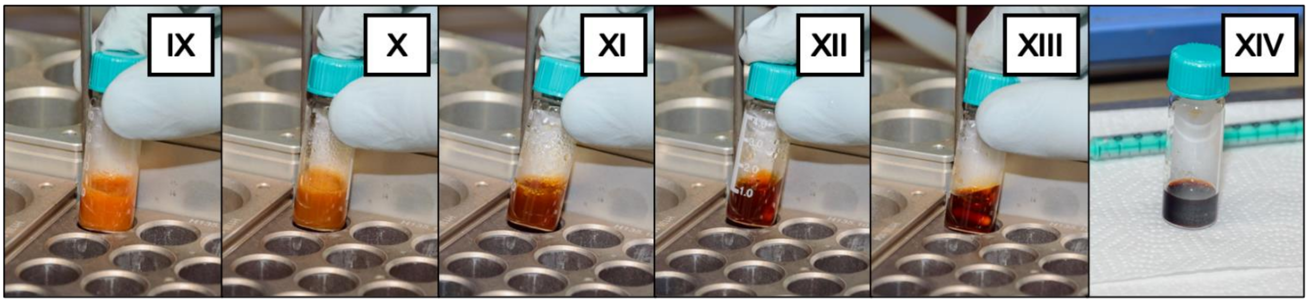

Figure SI4 - Solubilization of the starting materials over time: (IX) Heck–Matsuda reaction t = 0 min; (X) t = 5 min; (XI) t = 10 min; (XII) t = 15 min; (XIII) t = 60 min; (XIV) t = 240 min – end of the reaction. Although colors may vary with the diazonium salt used, the end of the reaction is indicated by the dark reddish black mixture.

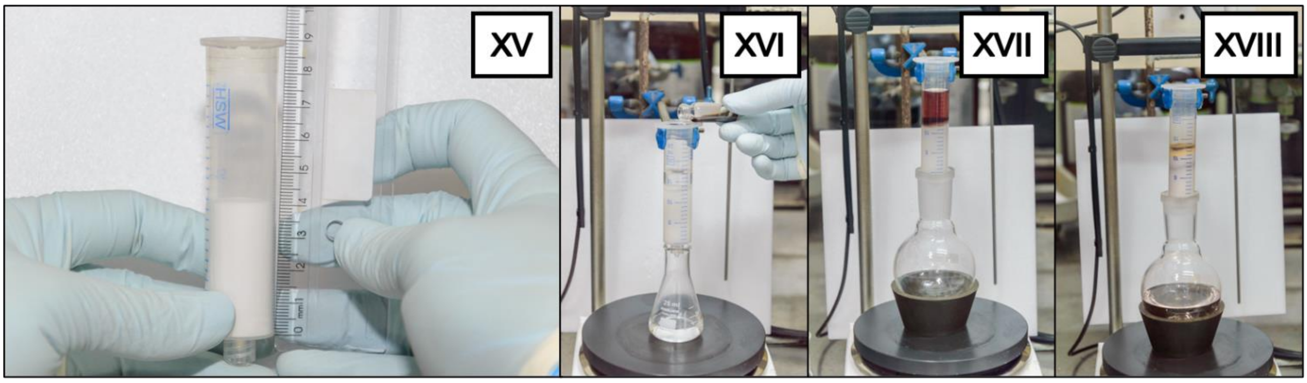

Figure SI5 - Work up procedure for the Heck–Matsuda reaction: (XV) silica pad (height: 4 cm, diameter: 2 cm; (XVI) pouring of the reaction onto the pre-conditioned silica pad; (XVII) elution; (XVIII) diluted reaction media after elution.

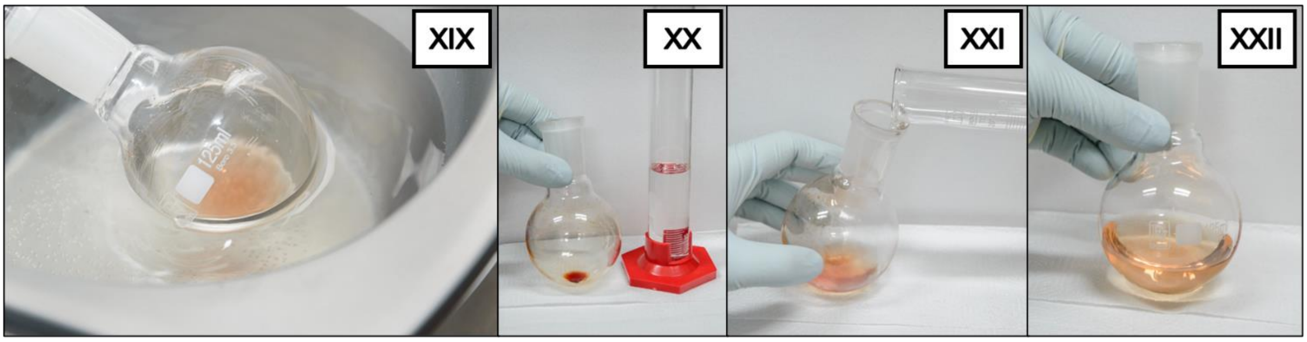

Figure SI6 - Critical evaporation step. (XIX) diluted reaction media concentration on rotatory evaporator; (XX) concentrated (not completely dry!) until approximately 0.5 mL; (XXI) addition of 30 mL of acetone to the concentrated crude; (XXII) crude diluted in acetone ready for another evaporation step.

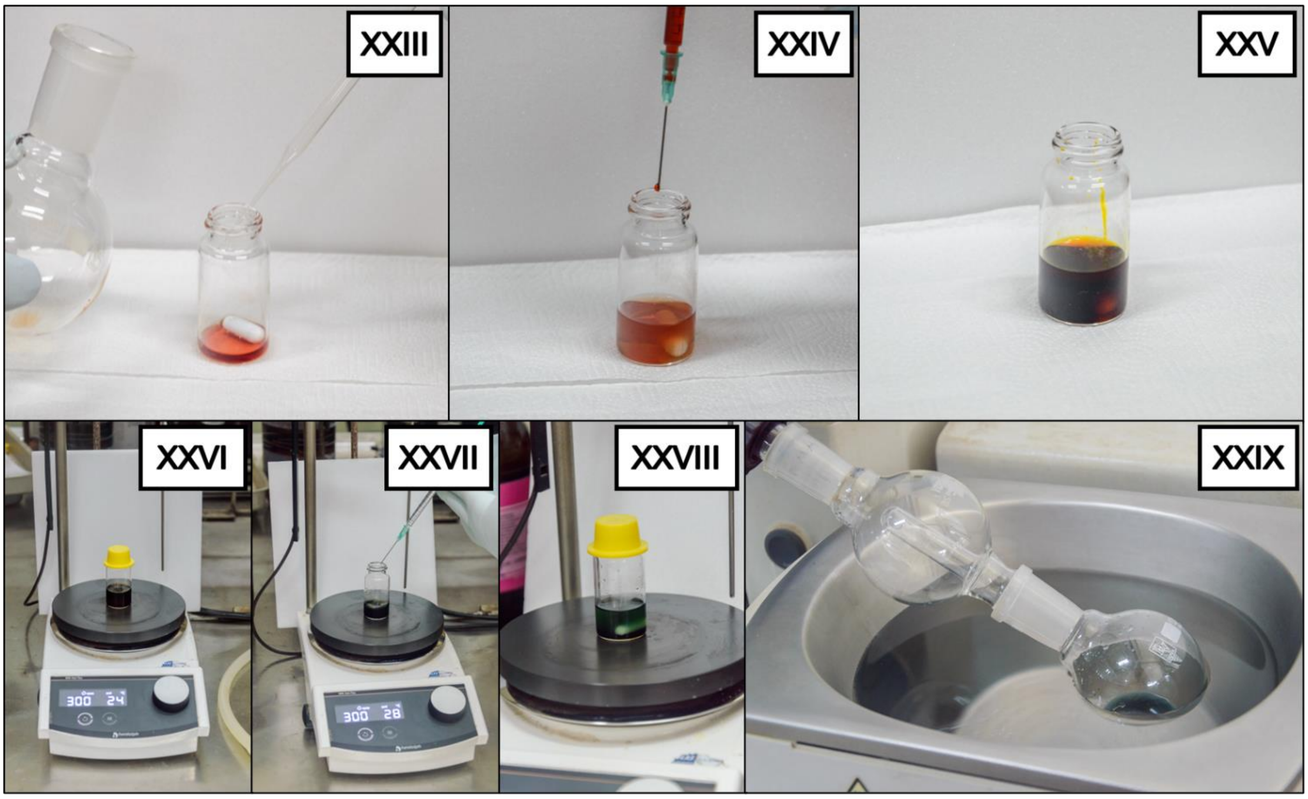

Figure SI7 - Jones oxidation reaction setup: (XXIII) concentrated crude reaction media transference to a 15 mL vial with acetone:H_2O 4:1 (v/v); (XXIV) addition of the Jones solution 2.5 M; (XXV) reaction media after Jones solution addition; (XXVI) Jones oxidation in progress; (XXVII) addition of iPrOH by the end of reaction; (XXVIII) reaction media after deactivation of Jones solution by reduction of Cr(VI) to Cr(III); (XXIX) concentration of the crude reaction media in rotatory evaporation (no extra precautions needed at this point).

4 Characterization of products

***tert*-Butyl (*R*)-4-(4-methoxyphenyl)-2-oxopyrrolidine-1-carboxylate (4aa)** [9]

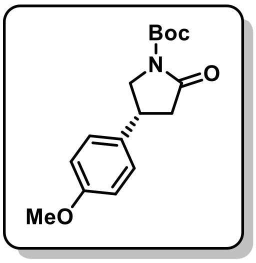

Following General Procedure (i). Colorless oil.
(59.3 mg, 0.204 mmol, 68% yield). 78:22 *er.*
The enantiomeric ratio was determined by SFC analysis in comparison to a racemic material using Daicel Chiralpak® IC column (4.6 mm × 250 mm) at 30 °C and 10% MeOH in supercritical CO_2 (3.0 mL min^{-1}) as mobile phase, λ = 230 nm (t_r = 7.9 min (minor), 9.3 min (major).

^{1}H NMR (400 MHz, $CDCl_3$) δ 7.14 (d, J = 8.7 Hz, 2H), 6.87 (d, J = 8.7 Hz, 2H), 4.11 (dd, J = 10.7, 8.1 Hz, 1H), 3.78 (s, 3H), 3.62 (dd, J = 10.8, 8.6 Hz, 1H), 3.47 (quint, J = 8.0 Hz, 1H), 2.84 (dd, J = 17.2, 8.4 Hz, 1H), 2.65 (dd, J = 17.2, 10.0 Hz, 1H), 1.51 (s, 9H).

^{13}C NMR (101 MHz, CDCl3) δ 173.2, 158.9, 149.9, 132.6, 127.8, 114.4, 83.0, 55.4, 53.4, 40.6, 35.8, 28.1.

HRMS (ESI+) m/z calculated for $C_{16}H_{21}NO_4$+Na^+ $[M+Na]^+$ 314.13628, found 314.13556.

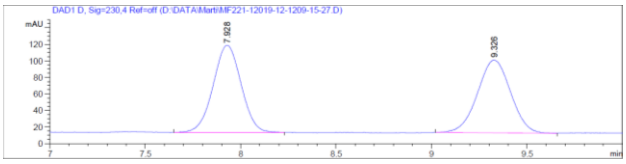

Signal 3: DAD1 D, Sig=230,4 Ref=off

Peak #	RetTime [min]	Type	Width [min]	Area [mAU*s]	Height [mAU]	Area %
1	7.928	BB	0.1542	1073.96216	105.52740	50.2413
2	9.326	BB	0.1718	1063.64502	88.12798	49.7587
Totals :				2137.60718	193.65539	

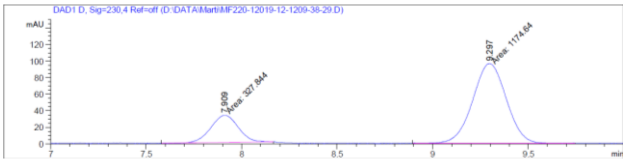

Signal 3: DAD1 D, Sig=230,4 Ref=off

Peak #	RetTime [min]	Type	Width [min]	Area [mAU*s]	Height [mAU]	Area %
1	7.909	MM	0.1670	327.84363	32.72367	21.8200
2	9.297	MM	0.2036	1174.64441	96.14930	78.1800
Totals :				1502.48804	128.87297	

***tert*-Butyl (*R*)-4-phenyl-2-oxopyrrolidine-1-carboxylate (4ab)** [9]

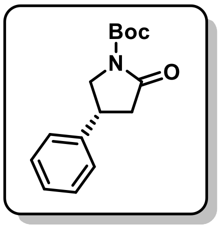

Following General Procedure (i). Colorless oil.

27.0 mg, 0.102 mmol, 34% yield, 80:20 *er.*

The enantiomeric ratio was determined by SFC analysis in comparison to a racemic material using Daicel Chiralpak® IC column (4.6 mm × 250 mm) at 30 °C and 10% MeOH in supercritical CO_2 (3.0 mL min^{-1}) as mobile phase, λ = 210 nm (t_r = 5.2 min (minor), 6.2 min (major)).

^{1}H NMR (400 MHz, $CDCl_3$) δ 7.40 – 7.36 (m, 2H), 7.33 – 7.24 (m, 3H), 4.16 (dd, *J* = 10.8, 8.1 Hz, 1H), 3.69 (dd, *J* = 10.8, 8.5 Hz, 1H), 3.53 (quint, *J* = 8.0 Hz, 1H), 2.89 (dd, *J* = 17.3, 8.5 Hz, 1H), 2.71 (dd, *J* = 17.3, 9.9 Hz, 1H), 1.53 (s, 9H).

^{13}C NMR (101 MHz, $CDCl_3$) δ 173.1, 150.0, 140.7, 129.1, 127.5, 126.9, 83.2, 53.2, 40.4, 36.5, 28.1.

HRMS (ESI+) *m*/*z* calculated for $C_{15}H_{19}NO_3$+Na^+ [M+Na]$^+$ 284.12571, found 284.12505.

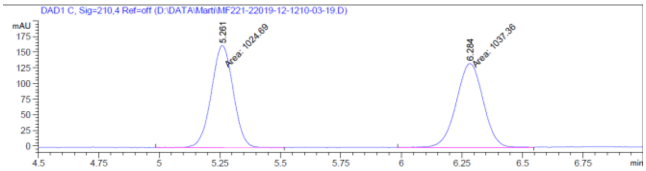

Signal 2: DAD1 C, Sig=210,4 Ref=off

Peak #	RetTime [min]	Type	Width [min]	Area [mAU*s]	Height [mAU]	Area %
1	5.261	MM	0.1048	1024.68652	162.98163	49.6926
2	6.284	MM	0.1289	1037.36316	134.12086	50.3074
Totals :				2062.04968	297.10249	

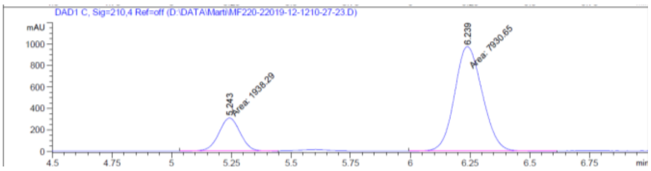

Signal 2: DAD1 C, Sig=210,4 Ref=off

Peak #	RetTime [min]	Type	Width [min]	Area [mAU*s]	Height [mAU]	Area %
1	5.243	MM	0.1048	1938.29065	308.36526	19.6403
2	6.239	MM	0.1356	7930.64600	975.07837	80.3597
Totals :				9868.93665	1283.44363	

***tert*-Butyl (*R*)-2-oxo-4-(4-(trifluoromethyl)phenyl)pyrrolidine-1-carboxylate (4ac)** [9]

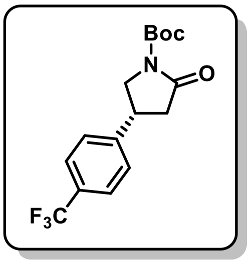

Following General procedure (i). Clear yellow oil.

26.6 mg, 0.081 mmol, 27% yield, 80:20 *er*.

The enantiomeric ratio was determined by SFC analysis in comparison to a racemic material using Daicel Chiralpak® IC column (4.6 mm × 250 mm) at 30 °C and 10% MeOH in supercritical CO_2 (3.0 mL min^{-1}) as mobile phase, λ = 210 nm (t_r = 3.0 min (minor), 3.5 min (major)).

^{1}H NMR (400 MHz, CDCl$_3$) δ 7.62 (d, *J* = 8.2 Hz, 2H), 7.36 (d, *J* = 8.2 Hz, 2H), 4.19 (dd, *J* = 10.7, 8.0 Hz, 1H), 3.70 (dd, *J* = 10.8, 8.2 Hz, 1H), 3.60 (quint, *J* = 8.0 Hz, 1H), 2.94 (dd, *J* = 17.3, 8.4 Hz, 1H), 2.71 (dd, *J* = 17.3, 9.5 Hz, 1H), 1.53 (s, 9H).

^{13}C NMR (101 MHz, CDCl$_3$) δ 172.5, 149.9, 144.8, 130.47, 130.0 (q, *J* = 32.3 Hz), 127.3, 126.1 (q, *J* = 4.0 Hz), 124.0 (q, *J* = 273.7 Hz), 83.5, 52.8, 40.2, 36.3, 28.1.

HRMS (ESI+) *m*/*z* calculated for $C_{16}H_{18}F_3NO_3+Na^+$ $[M+Na]^+$ 352.11310, found 352.11236.

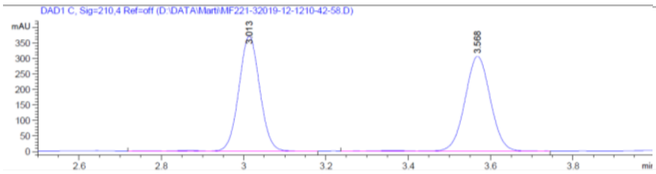

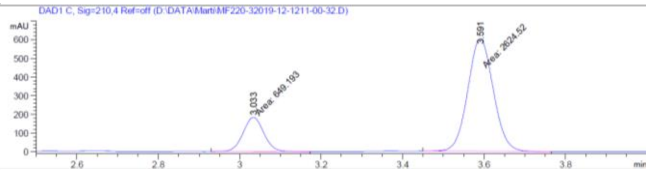

Signal 2: DAD1 C, Sig=210,4 Ref=off

Peak #	RetTime [min]	Type	Width [min]	Area [mAU*s]	Height [mAU]	Area %
1	3.013	VB R	0.0550	1315.82117	369.77844	50.0216
2	3.568	VV R	0.0668	1314.68652	305.09518	49.9784
Totals :				2630.50769	674.87363	

Signal 2: DAD1 C, Sig=210,4 Ref=off

Peak #	RetTime [min]	Type	Width [min]	Area [mAU*s]	Height [mAU]	Area %
1	3.033	MM	0.0590	649.19318	183.26735	19.8305
2	3.591	MM	0.0724	2624.51953	603.75623	80.1695
Totals :				3273.71271	787.02357	

(*R*)-4-(4-Methoxyphenyl)-1-tosylpyrrolidin-2-one (4ba)

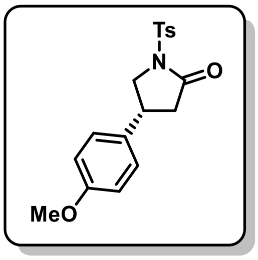

Following General Procedure (i). Pale yellow sticky solid.

Run 1: (88.6 mg, 0.255 mmol, 85% yield). **Run 2:** (88.0 mg, 0.255 mmol, 85% yield).

Average: 85% yield

The enantiomeric ratio was determined by HPLC analysis in comparison to a racemic material using Daicel Chiralpak® IC column (4.6 mm × 250 mm) at 30 °C and 60:40 hexanes:iPrOH (1.3 mL min^{-1}) as mobile phase, λ = 225 nm (t_r = 40.1 min (major), 51.2 min (minor)).

^{1}H NMR (500 MHz, CDCl$_3$) δ 7.93 (d, *J* = 8.3 Hz, 2H), 7.34 (d, *J* = 8.1 Hz, 2H), 7.05 (d, *J* = 8.6 Hz, 2H), 6.84 (d, *J* = 8.6 Hz, 2H), 4.29 (dd, *J* = 9.8, 8.0 Hz, 1H), 3.79 (s, 3H), 3.74 (dd, *J* = 9.8, 8.2 Hz, 1H), 3.55 (quint, *J* = 8.3 Hz, 1H), 2.80 (dd, *J* = 17.3, 8.4 Hz, 1H), 2.57 (dd, *J* = 17.3, 9.4 Hz, 1H), 2.45 (s, 3H).

^{13}C NMR (126 MHz, CDCl$_3$) δ 172.3, 159.1, 145.4, 135.8, 131.3, 129.9, 128.2, 127.8, 114.5, 55.5, 54.0, 39.9, 36.7, 21.8.

$[\alpha]^{20}_D$ = -19 (*c* 0.98, CHCl$_3$, 84:16 er)

HRMS (ESI+) *m*/*z* calculated for $C_{18}H_{19}NO_4S+H^+$ $[M+H]^+$ 346.11076, found 346.11031.

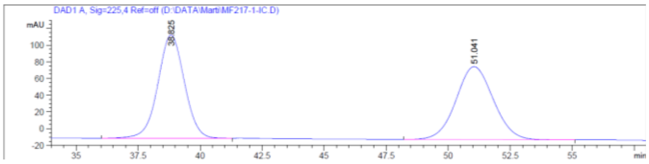

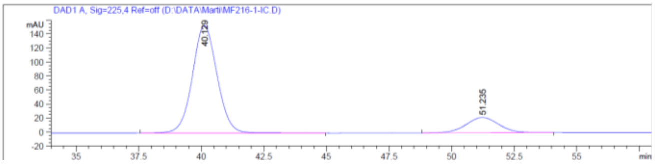

Signal 1: DAD1 A, Sig=225,4 Ref=off

Peak #	RetTime [min]	Type	Width [min]	Area [mAU*s]	Height [mAU]	Area %
1	38.825	BB	1.1342	9156.52148	123.61306	49.9561
2	51.041	BB	1.5869	9172.61328	87.36315	50.0439
Totals :				1.83291e4	210.97621	

Signal 1: DAD1 A, Sig=225,4 Ref=off

Peak #	RetTime [min]	Type	Width [min]	Area [mAU*s]	Height [mAU]	Area %
1	40.129	BB	1.0507	1.05005e4	153.37325	84.3914
2	51.235	BB	1.1936	1942.12158	21.58761	15.6086
Totals :				1.24426e4	174.96085	

(*R*)-4-Phenyl-1-tosylpyrrolidin-2-one (4bb)

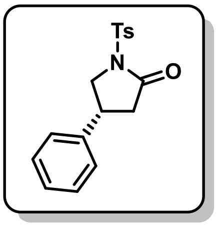

Following General Procedure (i). Colorless oil.

Run 1: (67.1 mg, 0.213 mmol, 71% yield). **Run 2:** (69.3 mg, 0.219 mmol, 73% yield). **Average: 72% yield.**

The enantiomeric ratio was determined by HPLC analysis in comparison to a racemic material using Daicel Chiralpak® IC column (4.6 mm × 250 mm) at 30 °C and 60:40 hexanes:iPrOH (1.3 mL min^{-1}) as mobile phase, λ = 225 nm (t_r = 24.3 min (major), 37.9 min (minor)).

^{1}H NMR (500 MHz, $CDCl_3$) δ 7.93 (d, *J* = 8.2 Hz, 2H), 7.38 – 7.26 (m, 5H), 7.14 (d, *J* = 7.1 Hz, 2H), 4.33 (dd, *J* = 9.8, 8.1 Hz, 1H), 3.79 (dd, *J* = 9.8, 8.1 Hz, 1H), 3.60 (quint, *J* = 8.3 Hz, 1H), 2.84 (dd, *J* = 17.3, 8.4 Hz, 1H), 2.61 (dd, *J* = 17.3, 9.3 Hz, 1H), 2.45 (s, 3H).

^{13}C NMR (126 MHz, $CDCl_3$) δ 172.2, 145.4, 139.9, 135.2, 129.9, 129.2, 128.2, 127.8, 126.7, 53.8, 39.6, 37.3, 21.8.

$[\alpha]^{20}_{D}$ = -12 (*c* 1.05, $CHCl_3$, 79:21 *er*)

HRMS (ESI+) *m/z* calculated for $C_{17}H_{17}NO_3S+Na^+$ $[M+Na]^+$ 338.08214, found 338.08137.

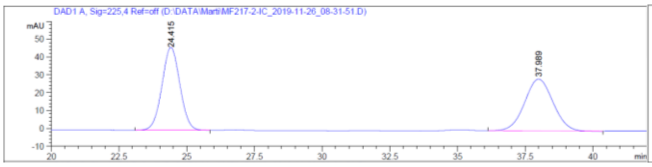

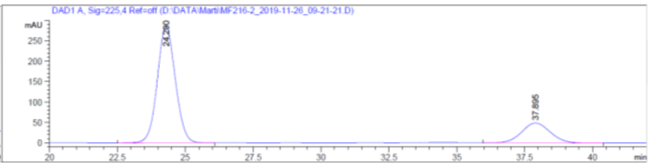

Signal 1: DAD1 A, Sig=225,4 Ref=off

Peak #	RetTime [min]	Type	Width [min]	Area [mAU*s]	Height [mAU]	Area %
1	24.415	BB	0.7101	2134.50488	46.45018	50.1794
2	37.989	BB	1.0867	2119.23975	28.99480	49.8206
Totals :				4253.74463	75.44498	

Signal 1: DAD1 A, Sig=225,4 Ref=off

Peak #	RetTime [min]	Type	Width [min]	Area [mAU*s]	Height [mAU]	Area %
1	24.290	BB	0.7149	1.31630e4	284.98715	78.6391
2	37.895	BB	1.0946	3575.48438	48.81356	21.3609
Totals :				1.67384e4	333.80071	

(*R*)-1-Tosyl-4-(4-(trifluoromethyl)phenyl)pyrrolidin-2-one (4bc)

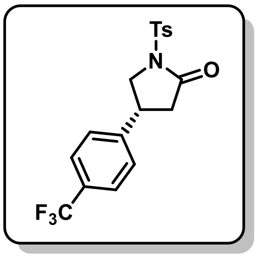

Following General Procedure (i). Yellow oil.

Run 1: (71.6 mg, 0.186 mmol, 62% yield). **Run 2:** (76.3 mg, 0.198 mmol, 66% yield). **Average: 64% yield.**

The enantiomeric ratio was determined by HPLC analysis in comparison to a racemic material using Daicel Chiralpak® IC column (4.6 mm × 250 mm) at 30 °C and 60:40 hexanes:iPrOH (1.3 mL min^{-1}) as mobile phase, λ = 225 nm (t_r = 15.8 min (major), 20.5 min (minor)).

^{1}H NMR (400 MHz, CDCl$_3$) δ 7.92 (d, *J* = 8.3 Hz, 2H), 7.56 (d, *J* = 8.1 Hz, 2H), 7.34 (d, *J* = 8.1 Hz, 2H), 7.26 (d, *J* = 8.1 Hz, 2H), 4.35 (dd, *J* = 10.0, 7.8 Hz, 1H), 3.80 (dd, *J* = 10.0, 7.4 Hz, 1H), 3.66 (quint, *J* = 8.3 Hz, 1H), 2.89 (dd, *J* = 17.3, 8.4 Hz, 1H), 2.60 (dd, *J* = 17.3, 8.6 Hz, 1H), 2.46 (s, 3H).

^{13}C NMR (101 MHz, CDCl$_3$) δ 171.6, 145.6, 144.2, 135.1, 129.9, 128.2, 127.1, 126.7 (q, *J* = 272.7 Hz) 126.2 (q, *J* = 3.7 Hz), 53.4, 39.4, 37.0, 21.8.

$[\alpha]^{20}_D$ = -14 (*c* 0.97, CHCl$_3$, 85:15 *er*)

HRMS (ESI+) *m/z* calculated for $C_{18}H_{16}F_3NO_3S+H^+$ $[M+H]^+$ 384.08758, found 384.08681.

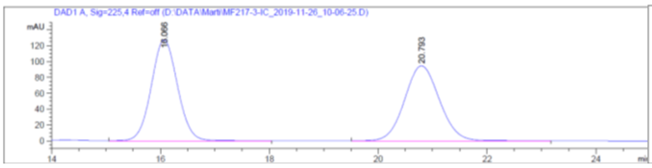

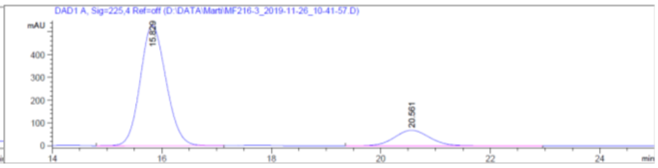

Signal 1: DAD1 A, Sig=225,4 Ref=off

Peak #	RetTime [min]	Type	Width [min]	Area [mAU*s]	Height [mAU]	Area %
1	16.066	BB	0.5168	4255.62500	127.70363	50.2258
2	20.793	BB	0.6891	4217.36084	94.79144	49.7742
Totals :				8472.98584	222.49506	

Signal 1: DAD1 A, Sig=225,4 Ref=off

Peak #	RetTime [min]	Type	Width [min]	Area [mAU*s]	Height [mAU]	Area %
1	15.829	BB	0.5066	1.72516e4	528.83960	84.9903
2	20.561	BB	0.6892	3046.71436	68.46552	15.0097
Totals :				2.02983e4	597.30511	

(*R*)-Methyl 4-(5-oxo-1-tosylpyrrolidin-3-yl)benzoate (4bd)

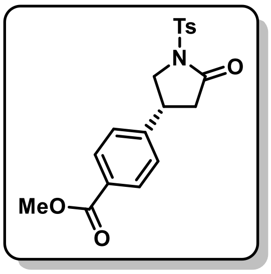

Following General Procedure (i). Yellow solid.

Run 1: (86.5 mg, 0.231 mmol, 77% yield). **Run 2:** (84.6 mg, 0.226 mmol, 75% yield).

Average: 76% yield.

The enantiomeric ratio was determined by HPLC analysis in comparison to a racemic material using Daicel Chiralpak® IB column (4.6 mm × 250 mm) at 30 °C and 80:20 hexanes:iPrOH (1.3 mL min^{-1}) as mobile phase, λ = 225 nm (t_r = 23.3 min (minor), 25.1 min (major)).

^{1}H NMR (400 MHz, CDCl$_3$) δ 7.95 (d, *J* = 8.4 Hz, 2H), 7.90 (d, *J* = 8.3 Hz, 2H), 7.32 (d, *J* = 8.1 Hz, 2H), 7.19 (d, *J* = 8.3 Hz, 2H), 4.33 (dd, *J* = 10.0, 7.8 Hz, 1H), 3.89 (s, 3H), 3.78 (dd, *J* = 10.0, 7.6 Hz, 1H), 3.69 – 3.59 (m, 1H), 2.86 (dd, *J* = 17.3, 8.4 Hz, 1H), 2.59 (dd, *J* = 17.3, 8.8 Hz, 1H), 2.43 (s, 3H).

^{13}C NMR (101 MHz, CDCl$_3$) δ 171.8, 166.6, 145.5, 145.2, 135.0, 130.4, 129.8, 129.6, 128.1, 126.7, 53.3, 52.3, 39.3, 37.1, 21.7.

$[\alpha]^{20}_D$ = -18 (*c* 1,00, CHCl$_3$, 87:13 *er*)

HRMS (ESI+) *m/z* calculated for $C_{19}H_{19}NO_5S+H^+$ $[M+H]^+$ 374.10567, found 374.10530.

HRMS (ESI+) *m/z* calculated for $C_{19}H_{19}NO_5S+Na^+$ $[M+Na]^+$ 396.08761, found 396.0873.

mp: 105-107°C.

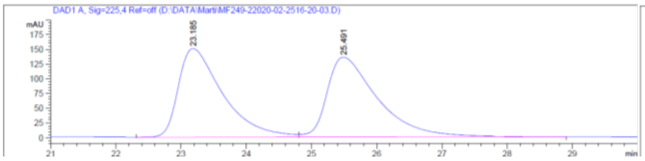

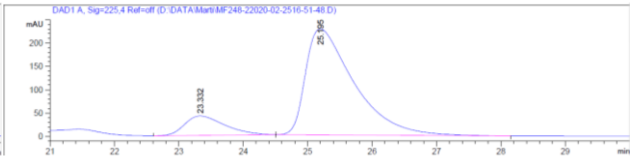

Signal 1: DAD1 A, Sig=225,4 Ref=off

Peak #	RetTime [min]	Type	Width [min]	Area [mAU*s]	Height [mAU]	Area %
1	23.185	BV	0.7013	7113.47754	150.52448	49.3431
2	25.491	VB	0.7939	7302.87891	135.69852	50.6569

Totals : 1.44164e4 286.22299

Signal 1: DAD1 A, Sig=225,4 Ref=off

Peak #	RetTime [min]	Type	Width [min]	Area [mAU*s]	Height [mAU]	Area %
1	23.332	BB	0.6532	1793.75281	41.58822	13.1407
2	25.195	BBA	0.7715	1.18566e4	226.97035	86.8593

Totals : 1.36504e4 268.55857

(*R*)-4-(4-Acetylphenyl)-1-tosylpyrrolidin-2-one (4be)

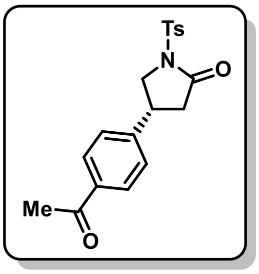

Following General Procedure (i). Yellow oily solid.

Run 1: (78.1 mg, 0.216 mmol, 72% yield). **Run 2:** (75.9 mg, 0.212 mmol, 70% yield). **Average: 71% yield.**

The enantiomeric ratio was determined by HPLC analysis in comparison to a racemic material using Daicel Chiralpak® IB column (4.6 mm × 250 mm) at 30 °C and 70:30 hexanes:iPrOH (1.3 mL min^{-1}) as mobile phase, λ = 225 nm (t_r = 21.5 min (minor), 24.5 min (major)).

^{1}H NMR (400 MHz, CDCl$_3$) δ 7.93 - 7.86 (m, 4H), 7.33 (d, *J* = 8.1 Hz, 2H), 7.23 (d, *J* = 8.3 Hz, 2H), 4.33 (dd, *J* = 10.0, 7.9 Hz, 1H), 3.80 (dd, *J* = 10.0, 7.6 Hz, 1H), 3.66 (quint, *J* = 8.2 Hz, 1H), 2.86 (dd, *J* = 17.3, 8.5 Hz, 1H), 2.60 (m, 1H), 2.57 (s, 3H), 2.44 (s, 3H).

^{13}C NMR (101 MHz, CDCl$_3$) δ 197.5, 171.7, 145.6, 145.4, 136.5, 135.0, 129.9, 129.2, 128.2, 126.9, 53.3, 39.3, 37.1, 26.7, 21.8.

$[\alpha]^{20}_D$ = -33 (*c* 1.02, CHCl$_3$, 87:13 *er*)

HRMS (ESI+) *m/z* calculated for $C_{19}H_{19}NO_4S+H^+$ $[M+H]^+$ 358.11076, found 358.11035.

mp: 94-96°C.

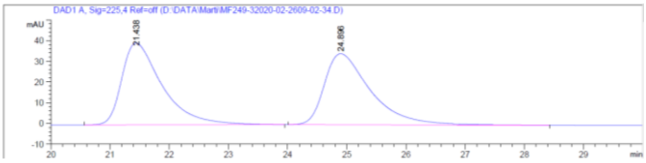

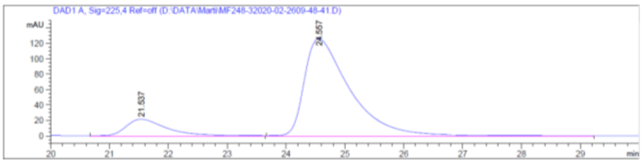

Signal 1: DAD1 A, Sig=225,4 Ref=off

Peak #	RetTime [min]	Type	Width [min]	Area [mAU*s]	Height [mAU]	Area %
1	21.438	BB	0.7080	1873.16040	39.44936	50.1703
2	24.896	BB	0.7972	1860.44543	34.27748	49.8297

Totals : 3733.60583 73.72684

Signal 1: DAD1 A, Sig=225,4 Ref=off

Peak #	RetTime [min]	Type	Width [min]	Area [mAU*s]	Height [mAU]	Area %
1	21.537	BB	0.6967	1018.51294	21.50101	13.1215
2	24.557	BB	0.7952	6743.64111	125.83127	86.8785

Totals : 7762.15405 147.33228

(*R*)-1-Tosyl-4-(4-fluorophenyl)pyrrolidin-2-one (4bf)

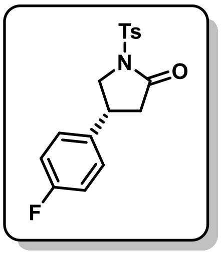

Following General Procedure (i). Colorless solid.

Run 1: (76.5 mg, 0.228 mmol, 76% yield). **Run 2:** (76.1 mg, 0.212 mmol, 76% yield). **Average: 76% yield.**

The enantiomeric ratio was determined by HPLC analysis in comparison to a racemic material using Daicel Chiralpak® IC column (4.6 mm × 250 mm) at 30 °C and 50:50

hexanes:iPrOH (1.3 mL min^{-1}) as mobile phase, λ = 230 nm (t_r = 14.8 min (major), 19.6 min (minor)).

^{1}H NMR (400 MHz, CDCl$_3$) δ 7.91 (d, *J* = 8.3 Hz, 2H), 7.34 (d, *J* = 8.1 Hz, 2H), 7.14 – 7.06 (m, 2H), 7.02 - 6.95 (m, 2H), 4.30 (dd, *J* = 9.9, 7.8 Hz, 1H), 3.74 (dd, *J* = 9.9, 7.7 Hz, 1H), 3.58 (quint, *J* = 8.2 Hz, 1H), 2.83 (dd, *J* = 17.3, 8.4 Hz, 1H), 2.55 (dd, *J* = 17.3, 9.0 Hz, 1H), 2.45 (s, 3H).

^{13}C NMR (101 MHz, CDCl$_3$) δ 171.9, 162.1 (d, *J* = 247.4 Hz), 145.5, 135.7 (d, *J* = 3.0 Hz), 135.1, 129.8, 128.3 (d, *J* = 8.0 Hz), 128.2, 116.0 (d, *J* = 21.2 Hz), 53.8, 39.7, 36.6, 21.8.

^{19}F NMR (235 MHz, CDCl$_3$) δ 114.45.

$[\alpha]^{20}_{D}$ = -17 (*c* 1.01, CHCl$_3$, 86:14 er)

HRMS (ESI+) *m/z* calculated for $C_{17}H_{16}FNO_3S+H^+$ $[M+H]^+$ 334.09077, found 334.09050.

HRMS (ESI+) *m/z* calculated for $C_{17}H_{16}FNO_3S$ +Na^+ $[M+Na]^+$ 356.07271, found 356.07220.

mp: 120-121°C.

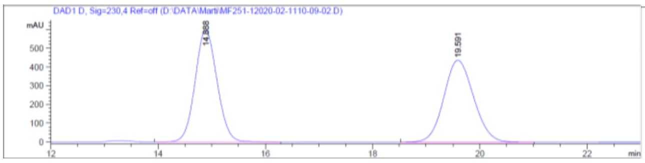

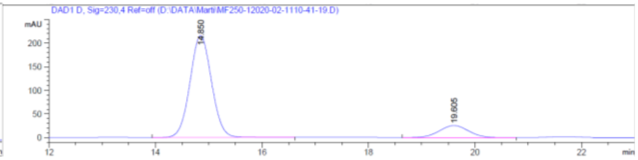

Signal 3: DAD1 D, Sig=230,4 Ref=off

Peak #	RetTime [min]	Type	Width [min]	Area [mAU*s]	Height [mAU]	Area %
1	14.888	BB	0.4390	1.69710e4	598.17249	50.2018
2	19.591	BB	0.5973	1.68346e4	437.10205	49.7982
Totals :				3.38056e4	1035.27454	

Signal 3: DAD1 D, Sig=230,4 Ref=off

Peak #	RetTime [min]	Type	Width [min]	Area [mAU*s]	Height [mAU]	Area %
1	14.850	BB	0.4361	6069.88184	215.81664	86.2683
2	19.605	BB	0.5940	966.16766	25.27286	13.7317
Totals :				7036.04950	241.08949	

(*R*)-1-Tosyl-4-(4-chlorophenyl)pyrrolidin-2-one (4bg)

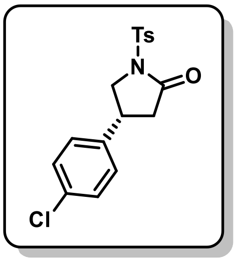

Following General Procedure (i). Clear-yellow gum.

Run 1: (72.0 mg, 0.204 mmol, 68% yield). **Run 2:** (76.5 mg, 0.219 mmol, 73% yield). **Average: 70% yield.**

The enantiomeric ratio was determined by HPLC analysis in comparison to a racemic material using Daicel Chiralpak® IC column (4.6 mm × 250 mm) at 30 °C and 50:50 hexanes:iPrOH (1.3 mL min^{-1}) as mobile phase, λ = 225 nm (t_r = 23.8 min (major), 30.1 min (minor)).

^{1}H NMR (500 MHz, CDCl$_3$) δ 7.83 (d, *J* = 8.3 Hz, 2H), 7.26 (d, *J* = 8.1 Hz, 2H), 7.19 (d, *J* = 8.4 Hz, 2H), 6.99 (d, *J* = 8.4 Hz, 2H), 4.23 (dd, *J* = 9.9, 7.9 Hz, 1H), 3.67 (dd, *J* = 9.9, 7.7 Hz, 1H), 3.50 (quint, *J* = 8.1 Hz, 1H), 2.76 (dd, *J* = 17.3, 8.4 Hz, 1H), 2.48 (dd, *J* = 17.3, 8.9 Hz, 1H), 2.38 (s, 3H).

^{13}C NMR (126 MHz, CDCl$_3$) δ 171.9, 145.5, 138.5, 135.0, 133.5, 129.8, 129.3, 128.2, 128.0, 53.6, 39.5, 36.7, 21.8.

$[\alpha]^{20}_{D}$ = -25 (*c* 1.05, CHCl$_3$, 86:14 *er*)

HRMS (ESI+) *m/z* calculated for $C_{17}H_{16}ClNO_3S+Na^+$ $[M+Na]^+$ 372.04316, found 372.04237.

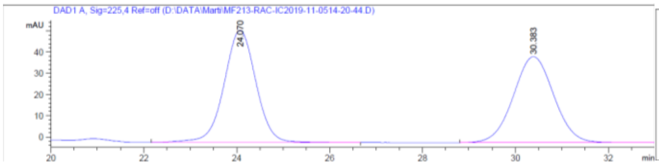

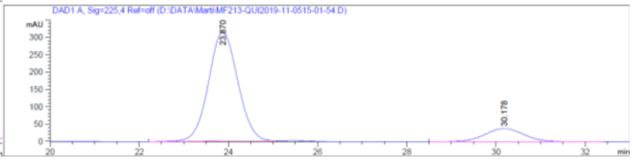

Signal 1: DAD1 A, Sig=225,4 Ref=off

Peak #	RetTime [min]	Type	Width [min]	Area [mAU*s]	Height [mAU]	Area %
1	24.070	BB	0.7278	2518.49512	52.86131	50.6226
2	30.383	BB	0.9379	2456.54688	40.60169	49.3774
Totals :				4975.04199	93.46300	

Signal 1: DAD1 A, Sig=225,4 Ref=off

Peak #	RetTime [min]	Type	Width [min]	Area [mAU*s]	Height [mAU]	Area %
1	23.870	BB	0.7032	1.42843e4	314.92059	86.2496
2	30.178	BB	0.9262	2277.29248	37.83499	13.7504
Totals :				1.65616e4	352.75558	

(*R*)-1-Tosyl-4-(4-bromophenyl)pyrrolidin-2-one (4bh)

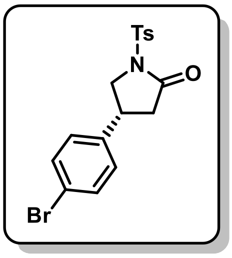

Following General Procedure (i). Yellow solid.

Run 1: (83.1 mg, 0.210 mmol, 70% yield). **Run 2:** (81.2 mg, 0.206 mmol, 68% yield). **Average: 69% yield.**

The enantiomeric ratio was determined by HPLC analysis in comparison to a racemic material using Daicel Chiralpak® IC column (4.6 mm × 250 mm) at 30 °C and 50:50 hexanes:iPrOH (1.3 mL min^{-1}) as mobile phase, λ = 225 nm (t_r = 20.5 min (major), 25.1 min (minor)).

^{1}H NMR (500 MHz, CDCl$_3$) δ 7.90 (d, *J* = 8.2 Hz, 2H), 7.41 (d, *J* = 8.3 Hz, 2H), 7.33 (d, *J* = 8.1 Hz, 2H), 7.00 (d, *J* = 8.3 Hz, 2H), 4.30 (dd, *J* = 9.8, 8.0 Hz, 1H), 3.74 (dd, *J* = 9.9, 7.7 Hz, 1H), 3.55 (quint, *J* = 8.1 Hz, 1H), 2.83 (dd, *J* = 17.3, 8.4 Hz, 1H), 2.54 (dd, *J* = 17.3, 8.9 Hz, 1H), 2.45 (s, 3H).

^{13}C NMR (126 MHz, CDCl$_3$) δ 171.8, 145.5, 139.1, 135.0, 132.2, 129.8, 128.4, 128.1, 121.6, 53.5, 39.4, 36.7, 21.8.

$[\alpha]^{20}_D$ = -22 (*c* 0.91, CHCl$_3$, 87:13 er)

HRMS (ESI+) *m*/*z* calculated for $C_{17}H_{16}BrNO_3S+H^+$ $[M+H]^+$ 394.01070, found 394.01033.

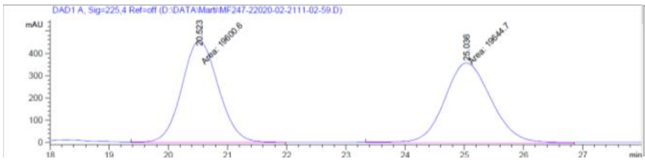

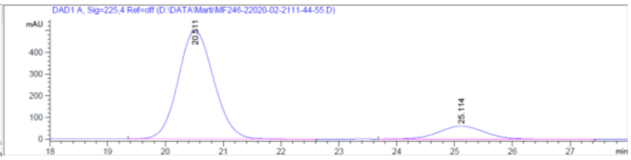

Signal 1: DAD1 A, Sig=225,4 Ref=off

Peak #	RetTime [min]	Type	Width [min]	Area [mAU*s]	Height [mAU]	Area %
1	20.523	MM	0.7155	1.96006e4	456.59232	49.9438
2	25.036	MM	0.9153	1.96447e4	357.70822	50.0562
Totals :				3.92453e4	814.30054	

Signal 1: DAD1 A, Sig=225,4 Ref=off

Peak #	RetTime [min]	Type	Width [min]	Area [mAU*s]	Height [mAU]	Area %
1	20.511	BB	0.6635	2.12924e4	499.33578	86.8995
2	25.114	BB	0.8367	3209.93311	59.56511	13.1005
Totals :				2.45023e4	558.90089	

(*R*)-1-Tosyl-4-(4-iodophenyl)pyrrolidin-2-one (4bi)

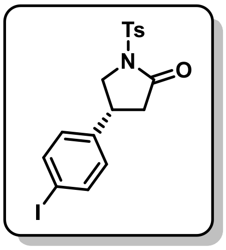

Following General Procedure (i). Clear-orange solid.

Run 1: (93.6 mg, 0.212 mmol, 70% yield). **Run 2:** (91.8 mg, 0.208 mmol, 69% yield). **Average: 70% yield.**

The enantiomeric ratio was determined by HPLC analysis in comparison to a racemic material using Daicel Chiralpak® IC column (4.6 mm × 250 mm) at 30 °C and 50:50 hexanes:iPrOH (1.3 mL min^{-1}) as mobile phase, λ = 230 nm (t_r = 19.7 min (major), 23.2 min (minor)).

^{1}H NMR (400 MHz, CDCl$_3$) δ 7.90 (d, *J* = 8.3 Hz, 2H), 7.61 (d, *J* = 8.4 Hz, 2H), 7.33 (d, *J* = 8.2 Hz, 2H), 6.87 (d, *J* = 8.3 Hz, 2H), 4.29 (dd, *J* = 10.0, 7.8 Hz, 1H), 3.73 (dd, *J* = 10.0, 7.6 Hz, 1H), 3.54 (quint, *J* = 8.1 Hz, 1H), 2.82 (dd, *J* = 17.3, 8.4 Hz, 1H), 2.54 (dd, *J* = 17.3, 8.8 Hz, 1H), 2.45 (s, 3H).

^{13}C NMR (101 MHz, CDCl$_3$) δ 171.8, 145.5, 139.7, 138.1, 135.0, 129.8, 128.6, 128.1, 92.9, 53.5, 39.3, 36.8, 21.8.

$[\alpha]^{20}_{D}$ = -19 (*c* 0.98, CHCl$_3$, 85:15 er)

HRMS (ESI+) *m/z* calculated for $C_{17}H_{16}INO_3S+H^+$ $[M+H]^+$ 441.99683, found 441.99660.

HRMS (ESI+) *m/z* calculated for $C_{17}H_{16}INO_3S+Na^+$ $[M+Na]^+$ 463.97878, found 463.97840.

mp: 121°C.

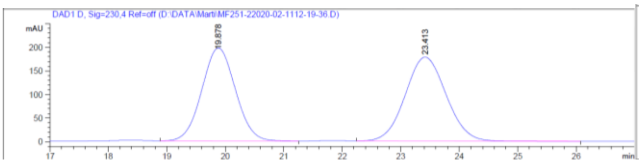

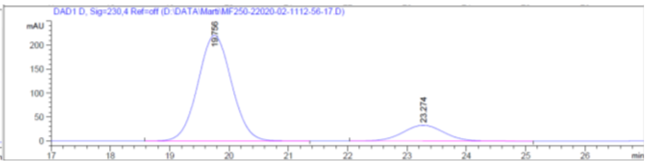

Signal 3: DAD1 D, Sig=230,4 Ref=off

Peak #	RetTime [min]	Type	Width [min]	Area [mAU*s]	Height [mAU]	Area %
1	19.878	BB	0.6201	7867.69238	196.93800	47.5977
2	23.413	BB	0.7562	8661.86621	177.84183	52.4023
Totals :				1.65296e4	374.77983	

Signal 3: DAD1 D, Sig=230,4 Ref=off

Peak #	RetTime [min]	Type	Width [min]	Area [mAU*s]	Height [mAU]	Area %
1	19.756	BB	0.6138	8670.30371	219.97101	84.6656
2	23.274	BB	0.7358	1570.33887	32.84156	15.3344
Totals :				1.02406e4	252.81257	

(*R*)-4-(*p*-Tolyl)-1-tosylpyrrolidin-2-one (4bj)

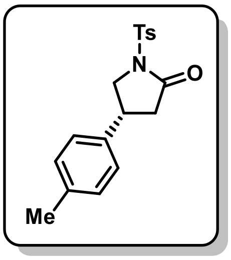

Following General Procedure (i). Colorless solid.

Run 1: (78.6 mg, 0.238 mmol, 79% yield). **Run 2:** (77.8 mg, 0.236 mmol, 79% yield). **Average: 79% yield.**

The enantiomeric ratio was determined by HPLC analysis in comparison to a racemic material using Daicel Chiralpak® IC column (4.6 mm × 250 mm) at 30 °C and 50:50 hexanes:iPrOH (1.3 mL min^{-1}) as mobile phase, λ = 230 nm (t_r = 15.4 min (major), 21.2 min (minor)).

^{1}H NMR (400 MHz, CDCl$_3$) δ 7.93 (d, *J* = 8.4 Hz, 2H), 7.34 (d, *J* = 8.1 Hz, 2H), 7.12 (d, *J* = 7.9 Hz, 2H), 7.02 (d, *J* = 8.1 Hz, 2H), 4.30 (dd, *J* = 9.9, 7.9 Hz, 1H), 3.76 (dd, *J* = 9.9, 8.0 Hz, 1H), 3.55 (quint, *J* = 8.0 Hz, 1H), 2.81 (dd, *J* = 17.3, 8.4 Hz, 1H), 2.58 (dd, *J* = 17.3, 9.4 Hz, 1H), 2.45 (s, 3H), 2.32 (s, 3H).

^{13}C NMR (101 MHz, CDCl$_3$) δ 172.3, 145.3, 137.4, 136.9, 135.2, 129.8, 129.7, 128.2, 126.5, 53.9, 39.7, 36.9, 21.8, 21.1.

$[\alpha]^{20}_{D}$ = -19 (*c* 1.07, CHCl$_3$, 86:14 er)

HRMS (ESI+) *m/z* calculated for $C_{18}H_{19}NO_3S+H^+$ $[M+H]^+$ 330.11584, found 330.11540.

HRMS (ESI+) *m/z* calculated for $C_{18}H_{19}NO_3S+Na^+$ $[M+Na]^+$ 352.09779, found 352.09710.

mp: 93-95°C.

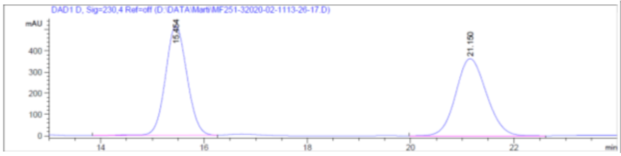

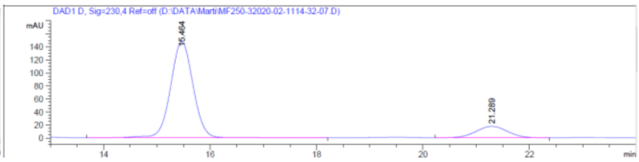

Signal 3: DAD1 D, Sig=230,4 Ref=off

Peak #	RetTime [min]	Type	Width [min]	Area [mAU*s]	Height [mAU]	Area %
1	15.454	BB	0.4496	1.48681e4	513.81287	49.9642
2	21.150	BB	0.6373	1.48894e4	362.45013	50.0358
Totals :				2.97575e4	876.26300	

Signal 3: DAD1 D, Sig=230,4 Ref=off

Peak #	RetTime [min]	Type	Width [min]	Area [mAU*s]	Height [mAU]	Area %
1	15.464	BB	0.4571	4342.44385	147.65497	85.7960
2	21.289	BB	0.6324	718.91461	17.45893	14.2040
Totals :				5061.35846	165.11390	

(*R*)-1-Tosyl-4-(4-nitrophenyl)pyrrolidin-2-one (4bk)

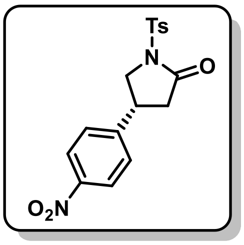

Following General Procedure (i). Yellow solid.

Run 1: (80.4 mg, 0.223 mmol, 74% yield). **Run 2:** (73.0 mg, 0.202 mmol, 67% yield). **Average: 70% yield.**

The enantiomeric ratio was determined by SFC analysis in comparison to a racemic material using Daicel Chiralpak® OJ-3 column (4.6 mm × 250 mm) at 30 °C and 10% MeOH in supercritical CO_2 (3.0 mL min^{-1}) as mobile phase, λ = 225 nm (t_r = 11.5 min (minor), 12.2 min (major)).

^{1}H NMR (400 MHz, $CDCl_3$) δ 8.17 (d, *J* = 8.7 Hz, 2H), 7.93 (d, *J* = 8.3 Hz, 2H), 7.34 (m, 4H), 4.37 (dd, *J* = 10.0, 7.7 Hz, 1H), 3.82 (dd, *J* = 10.0, 7.2 Hz, 1H), 3.72 (quint, *J* = 8.0, 1H), 2.92 (dd, *J* = 17.3, 8.4 Hz, 1H), 2.61 (dd, *J* = 17.3, 8.4 Hz, 1H), 2.46 (s, 3H).

^{13}C NMR (101 MHz, $CDCl_3$) δ 171.2, 147.5, 145.8, 134.9, 129.9, 128.3, 127.7, 124.4, 53.1, 39.3, 37.0, 21.8.

$[\alpha]^{20}_D$ = -22 (*c* 1.05, $CHCl_3$, 84:16 er)

HRMS (ESI+) *m/z* calculated for $C_{17}H_{16}N_2O_5S+Na^+$ $[M+Na]^+$ 383.06721, found 383.06609.

mp: 157°C.

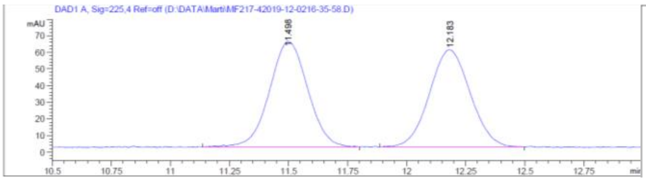

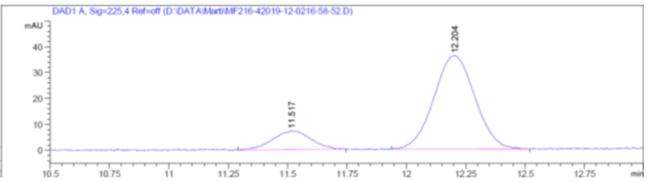

Signal 1: DAD1 A, Sig=225,4 Ref=off

Peak #	RetTime [min]	Type	Width [min]	Area [mAU*s]	Height [mAU]	Area %
1	11.498	VB R	0.1570	711.54370	63.35781	50.3119
2	12.183	VB R	0.1590	702.72174	58.35484	49.6881
Totals :				1414.26544	121.71265	

Signal 1: DAD1 A, Sig=225,4 Ref=off

Peak #	RetTime [min]	Type	Width [min]	Area [mAU*s]	Height [mAU]	Area %
1	11.517	BV R	0.1336	81.22764	7.25734	15.7541
2	12.204	BB	0.1434	434.37021	36.21155	84.2459
Totals :				515.59785	43.46889	

(*R*)-4-(2-Phenoxyphenyl)-1-tosylpyrrolidin-2-one (4bl)

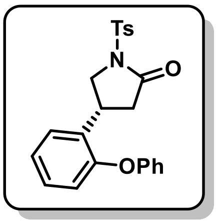

Following General Procedure (i). Colorless oil.

Run 1: (104.0 mg, 0.255 mmol, 85% yield). **Run 2:** (104.5 mg, 0.256 mmol, 67% yield). **Average: 85% yield.**

The enantiomeric ratio was determined by HPLC analysis in comparison to a racemic material using Daicel Chiralpak® IC column (4.6 mm × 250 mm) at 30 °C and 50:50 hexanes:iPrOH (1.3 mL min^{-1}) as mobile phase, λ = 210 nm (t_r = 23.5 min (minor), 41.9 min (major)).

^{1}H NMR (400 MHz, $CDCl_3$) δ 7.79 (d, *J* = 8.3 Hz, 2H), 7.30 – 7.21 (m, 2H), 7.21 – 7.17 (m, 2H), 7.16 – 7.02 (m, 3H), 6.97 (m, 1H), 6.83 – 6.74 (m, 3H), 4.22 (dd, *J* = 9.4, 7.8 Hz, 1H), 3.88 – 3.73 (m, 2H), 2.76 – 2.59 (m, 2H), 2.34 (s, 3H).

^{13}C NMR (101 MHz, $CDCl_3$) δ 172.5, 156.6, 155.0, 145.2, 135.2, 130.7, 130.1, 129.7, 128.9, 128.1, 127.8, 123.9, 123.8, 118.9, 118.6, 52.6, 38.4, 32.2, 21.8.

$[\alpha]^{20}_{D}$ = -5 (*c* 1.00, $CHCl_3$, 57:43 er)

HRMS (ESI+) *m/z* calculated for $C_{23}H_{21}NO_4S+H^+$ $[M+H]^+$ 408.12641, found 408.12610.

HRMS (ESI+) *m/z* calculated for $C_{23}H_{21}NO_4S+Na^+$ $[M+Na]^+$ 430.10835, found 430.10810.

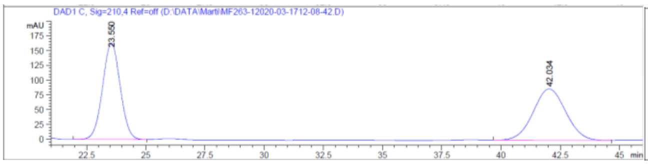

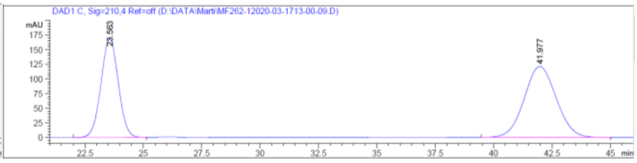

Signal 2: DAD1 C, Sig=210,4 Ref=off

Peak #	RetTime [min]	Type	Width [min]	Area [mAU*s]	Height [mAU]	Area %
1	23.550	BB	0.8052	8423.46582	162.91248	50.3651
2	42.034	BB	1.4585	8301.33594	86.89524	49.6349
Totals :				1.67248e4	249.80772	

Signal 2: DAD1 C, Sig=210,4 Ref=off

Peak #	RetTime [min]	Type	Width [min]	Area [mAU*s]	Height [mAU]	Area %
1	23.563	BB	0.8051	8699.12402	168.84608	42.9603
2	41.977	BB	1.4506	1.15501e4	121.11877	57.0397
Totals :				2.02492e4	289.96486	

(*R*)-1-Tosyl-4-(2-methoxyphenyl)pyrrolidin-2-one (4bm)

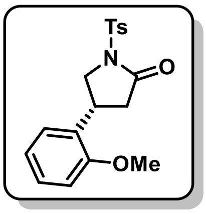

Following General Procedure (i). White solid.

Run 1: (83.3 mg, 0.240 mmol, 80% yield). **Run 2:** (88.9 mg, 0.257 mmol, 85% yield). **Average: 82% yield.**

The enantiomeric ratio was determined by HPLC analysis in comparison to a racemic material using Daicel Chiralpak® IB column (4.6 mm × 250 mm) at 30 °C and 80:20 hexanes:iPrOH (1.0 mL min^{-1}) as mobile phase, λ = 225 nm (t_r = 13.5 min (major), 15.2 min (minor)).

^{1}H NMR (500 MHz, $CDCl_3$) δ 7.93 (d, *J* = 8.3 Hz, 2H), 7.33 (d, *J* = 8.1 Hz, 2H), 7.27 – 7.21 (m, 1H), 7.02 (d, *J* = 6.8 Hz, 1H), 6.88 – 6.85 (m, 2H), 4.29 (dd, *J* = 9.5, 8.4 Hz, 1H), 3.82 (dd, *J* = 9.7, 7.0 Hz, 1H), 3.75 (m, 1H), 3.67 (s, 3H), 2.77 (dd, *J* = 17.5, 9.1 Hz, 1H), 2.66 (dd, *J* = 17.5, 8.0 Hz, 1H), 2.44 (s, 3H).

^{13}C NMR (126 MHz, $CDCl_3$) δ 173.0, 157.2, 145.1, 135.4, 129.7, 128.8, 128.3, 128.2, 127.7, 120.7, 110.8, 55.1, 52.5, 37.9, 32.7, 21.7.

$[\alpha]^{20}_{D}$ = -14 (*c* 1.02, $CHCl_3$, 93:7 er)

HRMS (ESI+) *m/z* calculated for $C_{18}H_{19}NO_4S+H^+$ $[M+H]^+$ 346,11076, found 346,11042.

HRMS (ESI+) *m/z* calculated for $C_{18}H_{19}NO_4S+Na^+$ $[M+Na]^+$ 368,09270 found 368,09223.

mp: 140-143°C.

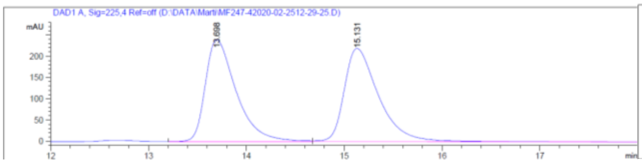

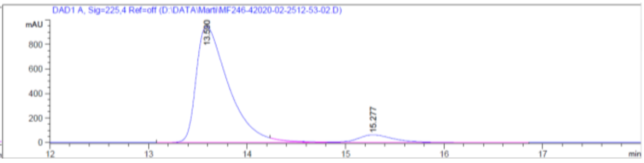

Signal 1: DAD1 A, Sig=225,4 Ref=off

Peak #	RetTime [min]	Type	Width [min]	Area [mAU*s]	Height [mAU]	Area %
1	13.698	BV	0.3297	5241.12891	239.17599	49.3111
2	15.131	VBA	0.3690	5387.57666	219.00600	50.6889
Totals :				1.06287e4	458.18199	

Signal 1: DAD1 A, Sig=225,4 Ref=off

Peak #	RetTime [min]	Type	Width [min]	Area [mAU*s]	Height [mAU]	Area %
1	13.590	BV R	0.3410	2.15243e4	947.54419	92.7897
2	15.277	VB E	0.3972	1672.55273	62.30276	7.2103
Totals :				2.31969e4	1009.84695	

(*R*)-1-Tosyl-4-(2-nitrophenyl)pyrrolidin-2-one (4bn)

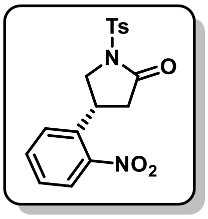

Following General Procedure (i). Yellow oil.

Run 1: (72.0 mg, 0.195 mmol, 65% yield). **Run 2:** (74.1 mg, 0.205 mmol, 68% yield). **Average: 66% yield.**

The enantiomeric ratio was determined by HPLC analysis in comparison to a racemic material using Daicel Chiralpak® IA column (4.6 mm × 250 mm) at 30 °C and 70:30 hexanes:iPrOH (1.3 mL min^{-1}) as mobile phase, λ = 225 nm (t_r = 9.9 min (minor), 11.8 min (major)).

^{1}H NMR (500 MHz, CDCl$_3$) δ 7.93 – 7.88 (m, 3H), 7.55 (m, 1H), 7.44 (m, 1H), 7.36 - 7.31 (m, 3H), 4.31 (dd, *J* = 10.4, 7.7 Hz, 1H), 4.05 (m, 1H), 3.98 (dd, *J* = 10.4, 5.0 Hz, 1H), 2.94 (dd, *J* = 17.8, 8.9 Hz, 1H), 2.63 (dd, *J* = 17.8, 5.9 Hz, 1H), 2.45 (s, 3H).

^{13}C NMR (126 MHz, CDCl$_3$) δ 171.7, 149.4, 145.6, 135.6, 134.9, 133.9, 129.9, 128.7, 128.2, 127.4, 125.2, 53.4, 39.3, 32.1, 21.8.

[α]$^{20}_{D}$ = +6 (*c* 1.02, CHCl$_3$, 91:9 er)

HRMS (ESI+) *m/z* calculated for $C_{17}H_{16}N_2O_5S+H^+$ [M+H]$^+$ 361.08527, found 361.08480.

HRMS (ESI+) *m/z* calculated for $C_{17}H_{16}N_2O_5S+Na^+$ [M+Na]$^+$ 383.06721, found 383.06670.

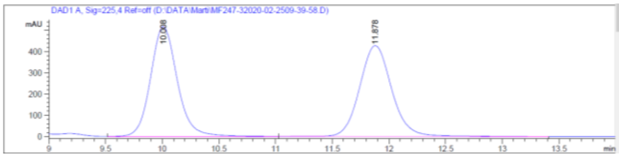

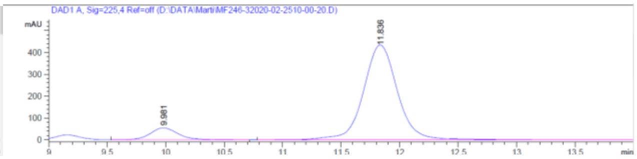

Signal 1: DAD1 A, Sig=225,4 Ref=off

Peak #	RetTime [min]	Type	Width [min]	Area [mAU*s]	Height [mAU]	Area %
1	10.008	BB	0.2506	8443.06543	516.84204	50.0662
2	11.878	BB	0.3023	8420.74414	426.52744	49.9338
Totals :				1.68638e4	943.36948	

Signal 1: DAD1 A, Sig=225,4 Ref=off

Peak #	RetTime [min]	Type	Width [min]	Area [mAU*s]	Height [mAU]	Area %
1	9.981	BB	0.2481	879.83301	54.00305	9.0797
2	11.836	BBA	0.3089	8810.26465	433.86591	90.9203
Totals :				9690.09766	487.86896	

(*R*)-1-Tosyl-4-(2-bromophenyl)pyrrolidin-2-one (4bo)

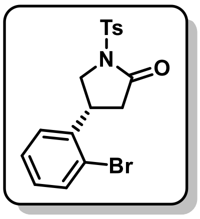

Following General Procedure (i). Orange gum.

Run 1: (82.7 mg, 0.210 mmol, 70% yield, 66:34 er). **Run 2:** (77.7 mg, 0.197 mmol, 65% yield). **Average: 67% yield.**

The enantiomeric ratio was determined by HPLC analysis in comparison to a racemic material using Daicel Chiralpak® IB column (4.6 mm × 250 mm) at 30 °C and 90:10 hexanes:iPrOH (1.0 mL min^{-1}) as mobile phase, λ = 225 nm (t_r = 24.5 min (major), 26.3 min (minor)).

^{1}H NMR (400 MHz, CDCl$_3$) δ 7.90 (d, *J* = 8.4 Hz, 2H), 7.57 (dd, *J* = 7.9, 1.3 Hz, 1H), 7.32 (d, *J* = 8.1 Hz, 2H), 7.22 (td, *J* = 7.5, 1.2 Hz, 1H), 7.16 – 7.07 (m, 2H), 4.30 (dd, *J* = 10.1, 7.6 Hz, 1H), 4.00 (quint, *J* = 8.0 Hz, 1H), 3.85 (dd, *J* = 10.1, 5.8 Hz, 1H), 2.88 (dd, *J* = 17.5, 8.7 Hz, 1H), 2.59 (dd, *J* = 17.5, 6.9 Hz, 1H), 2.44 (s, 3H).
^{13}C NMR (101 MHz, CDCl$_3$) δ 172.0, 145.4, 139.3, 135.1, 133.6, 129.8, 129.2, 128.2, 126.9, 124.4, 52.7, 38.6, 36.3, 21.8.
$[\alpha]^{20}_{D}$ = -23 (*c* 1.00, CHCl$_3$, 66:34 er)
HRMS (ESI+) *m/z* calculated for $C_{17}H_{16}BrO_3S+H^+$ $[M+H]^+$ 394.01070, found 394.01050.

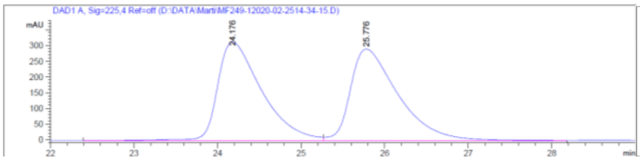

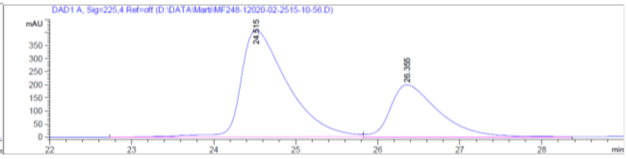

Signal 1: DAD1 A, Sig=225,4 Ref=off

Peak #	RetTime [min]	Type	Width [min]	Area [mAU*s]	Height [mAU]	Area %
1	24.176	BV	0.5475	1.14702e4	313.03329	49.9514
2	25.776	VB	0.5917	1.14925e4	290.41287	50.0486
Totals :				2.29627e4	603.44617	

Signal 1: DAD1 A, Sig=225,4 Ref=off

Peak #	RetTime [min]	Type	Width [min]	Area [mAU*s]	Height [mAU]	Area %
1	24.515	BV	0.5815	1.59856e4	407.66376	66.6621
2	26.355	VB	0.6026	7994.42480	198.20967	33.3379
Totals :				2.39800e4	605.87343	

(*R*)-1-Tosyl-4-(3-chlorophenyl)pyrrolidin-2-one (4bp)

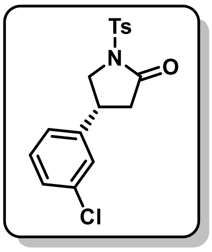

Following General Procedure (i). Yellowish solid.
Run 1: (74.3 mg, 0.212 mmol, 71% yield). **Run 2:** (72.5 mg, 0.207 mmol, 69% yield). **Average: 70% yield.**
The enantiomeric ratio was determined by HPLC analysis in comparison to a racemic material using Daicel Chiralpak® IC column (4.6 mm × 250 mm) at 30 °C and 50:50 hexanes:iPrOH (1.3 mL min^{-1}) as mobile phase, λ = 225 nm (t_r = 27.5 min (major), 34.1 min (minor)).
^{1}H NMR (400 MHz, CDCl$_3$) δ 7.92 (d, *J* = 8.4 Hz, 2H), 7.34 (d, *J* = 8.0 Hz, 2H), 7.26 – 7.22 (m, 2H), 7.08 (m, 1H), 7.02 (m, 1H), 4.32 (dd, *J* = 10.0, 7.8 Hz, 1H), 3.76 (dd, *J* = 10.0, 7.5 Hz, 1H), 3.57 (m, 1H), 2.84 (dd, *J* = 17.3, 8.5 Hz, 1H), 2.56 (dd, *J* = 17.3, 8.8 Hz, 1H), 2.45 (s, 3H).
^{13}C NMR (101 MHz, CDCl$_3$) δ 171.7, 145.5, 142.2, 135.0, 135.0, 130.5, 129.9, 128.2, 127.9, 126.9, 124.9, 53.4, 39.4, 36.9, 21.8.
$[\alpha]^{20}_{D}$ = -16 (*c* 1.10, CHCl$_3$, 86:14 er)
HRMS (ESI+) *m/z* calculated for $C_{17}H_{16}ClNO_3S+H^+$ $[M+H]^+$ 350.06122, found 350,06101.
mp: 127-128°C.

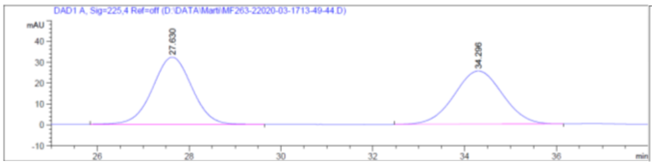

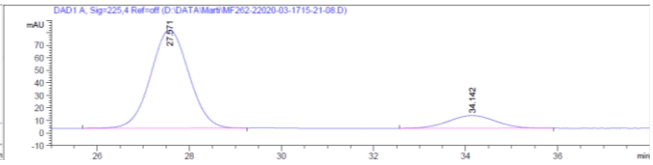

Signal 1: DAD1 A, Sig=225,4 Ref=off

Peak #	RetTime [min]	Type	Width [min]	Area [mAU*s]	Height [mAU]	Area %
1	27.630	BB	0.9045	1906.30286	32.11412	50.4880
2	34.296	BB	1.0893	1869.45386	25.44028	49.5120
Totals :				3775.75671	57.55440	

Signal 1: DAD1 A, Sig=225,4 Ref=off

Peak #	RetTime [min]	Type	Width [min]	Area [mAU*s]	Height [mAU]	Area %
1	27.571	BB	0.8910	4492.52539	78.11236	86.2477
2	34.142	BB	0.8571	716.33740	10.03198	13.7523
Totals :				5208.86279	88.14433	

(*R*)-4-(3-(Cyclopentyloxy)-4-methoxyphenyl)-1-tosylpyrrolidin-2-one (4bq)

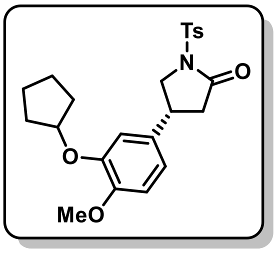

Following General Procedure (i). Clear-orange gum.

Run 1: (96.2 mg, 0.224 mmol, 75% yield). **Run 2:** (93.7 mg, 0.218 mmol, 73% yield). **Average: 74% yield**.

The enantiomeric ratio was determined by HPLC analysis in comparison to a racemic material using Daicel Chiralpak® IC column (4.6 mm × 250 mm) at 30 °C and 50:50 hexanes:iPrOH (1.3 mL min^{-1}) as mobile phase, λ = 210 nm (t_r = 16.1 min (major), 29.3 min (minor)).

^{1}H NMR (400 MHz, CDCl$_3$) δ 7.92 (d, *J* = 8.3 Hz, 2H), 7.33 (d, *J* = 8.1 Hz, 2H), 6.77 (m, 1H), 6.67 – 6.62 (m, 2H), 4.68 (m, 1H), 4.28 (dd, *J* = 9.9, 7.9 Hz, 1H), 3.81 (s, 3H), 3.75 (dd, *J* = 9.9, 7.7 Hz, 1H), 3.51 (quint, *J* = 8.2 Hz, 1H), 2.80 (dd, *J* = 17.3, 8.4 Hz, 1H), 2.57 (dd, *J* = 17.3, 9.0 Hz, 1H), 2.44 (s, 3H), 1.99 – 1.59 (m, 8H).

^{13}C NMR (101 MHz, CDCl$_3$) δ 172.3, 149.6, 148.1, 145.3, 135.2, 132.4, 129.8, 128.2, 118.6, 113.5, 112.3, 80.7, 56.2, 54.0, 39.8, 36.8, 32.9, 24.1, 21.8.

$[\alpha]^{20}_D$ = -24 (*c* 1.08, CHCl$_3$, 83:17 er)

HRMS (ESI+) *m*/*z* calculated for $C_{23}H_{27}NO_5S+H^+$ $[M+H]^+$ 430.16827, found 430.16810.

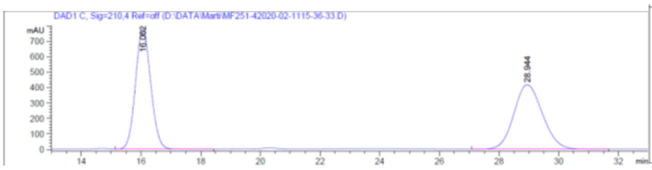

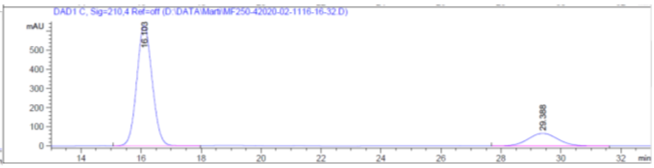

Signal 2: DAD1 C, Sig=210,4 Ref=off

Peak #	RetTime [min]	Type	Width [min]	Area [mAU*s]	Height [mAU]	Area %
1	16.062	BB	0.5528	2.76054e4	776.44836	49.8664
2	28.944	BB	1.0319	2.77532e4	417.29385	50.1336
Totals :				5.53586e4	1193.74222	

Signal 2: DAD1 C, Sig=210,4 Ref=off

Peak #	RetTime [min]	Type	Width [min]	Area [mAU*s]	Height [mAU]	Area %
1	16.103	BB	0.5531	2.23594e4	628.41785	83.2518
2	29.388	BB	1.0419	4498.17578	65.93841	16.7482
Totals :				2.68576e4	694.35625	

(*R*)-4-(4-Methoxyphenyl)-1-((2-nitrophenyl)sulfonyl)pyrrolidin-2-one (4ca).

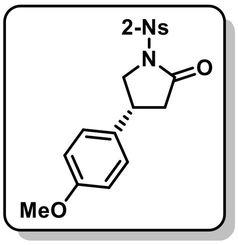

Following General Procedure (ii). Yellow oil.

Run 1: (77.3 mg, 0.205 mmol, 69% yield). **Run 2:** (73.0 mg, 0.194 mmol, 65% yield). **Average: 67% yield**.

The enantiomeric ratio was determined by SFC analysis in comparison to a racemic material using Daicel Chiralpak® IC column (4.6 mm × 250 mm) at 30 °C and 7% MeOH in supercritical CO_2 (3.0 mL min^{-1}) as mobile phase, λ = 225 nm (t_r = 14.6 min (minor), 15.5 min (major)).

^{1}H NMR (500 MHz, $CDCl_3$) δ 8.53 – 7.45 (m, 1H), 7.87 – 7.73 (m, 3H), 7.20 (d, *J* = 8.6 Hz, 2H), 6.90 (d, *J* = 8.7 Hz, 2H), 4.41 (dd, *J* = 10.0, 8.2 Hz, 1H), 3.96 (t, *J* = 9.6 Hz, 1H), 3.81 (s, 3H), 3.78 – 3.64 (m, 1H), 2.82 (dd, *J* = 17.3, 8.3 Hz, 1H), 2.73 (dd, *J* = 17.3, 10.9 Hz, 1H).

13C NMR (126 MHz, $CDCl_3$) δ 172.53, 159.10, 148.03, 135.04, 134.56, 132.08, 131.52, 130.97, 127.91, 124.31, 114.45, 55.35, 53.78, 39.63, 37.43.

$[\alpha]^{20}_D$ = +19 (*c* 0.50, MeOH, 70:30 er).

HRMS (ESI+) *m/z* calculated for $C_{17}H_{16}N_2O_6S+H^+$ $[M+H]^+$ 377.08018, found 377.07969.

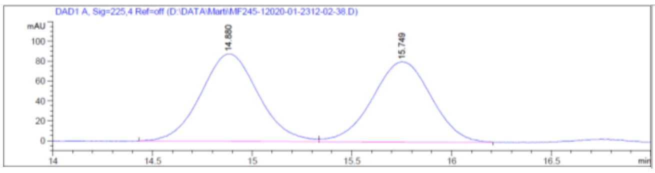

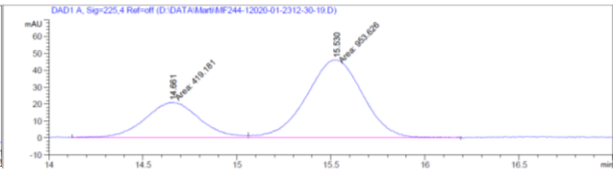

Signal 1: DAD1 A, Sig=225,4 Ref=off

Peak #	RetTime [min]	Type	Width [min]	Area [mAU*s]	Height [mAU]	Area %
1	14.880	BV R	0.2394	1764.88013	87.84776	51.5192
2	15.749	VV R	0.2531	1660.79224	80.54211	48.4808
Totals :				3425.67236	168.38988	

Signal 1: DAD1 A, Sig=225,4 Ref=off

Peak #	RetTime [min]	Type	Width [min]	Area [mAU*s]	Height [mAU]	Area %
1	14.661	MF	0.3347	419.18085	20.87205	30.5346
2	15.530	FM	0.3457	953.62640	45.97793	69.4654
Totals :				1372.80725	66.84998	

4-Phenyl-1-((2-nitrophenyl)sulfonyl)pyrrolidin-2-one (4cb).

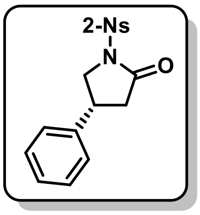

Following General Procedure (ii). Colorless oil.

Run 1: (77.0 mg, 0.222 mmol, 74% yield). **Run 2:** (72.8 mg, 0.211 mmol, 70% yield). **Average: 72% yield**.

The enantiomeric ratio was determined by SFC analysis in comparison to a racemic material using Daicel Chiralpak® IC column (4.6 mm × 250 mm) at 30 °C and 4% MeOH in supercritical CO_2 (3.0 mL min^{-1}) as mobile phase, λ = 210 nm (t_r = 15.9 min (major), 16.7 min (minor)).

^{1}H NMR (500 MHz, $CDCl_3$) δ 8.61-8.34 (m, 1H), 7.89 - 7.60 (m, 3H), 7.39 – 7.25 (m, 5H), 4.45 (dd, *J* = 10.0, 8.2 Hz, 1H), 4.04 (t, *J* = 9.5 Hz, 1H), 3.77 (quint, *J* = 8.5 Hz, 1H), 2.85 (dd, *J* = 17.3, 8.4 Hz, 1H), 2.77 (dd, *J* = 17.3, 10.8 Hz, 1H).

^{13}C NMR (126 MHz, $CDCl_3$) δ 172.46, 148.05, 139.13, 135.15, 134.55, 132.13, 131.46, 129.13, 127.82, 126.90, 124.37, 53.58, 39.42, 38.05.

$[\alpha]^{20}_D$ = +20 (*c* 0.50, MeOH, 70:30 er)

HRMS (ESI+) *m/z* calculated for $C_{16}H_{14}N_2O_5S+H^+$ $[M+H]^+$ 347.06962, found 347.06950.

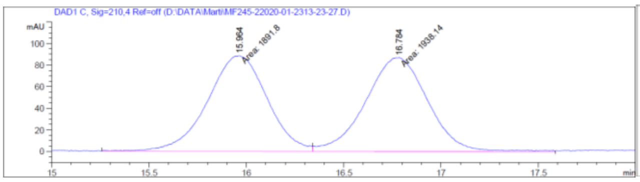

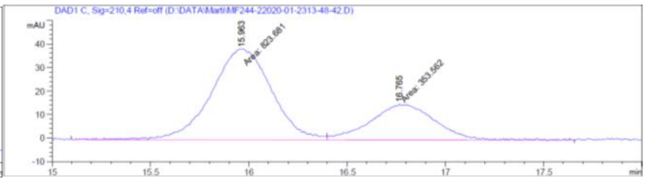

Signal 2: DAD1 C, Sig=210,4 Ref=off

Peak #	RetTime [min]	Type	Width [min]	Area [mAU*s]	Height [mAU]	Area %
1	15.964	MF	0.3558	1891.79810	88.62054	49.3950
2	16.784	FM	0.3716	1938.14185	86.93184	50.6050
Totals :				3829.93994	175.55238	

Signal 2: DAD1 C, Sig=210,4 Ref=off

Peak #	RetTime [min]	Type	Width [min]	Area [mAU*s]	Height [mAU]	Area %
1	15.963	MF	0.3544	823.68066	38.73061	69.9669
2	16.765	FM	0.3866	353.56189	15.24101	30.0331
Totals :				1177.24255	53.97161	

(*R*)-1-((2-Nitrophenyl)sulfonyl)-4-(4-(trifluoromethyl)phenyl)pyrrolidin-2-one (4cc).

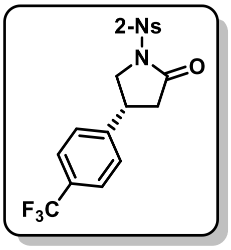

Following General Procedure (ii). Yellow oil.

Run 1: (54.7 mg, 0.158 mmol, 53% yield). **Run 2:** (44,3 mg, 0.127 mmol, 43% yield). **Average: 48% yield**.

The enantiomeric ratio was determined by SFC analysis in comparison to a racemic material using Daicel Chiralpak® IC column (4.6 mm × 250 mm) at 30 °C and 4% MeOH in supercritical CO_2 (3.0 mL min^{-1}) as mobile, λ = 210 nm (t_r = 24.2 min (minor), 25.8 min (major)).

^{1}H NMR (500 MHz, $CDCl_3$) δ 8.52 - 8.42 (m, 1H), 7.88 – 7.74 (m, 3H), 7.64 (d, *J* = 8.2 Hz, 2H), 7.42 (d, *J* = 8.2 Hz, 2H), 4.48 (dd, *J* = 10.1, 8.2 Hz, 1H), 4.03 (dd, *J* = 10.0, 9.0 Hz, 1H), 3.90 – 3.79 (quint, *J* = 6.4 Hz, 1H), 2.90 (dd, *J* = 17.3, 8.4 Hz, 1H), 2.77 (dd, *J* = 17.3, 10.4 Hz, 1H).

^{13}C NMR (126 MHz, $CDCl_3$) δ 171.84, 148.03, 143.30, 135.27, 134.58, 132.19, 131.31, 130.52, 130.27, 130.00, 129.75, 127.37, 127.17, 126.14, 126.11, 126.08, 126.06, 125.00, 124.41, 122.84, 120.67, 53.12, 39.17, 37.71.

^{19}F NMR (470 MHz, $CDCl_3$) δ – 62.61 ppm.

$[\alpha]^{20}_D$ = +8 (*c* 0.95, MeOH, 65:35 er).

HRMS (ESI+) *m*/*z* calculated for $C_{17}H_{13}F_3N_2O_5S+H^+$ $[M+H]^+$ 415.05700, found 415.05689.

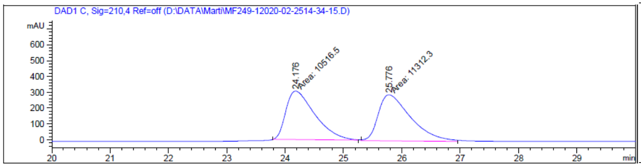

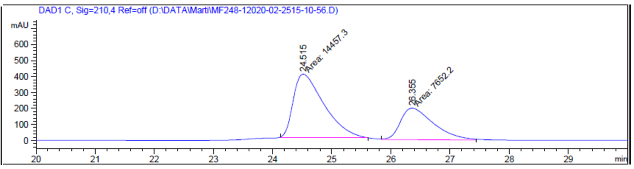

Signal 2: DAD1 C, Sig=210,4 Ref=off

Peak #	RetTime [min]	Type	Width [min]	Area [mAU*s]	Height [mAU]	Area %
1	3.124	BB	0.1526	113.91563	11.80131	0.5184
2	3.665	BB	0.1046	10.57407	1.45828	0.0481
3	7.198	BB	0.1717	21.86050	1.85268	0.0995
4	24.176	MM	0.5734	1.05165e4	305.65854	47.8563
5	25.776	MM	0.6472	1.13123e4	291.31415	51.4778
Totals :				2.19751e4	612.08495	

Signal 2: DAD1 C, Sig=210,4 Ref=off

Peak #	RetTime [min]	Type	Width [min]	Area [mAU*s]	Height [mAU]	Area %
1	24.515	MM	0.6048	1.44573e4	398.41202	65.3895
2	26.355	MM	0.6486	7652.20361	196.63274	34.6105
Totals :				2.21095e4	595.04475	

(*R*)-4-(4-Methoxyphenyl)-1-((4-nitrophenyl)sulfonyl)pyrrolidin-2-one (4da)

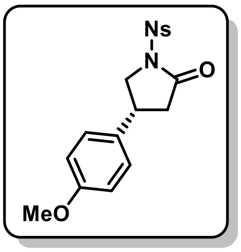

Following General Procedure (ii). Yellow oil.

Run 1: (96.5 mg, 0.257 mmol, 86% yield). **Run 2:** (92.9 mg, 0.247 mmol, 82% yield). **Average: 84% yield**.

The enantiomeric ratio was determined by HPLC analysis in comparison to a racemic material using Daicel Chiralpak® IB column (4.6 mm × 250 mm) at 30 °C and 50:50 hexanes:iPrOH (1.3 mL min^{-1}) as mobile phase, λ = 260 nm (t_r = 15.0 min (major), 17.5 min (minor)).

^{1}H NMR (500 MHz, $CDCl_3$) δ 8.39 (d, *J* = 8.9 Hz, 2H), 8.24 (d, *J* = 8.9 Hz, 2H), 7.06 (d, *J* = 8.6 Hz, 2H), 6.85 (d, *J* = 8.7 Hz, 2H), 4.34 (dd, *J* = 10.0, 7.8 Hz, 1H), 3.79 (s, 3H), 3.78 (m, 1H), 3.60 (m, 1H), 2.85 (dd, *J* = 17.5, 8.3 Hz, 1H), 2.63 (dd, *J* = 17.5, 9.2 Hz, 1H).

^{13}C NMR (126 MHz, CDCl$_3$) δ 172.40, 159.19, 150.94, 143.37, 131.10, 129.60, 127.60, 124.28, 114.50, 88.22, 55.36, 54.19, 39.39, 36.79.

[α]$^{20}_{D}$ = -25 (*c* 0.98, CHCl$_3$, 86:14 er)

HRMS (ESI+) *m/z* calculated for $C_{17}H_{16}N_2O_6S$+Na$^+$ [M+Na]$^+$ 399.06213, found 399.06135.

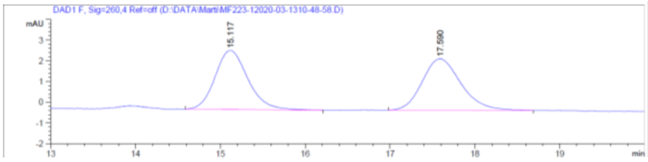

Signal 2: DAD1 F, Sig=260,4 Ref=off

Peak #	RetTime [min]	Type	Width [min]	Area [mAU*s]	Height [mAU]	Area %
1	15.117	BB	0.4050	76.44444	2.84929	49.1894
2	17.590	BB	0.4706	78.96404	2.48583	50.8106
Totals :				155.40848	5.33512	

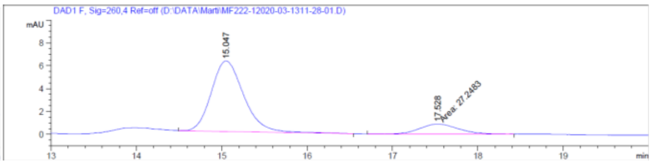

Signal 2: DAD1 F, Sig=260,4 Ref=off

Peak #	RetTime [min]	Type	Width [min]	Area [mAU*s]	Height [mAU]	Area %
1	15.047	BB	0.4033	162.61902	6.17470	85.6488
2	17.528	MM	0.5209	27.24834	8.71851e-1	14.3512
Totals :				189.86736	7.04655	

(*R*)-1-((4-Nitrophenyl)sulfonyl)-4-phenylpyrrolidin-2-one (4db)

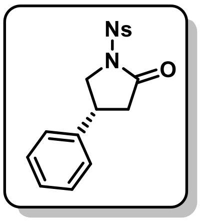

Following General Procedure (ii). Colorless oil.

Run 1: (78.9 mg, 0.228 mmol, 76% yield). **Run 2:** (70.6 mg, 0.204 mmol, 68% yield). **Average: 72% yield**.

The enantiomeric ratio was determined by HPLC analysis in comparison to a racemic material using Daicel Chiralpak® IB column (4.6 mm × 250 mm) at 30 °C and 50:50 hexanes:iPrOH (1.3 mL min^{-1}) as mobile phase, λ = 260 nm (t_r = 17.8 min (major), 20.5 min (minor)).

^{1}H NMR (500 MHz, CDCl$_3$) δ 8.39 (d, *J* = 8.7 Hz, 2H), 8.25 (d, *J* = 8.8 Hz, 2H), 7.38 - 7.28 (m, 3H), 7.19 – 7.07 (m, 2H), 4.38 (dd, *J* = 9.9, 8.0 Hz, 1H), 3.85 (dd, *J* = 9.9, 8.0 Hz, 1H), 3.67 (quint, *J* = 8.2, 1H), 2.89 (dd, *J* = 17.5, 8.4 Hz, 1H), 2.68 (dd, *J* = 17.5, 9.2 Hz, 1H).

^{13}C NMR (126 MHz, CDCl$_3$) δ 172.28, 150.96, 143.33, 139.28, 129.61, 129.19, 127.93, 126.52, 124.30, 53.93, 39.19, 37.36.

[α]$^{20}_{D}$ = -27 (*c* 1.02, CHCl$_3$, 80:20 er)

HRMS (ESI+) *m/z* calculated for $C_{16}H_{14}N_2O_5S$+H$^+$ [M+H]$^+$ 347.06962, found 347.06940.

HRMS (ESI+) *m/z* calculated for $C_{16}H_{14}N_2O_5S$+Na$^+$ [M+Na]$^+$ 369.05156, found 369.05130.

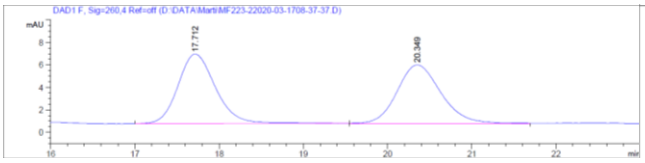

Signal 3: DAD1 F, Sig=260,4 Ref=off

Peak #	RetTime [min]	Type	Width [min]	Area [mAU*s]	Height [mAU]	Area %
1	17.712	BB	0.4669	191.30092	6.18395	50.6470
2	20.349	BB	0.5349	186.41341	5.19224	49.3530
Totals :				377.71432	11.37619	

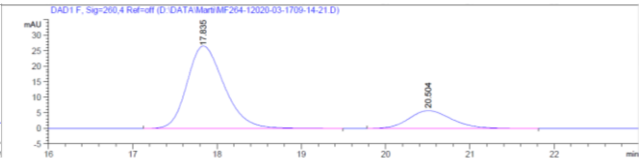

Signal 3: DAD1 F, Sig=260,4 Ref=off

Peak #	RetTime [min]	Type	Width [min]	Area [mAU*s]	Height [mAU]	Area %
1	17.835	BB	0.4700	812.38898	26.62169	80.0788
2	20.504	BB	0.5225	202.09770	5.74330	19.9212
Totals :				1014.48668	32.36499	

(R)-1-((4-Nitrophenyl)sulfonyl)-4-(4-(trifluoromethyl)phenyl)pyrrolidin-2-one (4dc)

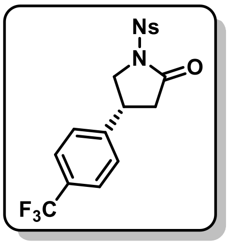

Following General Procedure (ii). Colorless solid.

Run 1: (95.6 mg, 0.230 mmol, 77% yield). **Run 2:** (88.7 mg, 0.214 mmol, 71% yield). **Average: 74% yield**.

The enantiomeric ratio was determined by HPLC analysis in comparison to a racemic material using Daicel Chiralpak® IB column (4.6 mm × 250 mm) at 30 °C and 50:50 hexanes:iPrOH (1.3 mL min^{-1}) as mobile phase, λ = 260 nm (t_r = 11.9 min (major), 15.9 min (minor)).

^{1}H NMR (250 MHz, $CDCl_3$) δ 8.41 (d, J = 8.9 Hz, 2H), 8.28 (d, J = 8.9 Hz, 2H), 7.62 (d, J = 8.2 Hz, 2H), 7.31 (d, J = 8.2 Hz, 2H), 4.40 (dd, J = 10.0, 7.9 Hz, 1H), 3.86 (dd, J = 10.0, 7.9 Hz, 1H), 3.80 – 3.68 (m, 1H), 2.93 (dd, J = 17.5, 8.4 Hz, 1H), 2.69 (dd, J = 17.5, 9.2 Hz, 1H).

^{13}C NMR (250 MHz, $CDCl_3$) δ 171.57, 151.06, 143.22, 143.16, 130.41 (q, J = 32.9 Hz), 129.68, 127.03, 126.21 (q, 3.6 Hz), 124.84, 124.35, 122.68, 120.51, 53.31, 39.00, 37.14.

^{19}F NMR (470 MHz, $CDCl_3$) δ -62.74.

$[\alpha]^{20}_D$ = +21 (c 0.50, MeOH, 83:17 er)

HRMS (ESI+) m/z calculated for $C_{17}H_{13}F_3N_2O_5S$+H^+ $[M+H]^+$ 415.05700, found 415.05667.

HRMS (ESI+) m/z calculated for $C_{17}H_{13}F_3N_2O_5S$ +Na^+ $[M+Na]^+$ 437.03895, found 369.03848.

mp: 189-191°C.

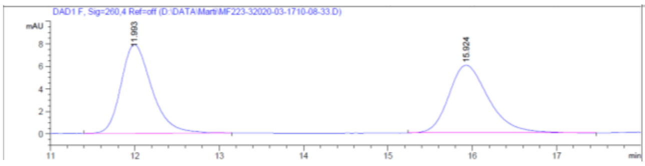

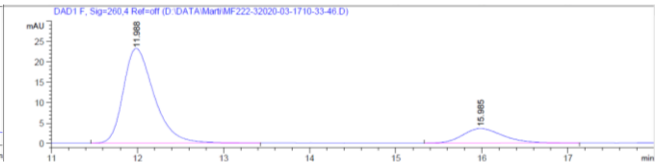

Signal 3: DAD1 F, Sig=260,4 Ref=off

Peak #	RetTime [min]	Type	Width [min]	Area [mAU*s]	Height [mAU]	Area %
1	11.993	BB	0.3827	198.98071	7.87601	50.1625
2	15.924	BB	0.4892	197.69135	6.01902	49.8375
Totals :				396.67206	13.89503	

Signal 3: DAD1 F, Sig=260,4 Ref=off

Peak #	RetTime [min]	Type	Width [min]	Area [mAU*s]	Height [mAU]	Area %
1	11.988	BB	0.3778	577.20740	23.23417	83.1789
2	15.985	BB	0.4770	116.72707	3.55584	16.8211
Totals :				693.93446	26.79000	

(R)-4-(4-Chlorophenyl)-1-((4-nitrophenyl)sulfonyl)pyrrolidin-2-one (4dd)

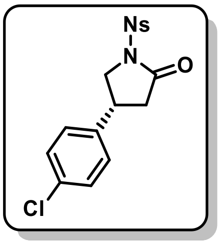

Following General Procedure (ii). Clear-yellow solid.

Run 1: (92.6 mg, 0.243 mmol, 81% yield). **Run 2:** (93.5 mg, 0,245 mmol, 81% yield). **Average: 81% yield**.

The enantiomeric ratio was determined by HPLC analysis in comparison to a racemic material using Daicel Chiralpak® IB-3 column (4.6 mm × 250 mm) at 30 °C and 60:40 hexanes:iPrOH (1.3 mL min^{-1}) as mobile phase, λ = 260 nm (t_r = 17.4 min (major), 24.3 min (minor)).

^{1}H NMR (500 MHz, $CDCl_3$) δ 8.40 (d, J = 8.8 Hz, 2H), 8.25 (d, J = 8.8 Hz, 2H), 7.31 (d, J = 8.4 Hz, 2H), 7.09 (d, J = 8.4 Hz, 2H), 4.35 (dd, J = 9.9, 7.9 Hz, 1H), 3.80 (dd, J = 9.9, 8.0 Hz, 1H), 3.63 (quint, J = 8.0 Hz, 1H), 2.87 (dd, J = 17.5, 8.4 Hz, 1H), 2.63 (dd, J = 17.5, 9.2 Hz, 1H).

^{13}C NMR (126 MHz, $CDCl_3$) δ 171.9, 151.2, 143.4, 137.8, 134.0, 129.8, 129.5, 128.0, 124.5, 53.8, 39.3, 37.0.

$[\alpha]^{20}_{D}$ = -2 (*c* 1.02, MeOH, 88:12 er)

HRMS (ESI+) *m/z* calculated for $C_{16}H_{13}ClN_2O_5S+H^+$ $[M+H]^+$ 381.03065, found 381.03015.

HRMS (ESI+) *m/z* calculated for $C_{16}H_{13}ClN_2O_5S+Na^+$ $[M+Na]^+$ 403.01259, found 403.01221.

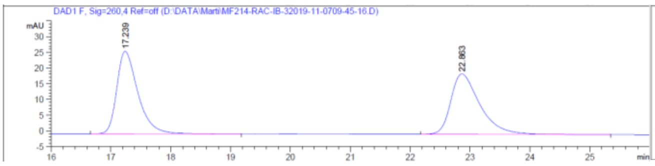

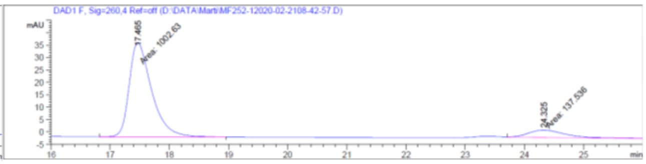

Signal 3: DAD1 F, Sig=260,4 Ref=off

Peak #	RetTime [min]	Type	Width [min]	Area [mAU*s]	Height [mAU]	Area %
1	17.239	BB	0.3716	648.75983	26.31353	49.9012
2	22.863	BB	0.5048	651.32800	19.24595	50.0988
Totals :				1300.08783	45.55948	

Signal 3: DAD1 F, Sig=260,4 Ref=off

Peak #	RetTime [min]	Type	Width [min]	Area [mAU*s]	Height [mAU]	Area %
1	17.465	MM	0.4386	1002.62891	38.10291	87.9372
2	24.325	MM	0.7648	137.53607	2.99707	12.0628
Totals :				1140.16498	41.09999	

(*R*)-4-(3-(Cyclopentyloxy)-4-methoxyphenyl)-1-((4-nitrophenyl)sulfonyl)pyrrolidin-2-one (4de)

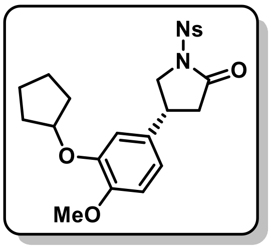

Following General Procedure (ii). Yellow solid.

Run 1: (90.3 mg, 0.196 mmol, 65% yield, 99:1 *er*). **Run 2:** (84.9 mg, 0,184 mmol, 61% yield). **Average: 63% yield**.

The enantiomeric ratio was determined by HPLC analysis in comparison to a racemic material using Daicel Chiralpak® IC column (4.6 mm × 250 mm) at 30 °C and 50:50 hexanes:iPrOH (1.3 mL min^{-1}) as mobile phase, λ = 225 nm (t_r = 17.6 min (major), 24.3 min (minor)).

^{1}H NMR (400 MHz, $CDCl_3$) δ 8.37 (d, *J* = 8.9 Hz, 2H), 8.22 (d, *J* = 8.9 Hz, 2H), 6.77 (d, *J* = 7.9 Hz, 1H), 6.68 – 6.59 (m, 2H), 4.70 (m, 1H), 4.32 (dd, *J* = 9.9, 7.7 Hz, 1H), 3.81(s, 3H), 3.80 (m, 1H) 3.57 (quint, *J* = 8.0 Hz, 1H), 2.84 (dd, *J* = 17.5, 8.3 Hz, 1H), 2.63 (dd, *J* = 17.5, 8.9 Hz, 1H), 1.97 – 1.61 (m, 8H).

^{13}C NMR (101 MHz, $CDCl_3$) δ 172.5, 151.0, 149.9, 148.2, 143.5, 131.8, 129.7, 124.4, 118.6, 113.7, 112.4, 80.8, 56.2, 54.3, 39.4, 37.1, 32.9, 32.9, 24.1.

HRMS (ESI+) *m/z* calculated for $C_{22}H_{24}N_2O_7S+H^+$ $[M+H]^+$ 461.13770, found 461.13718.

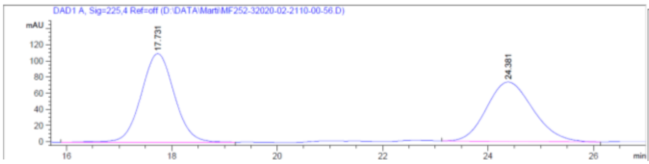

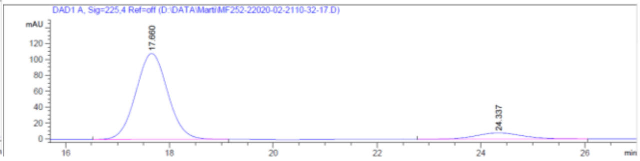

Signal 1: DAD1 A, Sig=225,4 Ref=off

Peak #	RetTime [min]	Type	Width [min]	Area [mAU*s]	Height [mAU]	Area %
1	17.731	BB	0.6773	4790.33691	109.73964	51.6142
2	24.381	BB	0.9366	4490.70703	73.52392	48.3858
Totals :				9281.04395	183.26356	

Signal 1: DAD1 A, Sig=225,4 Ref=off

Peak #	RetTime [min]	Type	Width [min]	Area [mAU*s]	Height [mAU]	Area %
1	17.660	BB	0.6599	4595.22852	108.10003	90.7350
2	24.337	BB	0.7938	469.21887	7.66864	9.2650
Totals :				5064.44739	115.76867	

(*R*)-4-(4-Chlorophenyl)pyrrolidin-2-one (5a)

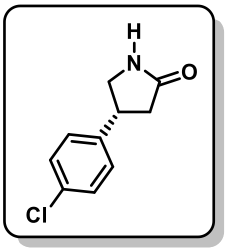

4dd (75 mg, 0.20 mmol) was added to the suspension of K_2CO_3 (55 mg, 0.39 mmol) in CH_3CN (2 mL), then PhSH (31 µL, 0.3 mmol) was added at room temperature. Next, DMSO (0.75 mL) was added to the reaction mixture, and stirring was continued at room temperature for 2 h. After complete consumption of the starting compound, the reaction was quenched with water and the aqueous layer was extracted with ethyl acetate. The residue was purified by flash column chromatography using hexanes/MeOH as eluent to provide the compound **5a** (79%, 31 mg, 0.158 mmol). Off-white solid.

The enantiomeric ratio was determined by HPLC analysis in comparison to a racemic material using Daicel Chiralpak® OJ-H column (4.6 mm × 250 mm) at 30 °C and 90:10 hexanes:iPrOH (1.0 mL min^{-1}) as mobile phase, λ = 225 nm (t_r = 15.3 min (major), 17.4 min (minor)).

^{1}H NMR (500 MHz, $CDCl_3$) δ 7.32 (d, *J* = 8.5 Hz, 2H), 7.19 (d, *J* = 8.5 Hz, 2H), 6.31 (bs, 1H), 3.79 (t, *J* = 8.8 Hz, 1H), 3.63 (quint, *J* = 8.0 Hz, 1H), 3.38 (dd, *J* = 9.4, 7.1 Hz, 1H), 2.74 (dd, *J* = 16.9, 8.9 Hz, 1H), 2.46 (dd, *J* = 16.9, 8.6 Hz, 1H).

^{13}C NMR (126 MHz, $CDCl_3$) δ 177.3, 140.6, 132.9, 129.0, 128.1, 49.4, 39.7, 37.8.

$[\alpha]^{20}_D$ = -13 (*c* 1.0, MeOH, 90:10 er)

HRMS (ESI+) *m/z* calculated for $C_{10}H_{10}NClO+H^+$ $[M+H]^+$ 196.05237, found 196.05255.

mp: 105-107°C.

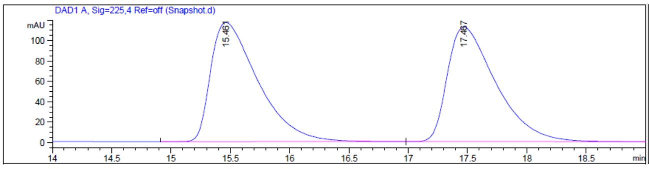

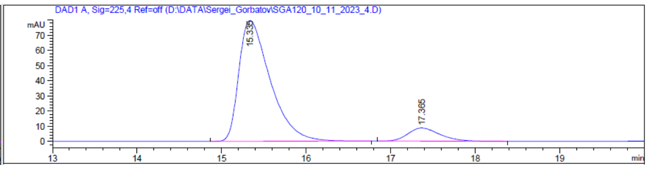

Signal 1: DAD1 A, Sig=225,4 Ref=off

Peak #	RetTime [min]	Type	Width [min]	Area [mAU*s]	Height [mAU]	Area %
1	15.461	BB	0.4209	3230.46167	116.71989	50.2910
2	17.467	BB	0.4325	3193.08301	112.03905	49.7090
Totals :				6423.54468	228.75894	

Signal 1: DAD1 A, Sig=225,4 Ref=off

Peak #	RetTime [min]	Type	Width [min]	Area [mAU*s]	Height [mAU]	Area %
1	15.335	BB	0.3899	2045.66260	79.58565	89.9989
2	17.365	BB	0.4023	227.32468	8.65915	10.0011
Totals :				2272.98727	88.24479	

(*R*)-4-(3-(Cyclopentyloxy)-4-methoxyphenyl)-1-((4-nitrophenyl)sulfonyl)pyrrolidin-2-one (5b)

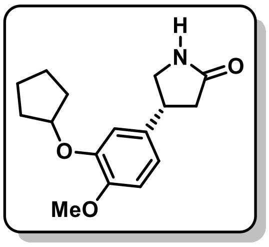

4de (48.5 mg, 0.105 mmol) was added to the suspension of K_2CO_3 (29 mg, 0.210 mmol) in CH_3CN (1 mL), then PhSH (31 µL, 0.154 mmol) was added at room temperature. Next, DMSO (0.4 mL) was added to the reaction mixture, and stirring was continued at room temperature for 2 h. After complete consumption of the starting compound, the reaction was quenched with water and the aqueous layer was extracted with ethyl acetate. The residue was purified by flash column chromatography using hexanes/MeOH as eluent to provide the compound **5b** (97%, 28 mg, 0.102 mmol). White solid.

The enantiomeric ratio was determined by HPLC analysis in comparison to a racemic material using Daicel Chiralpak® IC column (4.6 mm × 250 mm) at 30 °C and 65:35 hexanes:iPrOH (1.0 mL min^{-1}) as mobile phase, λ = 210 nm (t_r = 20.4 min (minor), 23.3 min (major)).

^{1}H NMR (500 MHz, $CDCl_3$) δ 6.83 (dd, *J* = 7.9, 2.1 Hz, 1H), 6.80 – 6.75 (m, 2H), 6.43 (bs, 1H), 4.81 – 4.73 (m, 1H), 3.83 (s, 3H), 3.76 (t, *J* = 8.9 Hz, 1H), 3.64 (quint, *J* = 8.4 Hz, 1H),, 3.42 – 3.35 (m, 1H), 2.71 (dd, *J* = 16.9, 2.0 Hz, 1H), 2.48 (dd, *J* = 16.9, 2.1 Hz, 1H), 1.98 – 1.78 (m, 6H), 1.67 – 1.56 (m, 2H).
^{13}C NMR (126 MHz, $CDCl_3$) δ 177.8, 149.2, 147.9, 134.6, 118.8, 113.9, 112.2, 80.6, 56.2, 49.8, 40.0, 38.1, 32.8, 24.0.
$[\alpha]^{20}_D$ = -15 (*c* 1.0, MeOH, 82:18 er)
HRMS (ESI+) *m/z* calculated for $C_{16}H_{21}NO_3+H^+$ $[M+H]^+$ 276.15942, found 276.15926.
mp: 130-132°C.

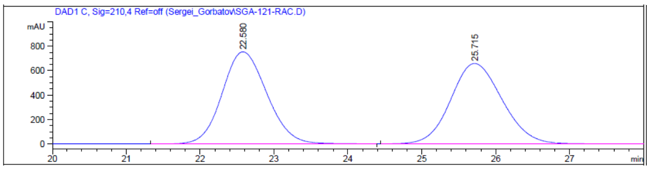

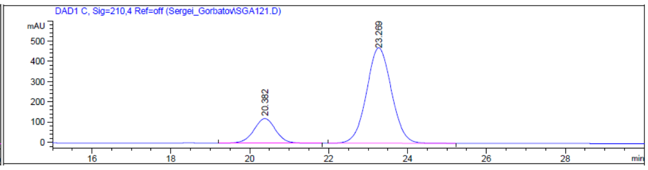

Signal 3: DAD1 C, Sig=210,4 Ref=off

Peak #	RetTime [min]	Type	Width [min]	Area [mAU*s]	Height [mAU]	Area %
1	22.580	BB	0.6538	3.18280e4	755.02258	49.8513
2	25.715	BB	0.7526	3.20178e4	659.21533	50.1487
Totals :				6.38458e4	1414.23792	

Signal 3: DAD1 C, Sig=210,4 Ref=off

Peak #	RetTime [min]	Type	Width [min]	Area [mAU*s]	Height [mAU]	Area %
1	20.382	BB	0.5917	4643.79785	121.54782	18.3587
2	23.269	BB	0.6794	2.06510e4	471.10315	81.6413
Totals :				2.52948e4	592.65097	

(*R*)-Baclofen hydrochloride (6)

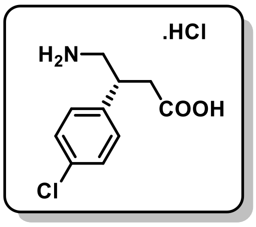

A mixture of compound **5a** (30 mg, 0.153 mmol) in aqueous 6 N HCl (0.5 ml) was heated at 100°C for 10 h. The excess of water in the reaction mixture was removed under reduced pressure to obtain solid residue, which was triturated in isopropanol affording (*R*)-baclofen hydrochloride **6** (76%, 29 mg, 0.116 mmol). White solid.
^{1}H NMR (500 MHz, D_2O) δ 7.37 (d, *J* = 8.4 Hz, 2H), 7.27 (d, *J* = 8.4 Hz, 2H), 3.41 – 3.28 (m, 2H), 3.18 (dd, *J* = 12.7, 10.4 Hz, 1H), 2.79 (dd, *J* = 16.1, 5.8 Hz, 1H), 2.67 (dd, *J* = 16.1, 8.9 Hz, 1H).
^{13}C NMR (126 MHz, D_2O) δ 175.3, 137.0, 133.4, 129.4, 129.3, 43.6, 39.4, 38.2.
HRMS (ESI+) *m/z* calculated for $C_{10}H_{12}ClNO_2+H^+$ $[M+H]^+$ 214.06286, found 214.06293.
It was not possible to determine the optical rotation $[\alpha]^{20}_D$ of the compound due to its low response on the polarimeter.
Separation of the enantiomers of **6** by normal-phase HPLC was also attempted, but it failed due to its high polarity and low absorbance (DAD detector).
mp: 194-196°C (decomposition).

4. ^{1}H, ^{13}C and ^{19}F NMR Spectra

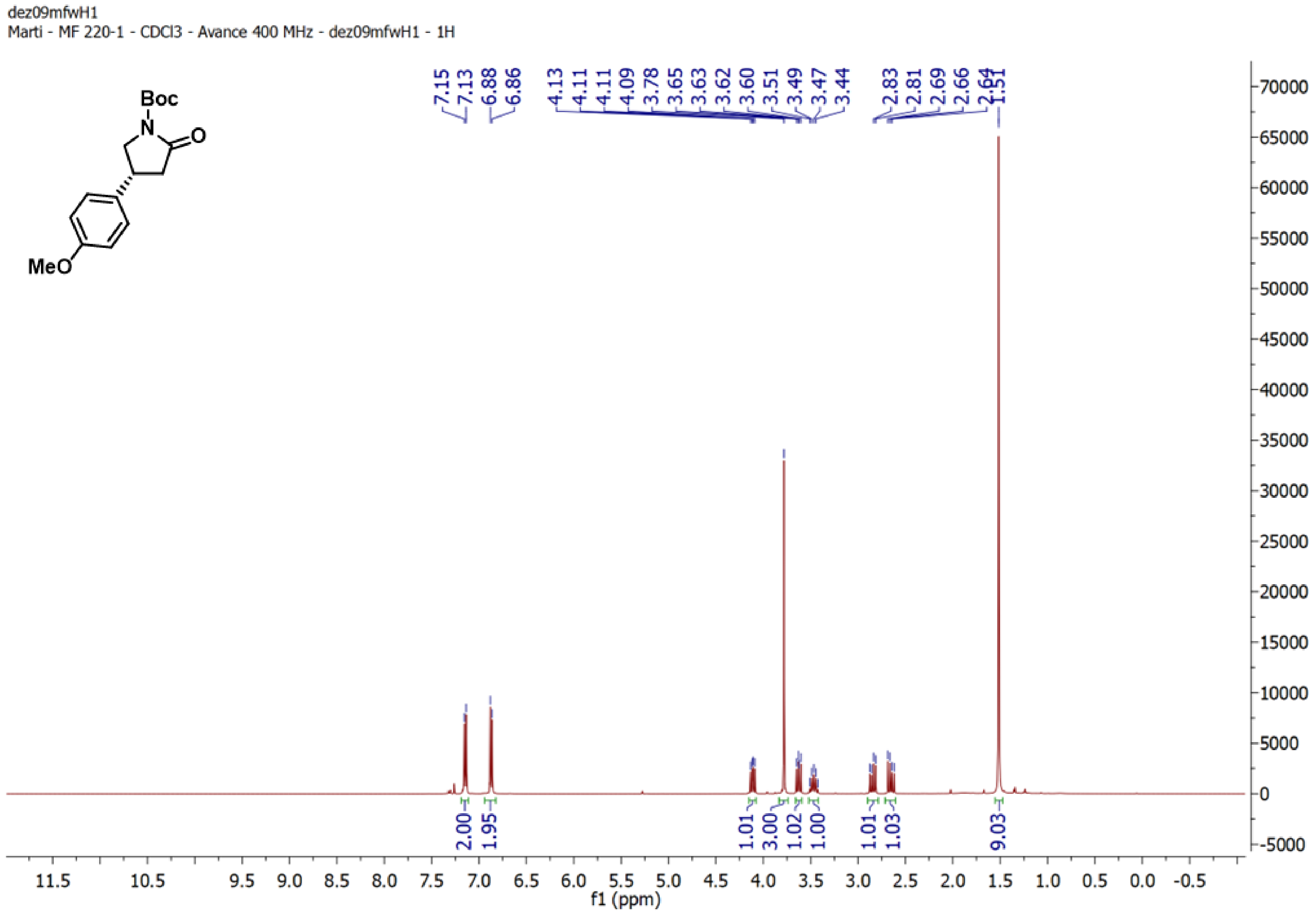

Figure SI8: ^{1}H NMR of compound **4aa**.

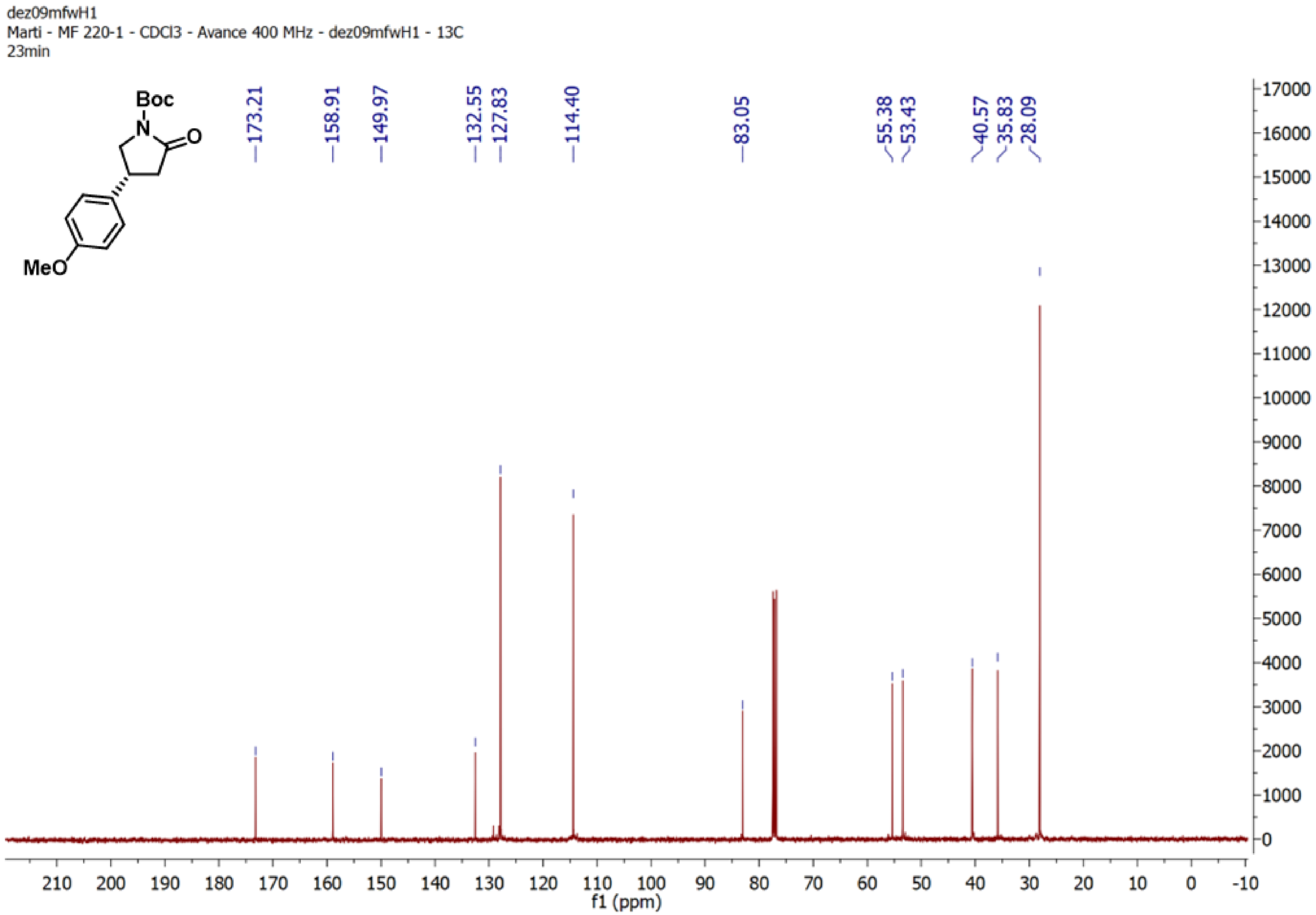

Figure SI9: ^{13}C NMR of compound **4aa**.

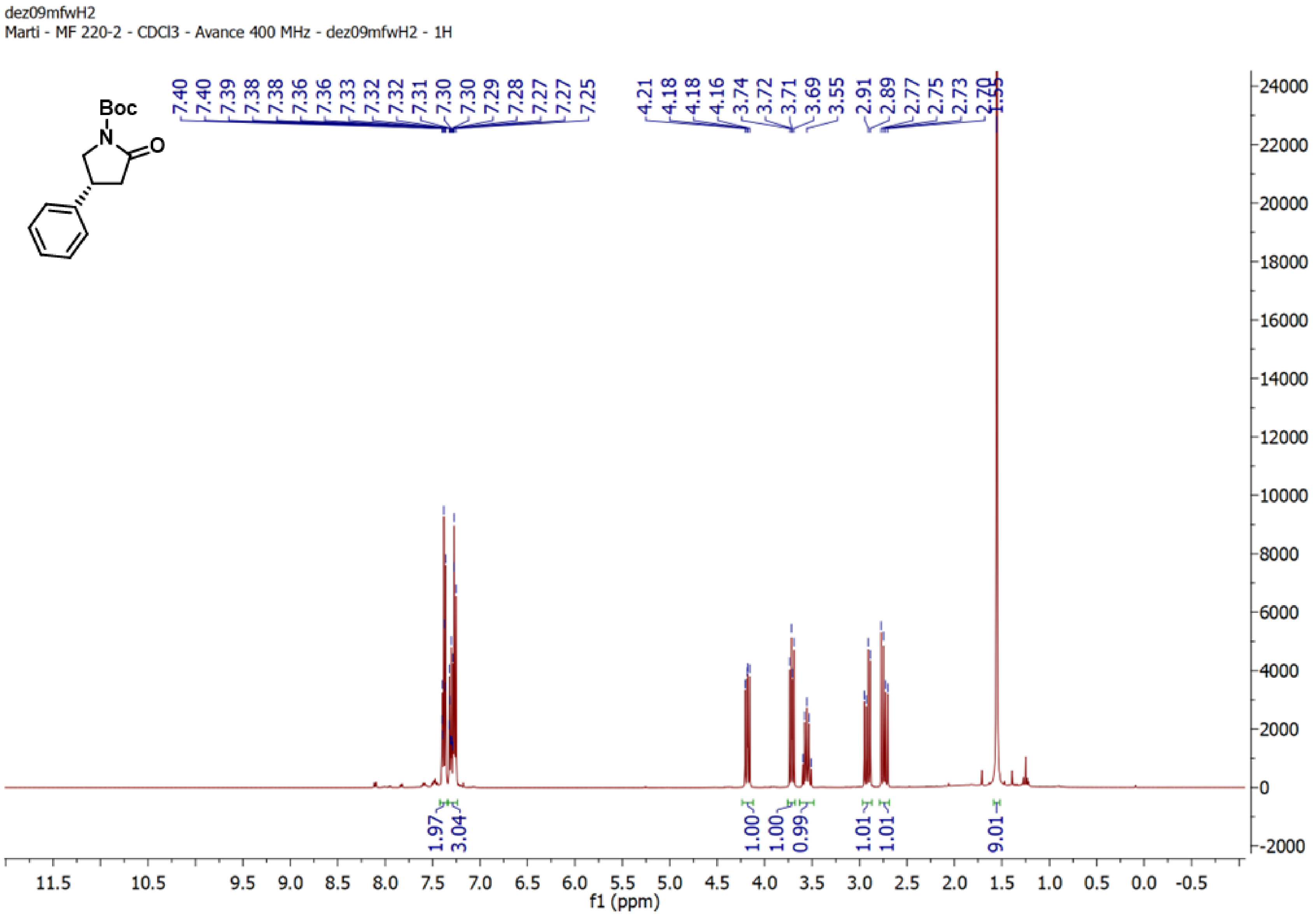

Figure SI10: ^{1}H NMR of compound **4ab**.

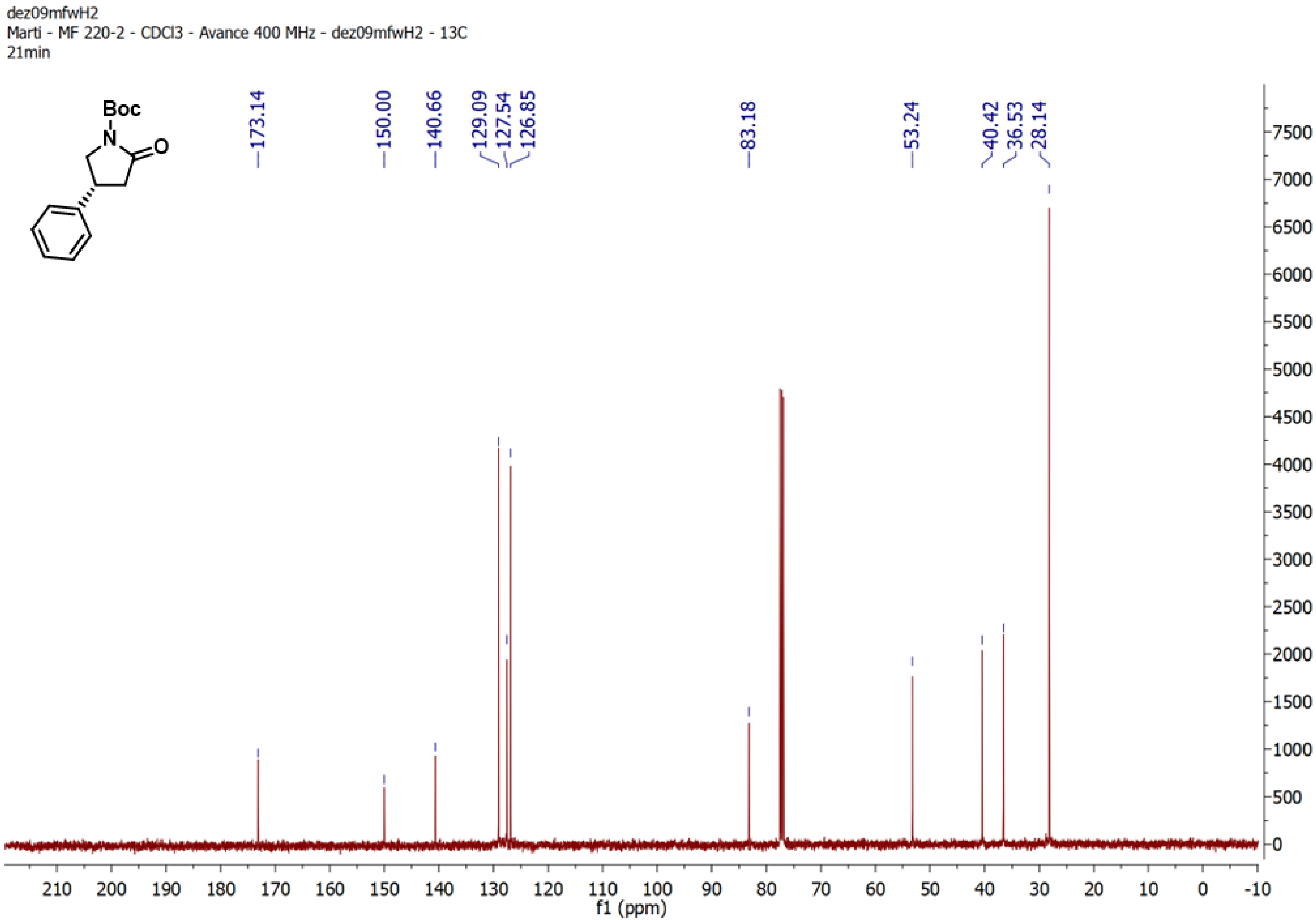

Figure SI11: ^{13}C NMR of compound **4ab**.

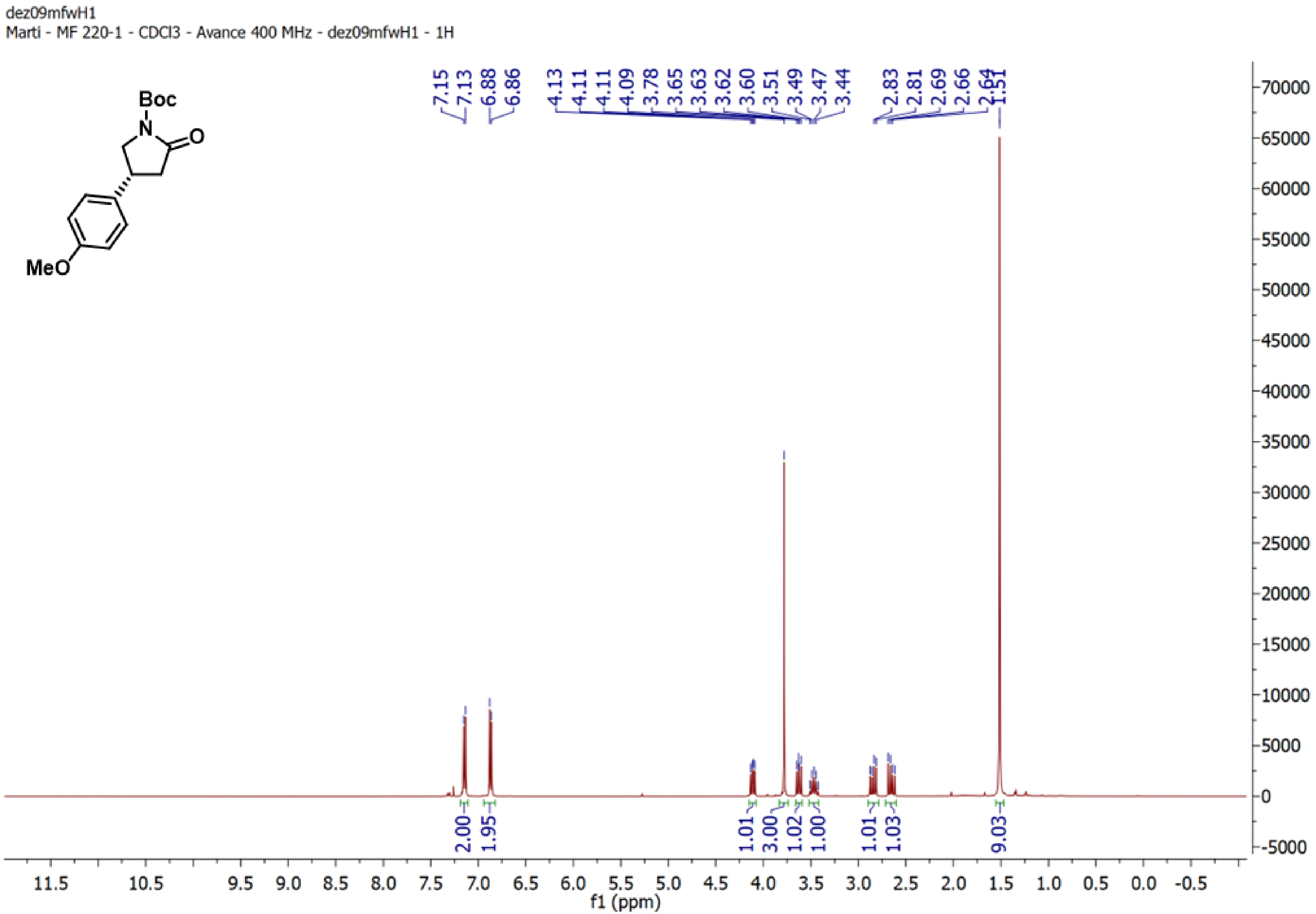

Figure SI12: ^{1}H NMR of compound **4ac**.

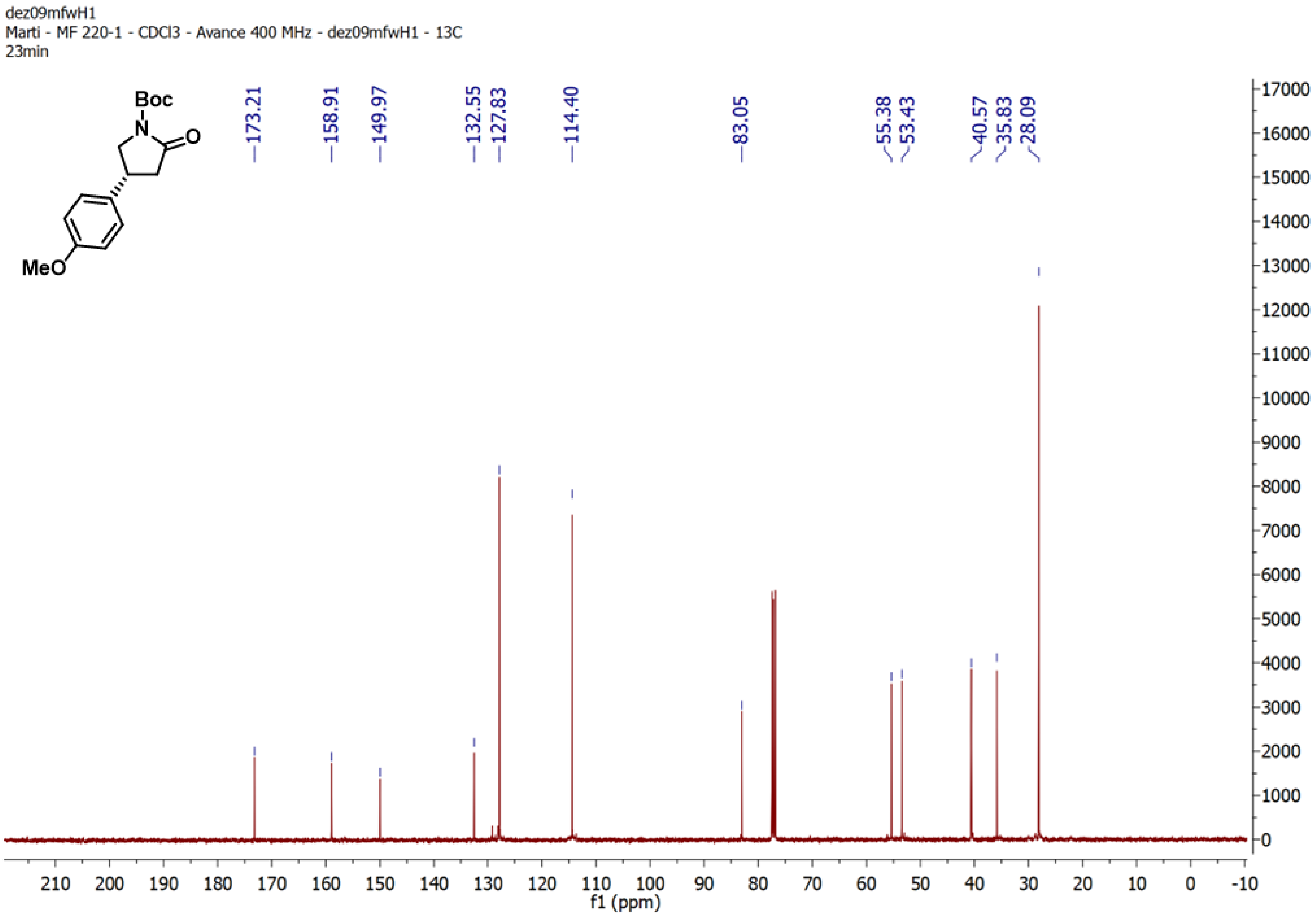

Figure SI13: ^{13}C NMR of compound **4ac**.

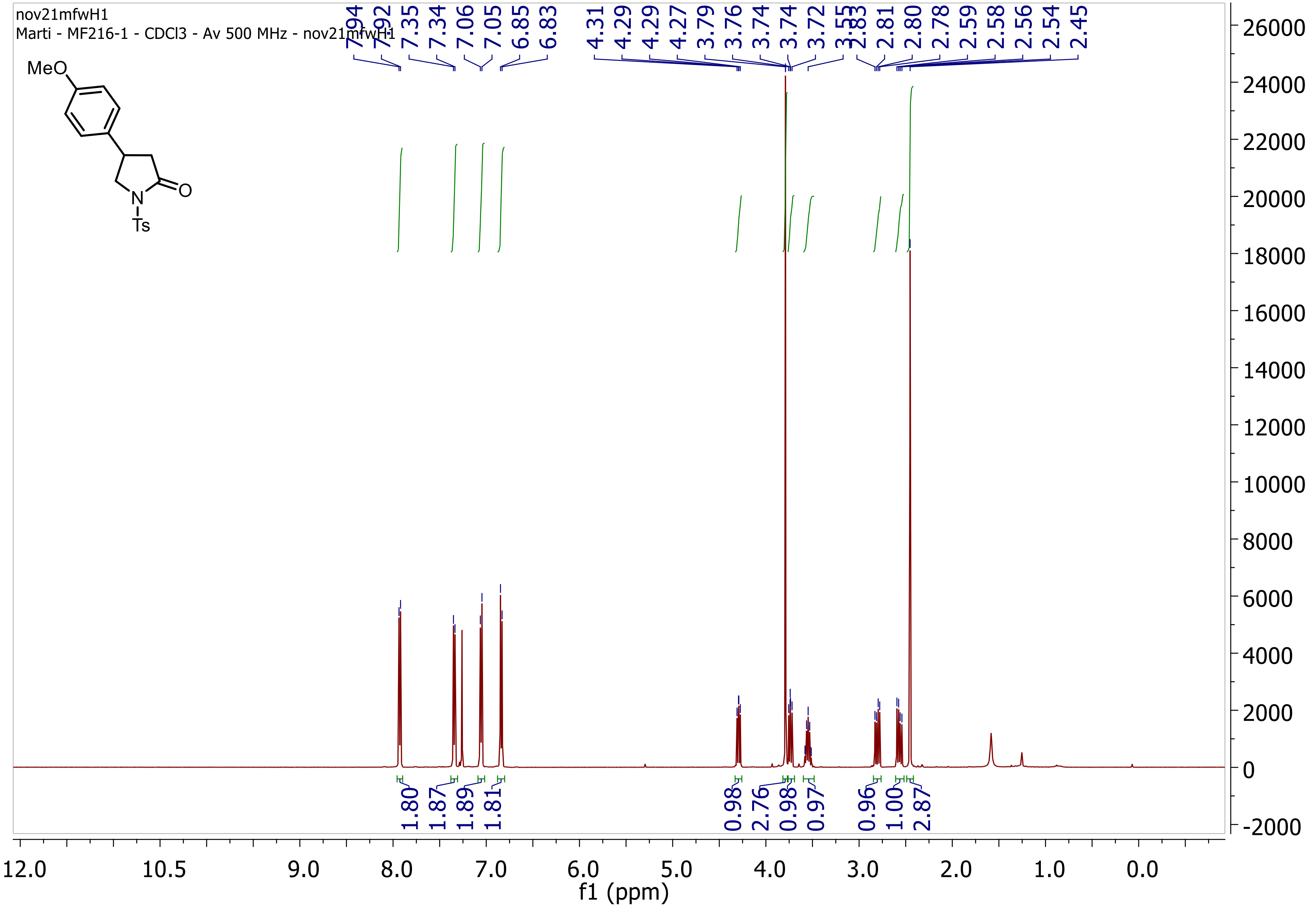

Figure SI14: ^{1}H NMR of compound **4ba**.

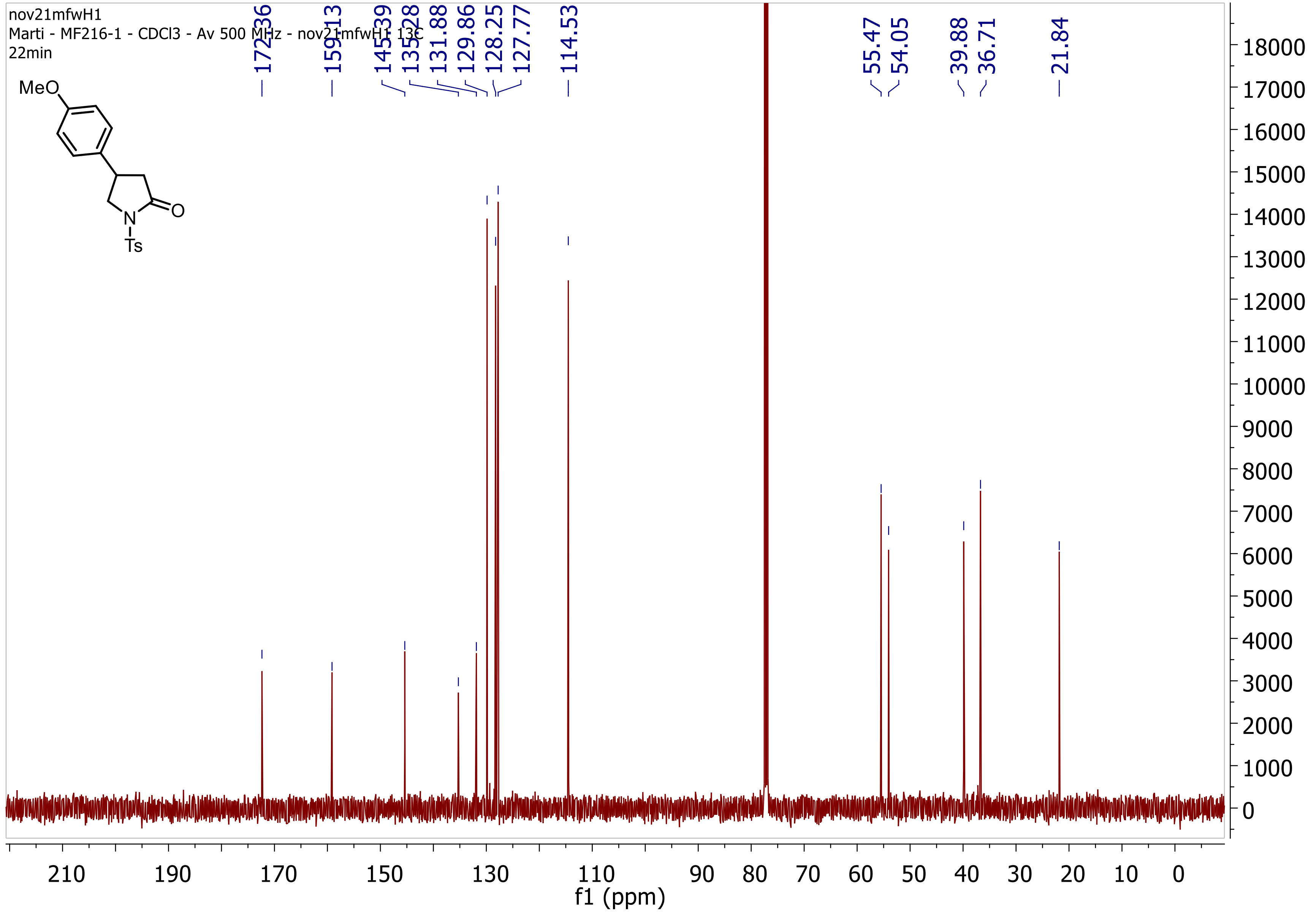

Figure SI15: ^{13}C NMR of compound **4ba**.

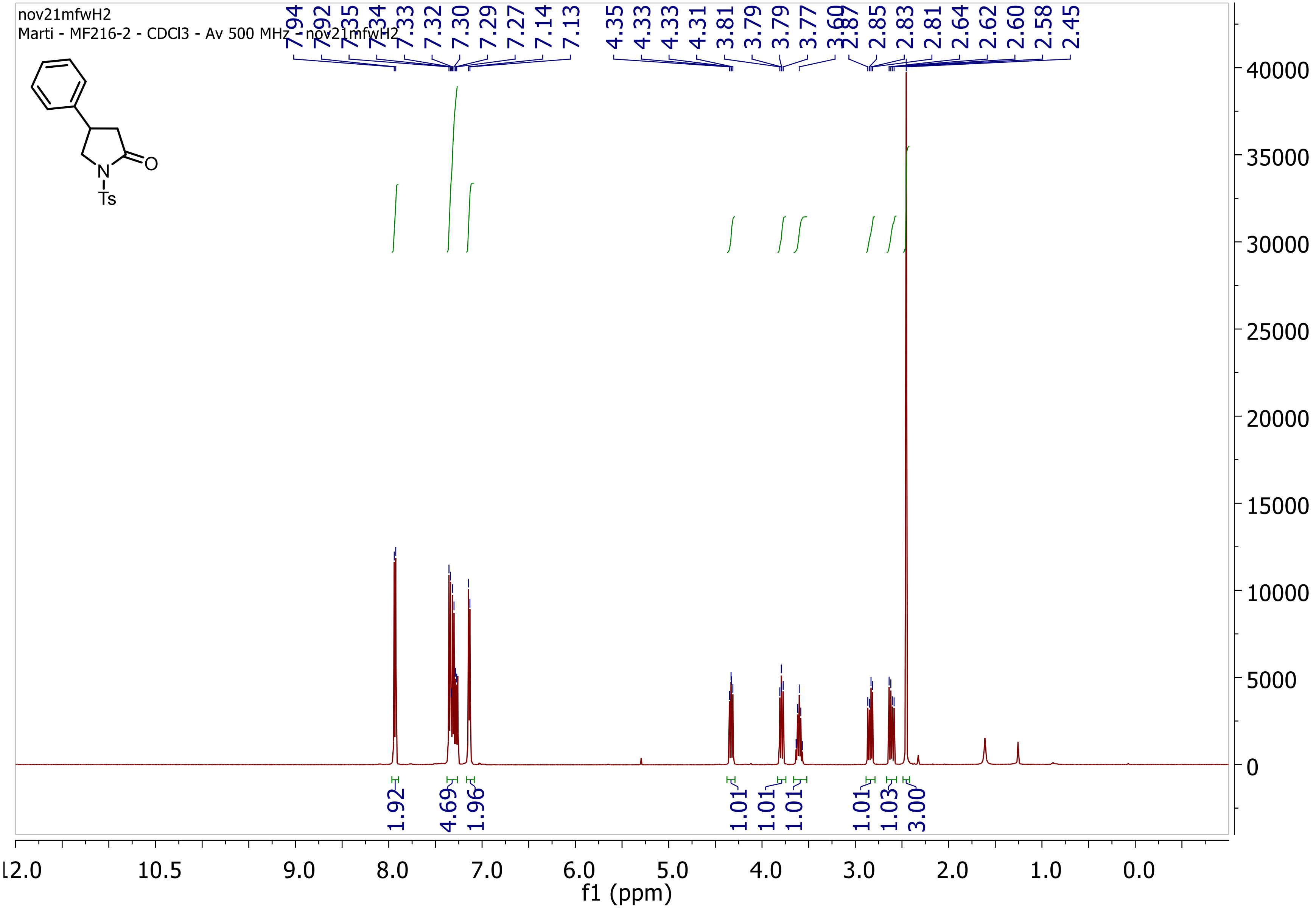

Figure SI16: ^{1}H NMR of compound **4bb**.

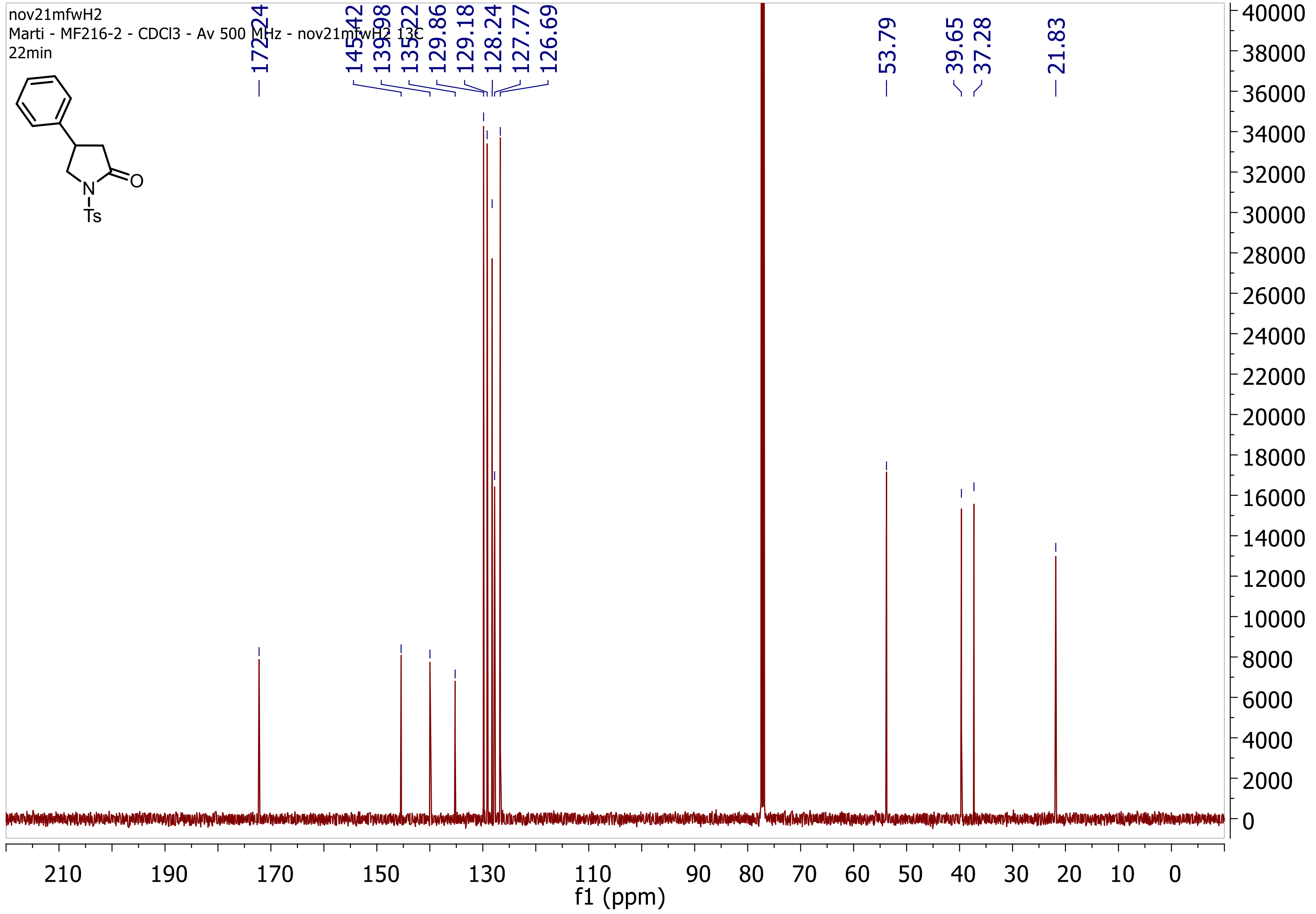

Figure SI17: ^{13}C NMR of compound **4bb**.

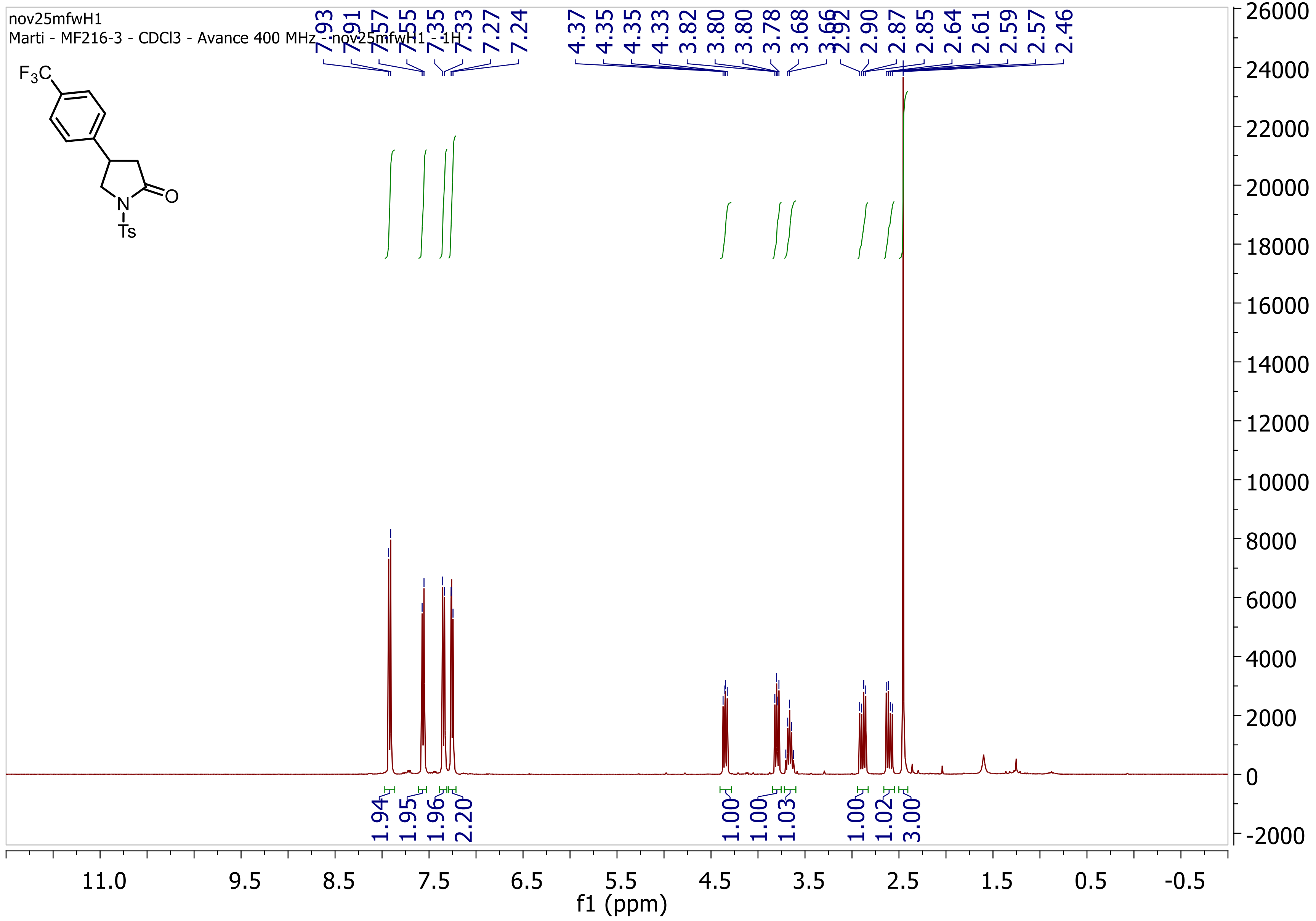

Figure SI18: ^{1}H NMR of compound **4bc**.

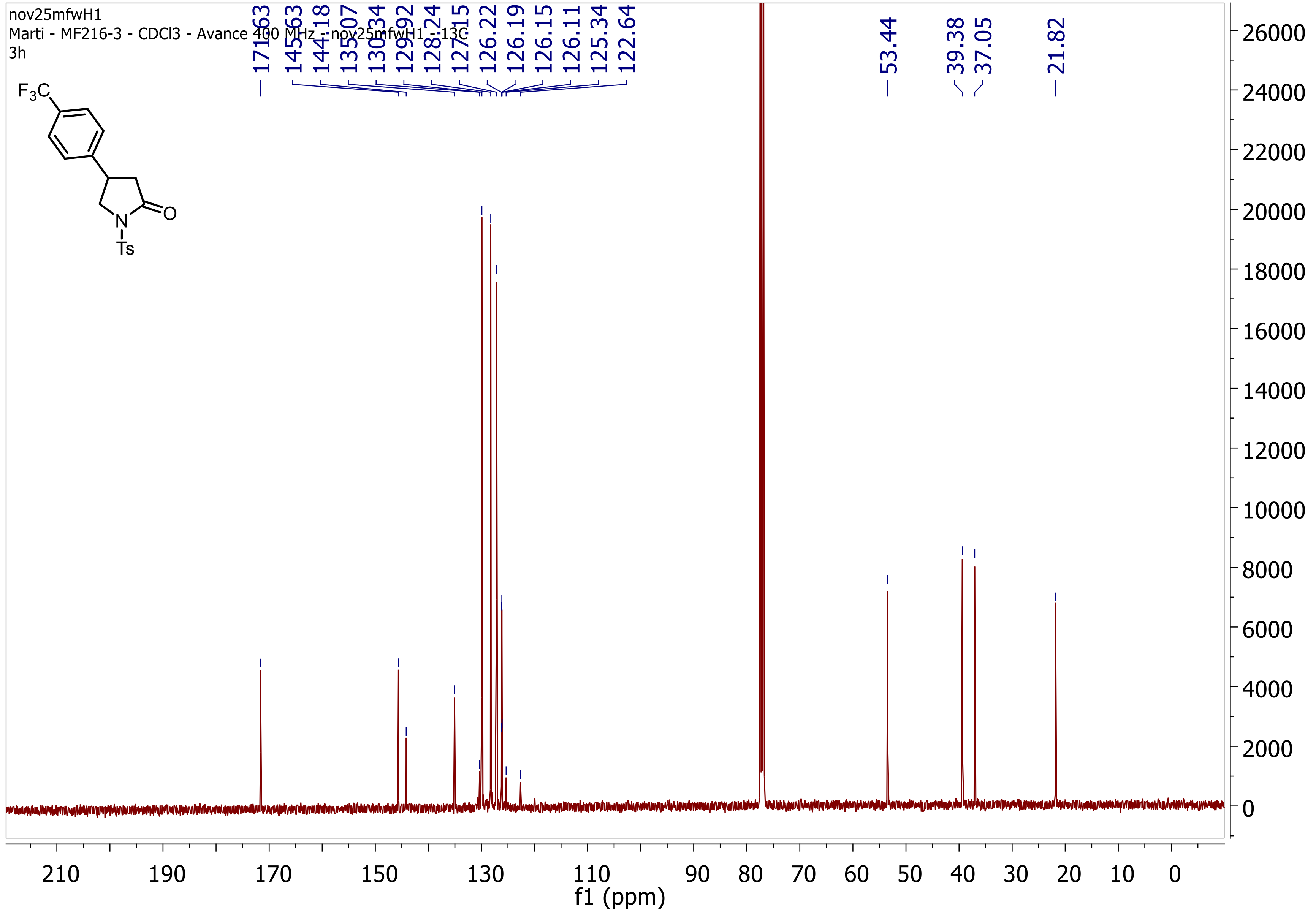

Figure SI19: ^{13}C NMR of compound **4bc**.

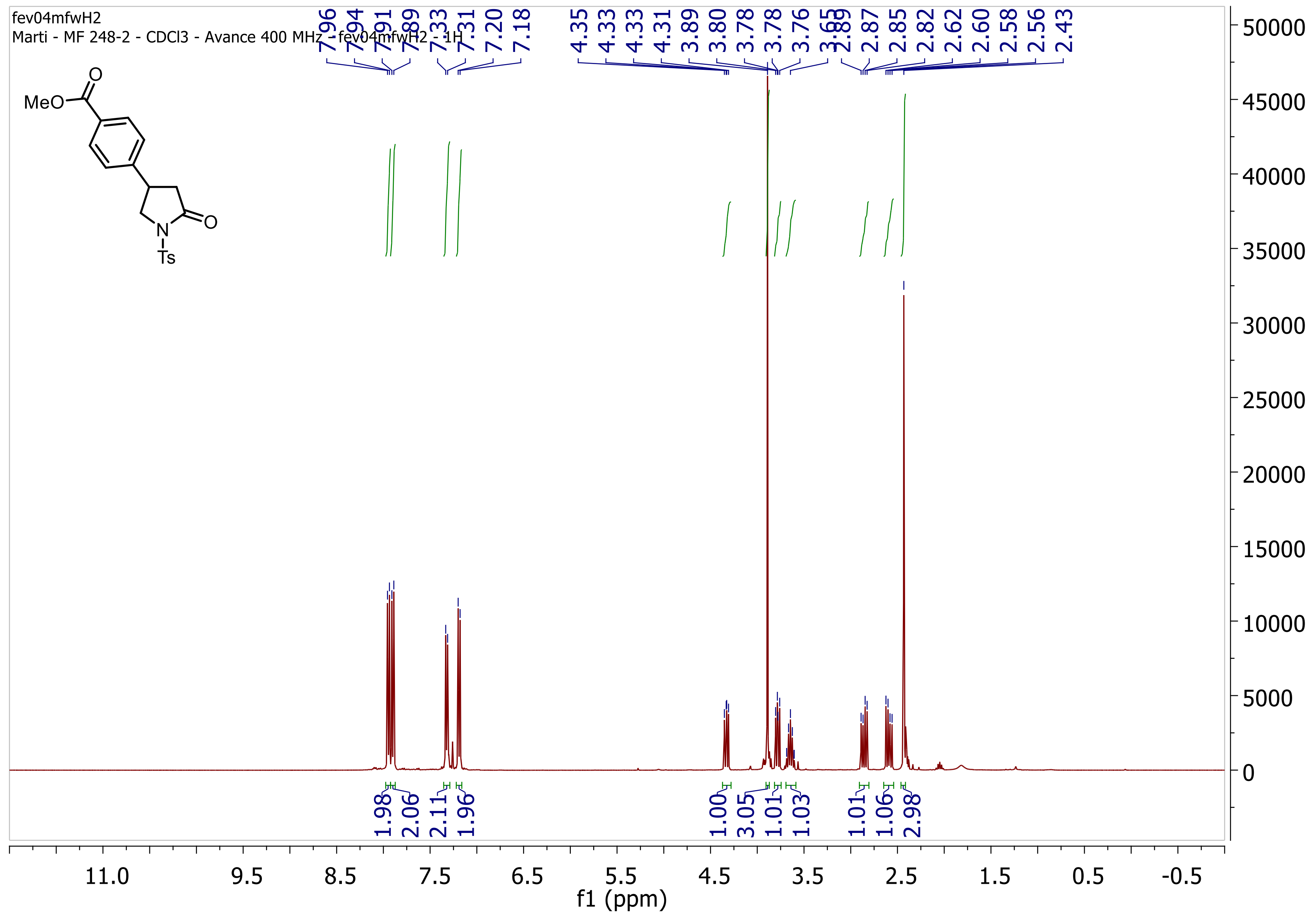

Figure SI20: ^{1}H NMR of compound **4bd**.

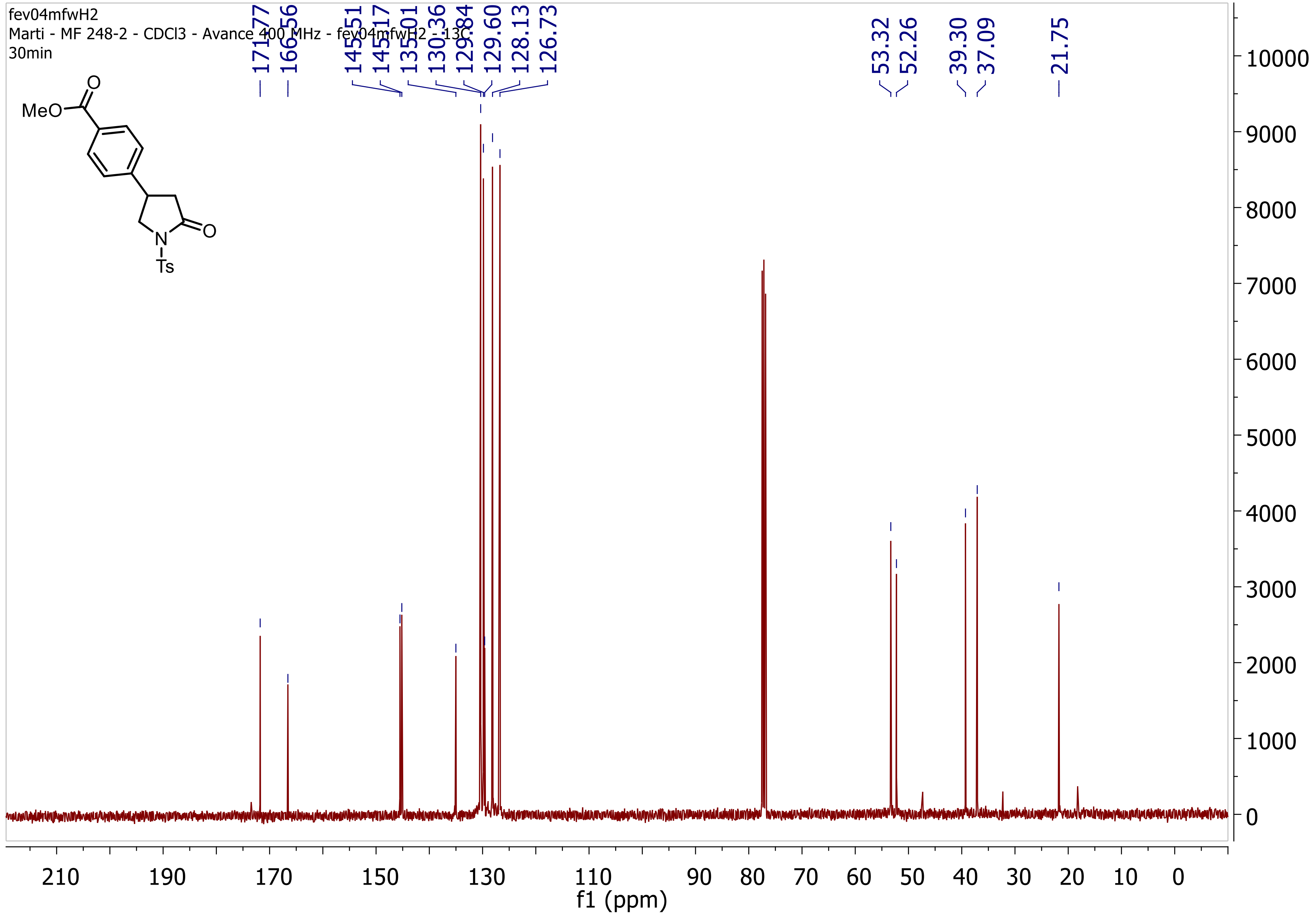

Figure SI21: ^{13}C NMR of compound **4bd**.

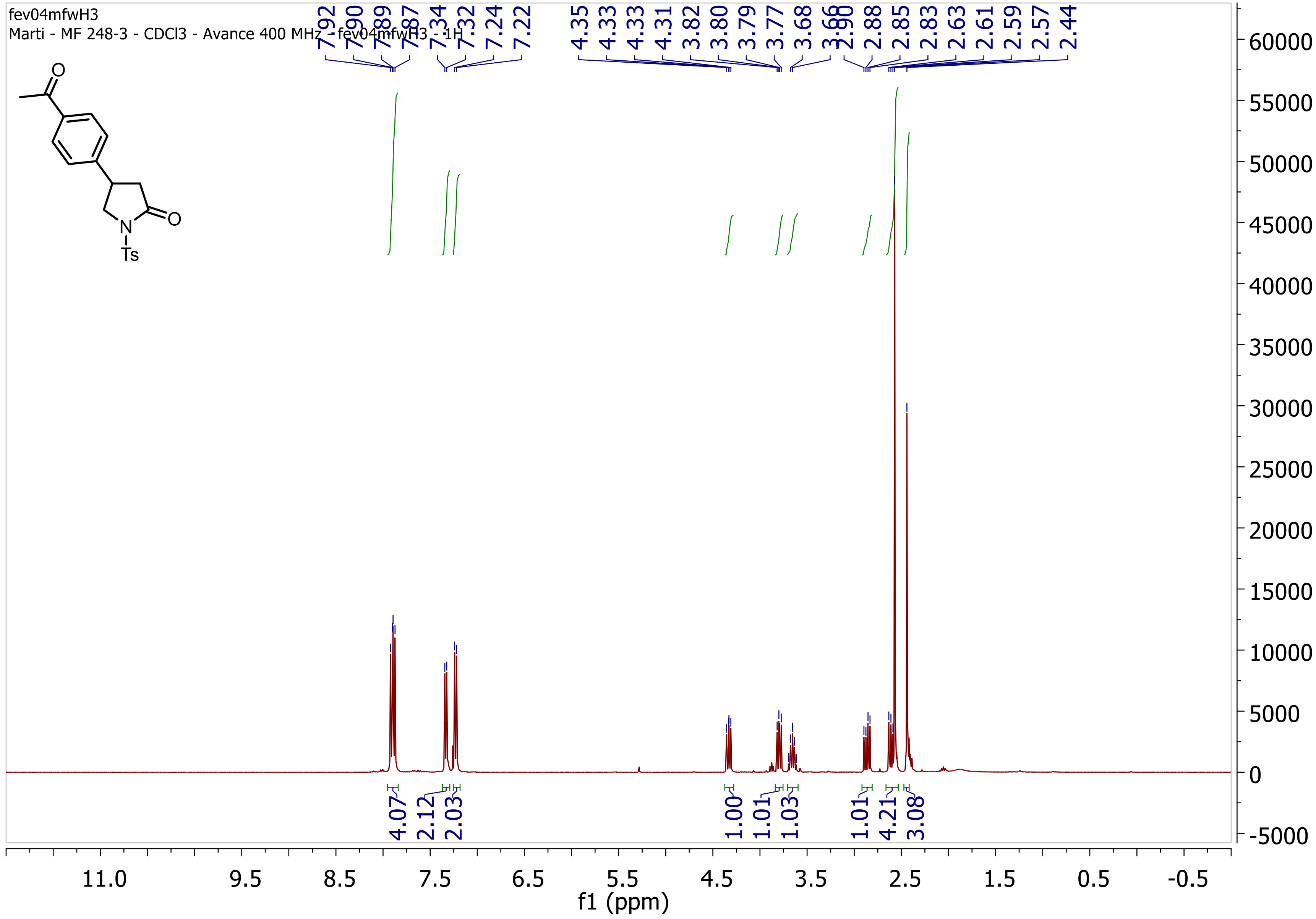

Figure SI22: ^{1}H NMR of compound **4be**.

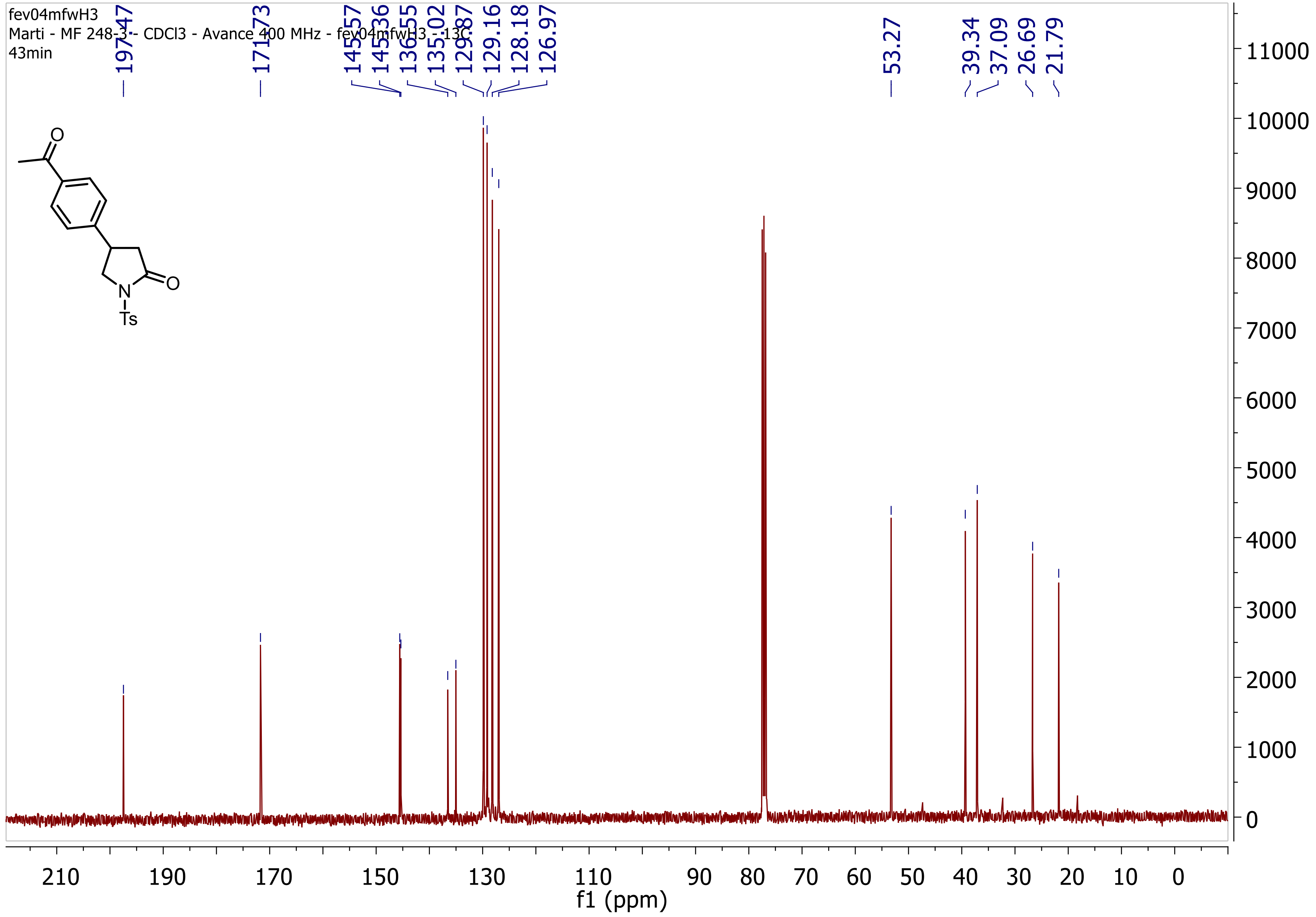

Figure SI23: ^{13}C NMR of compound **4be**.

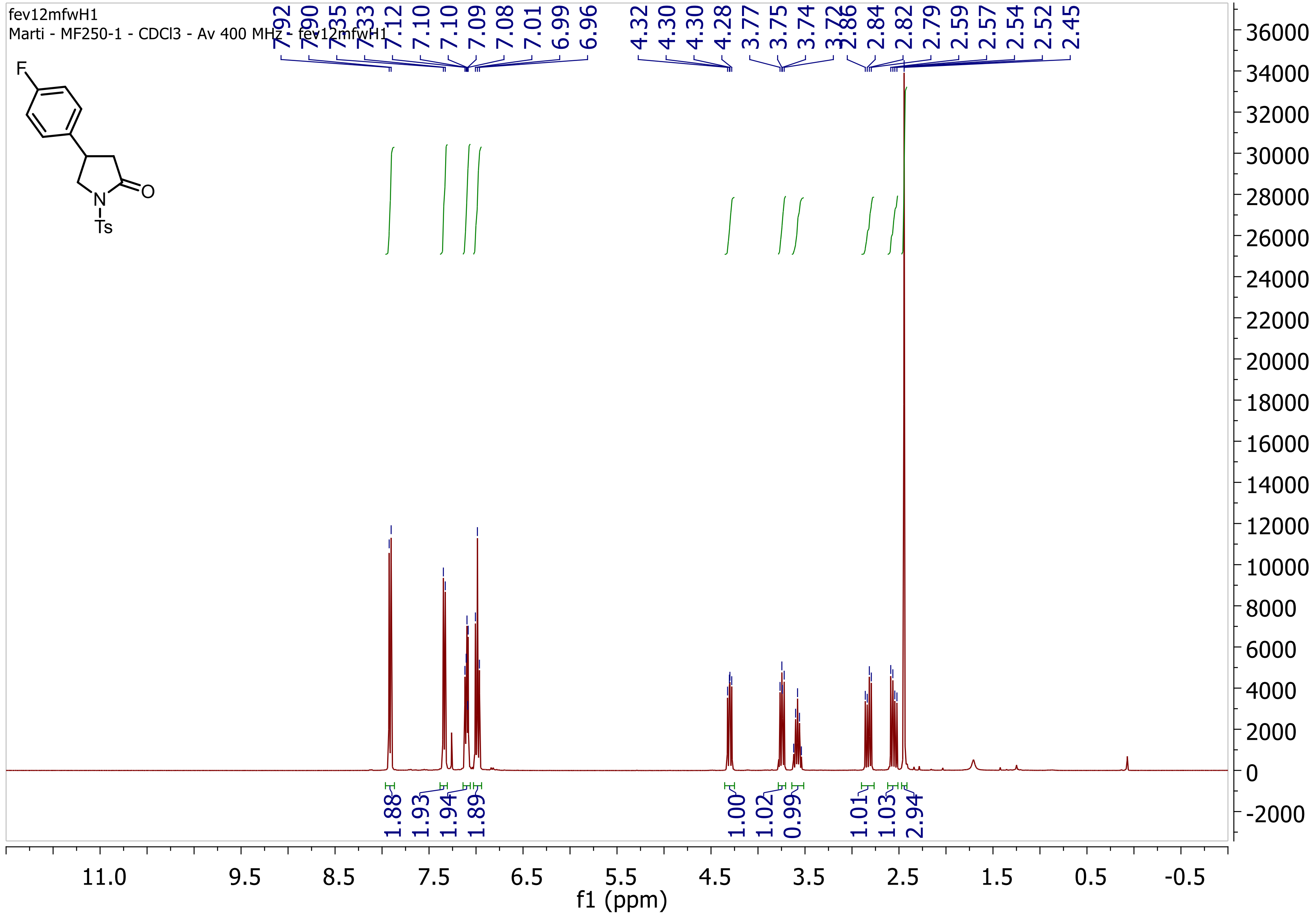

Figure SI24: ^{1}H NMR of compound **4bf**.

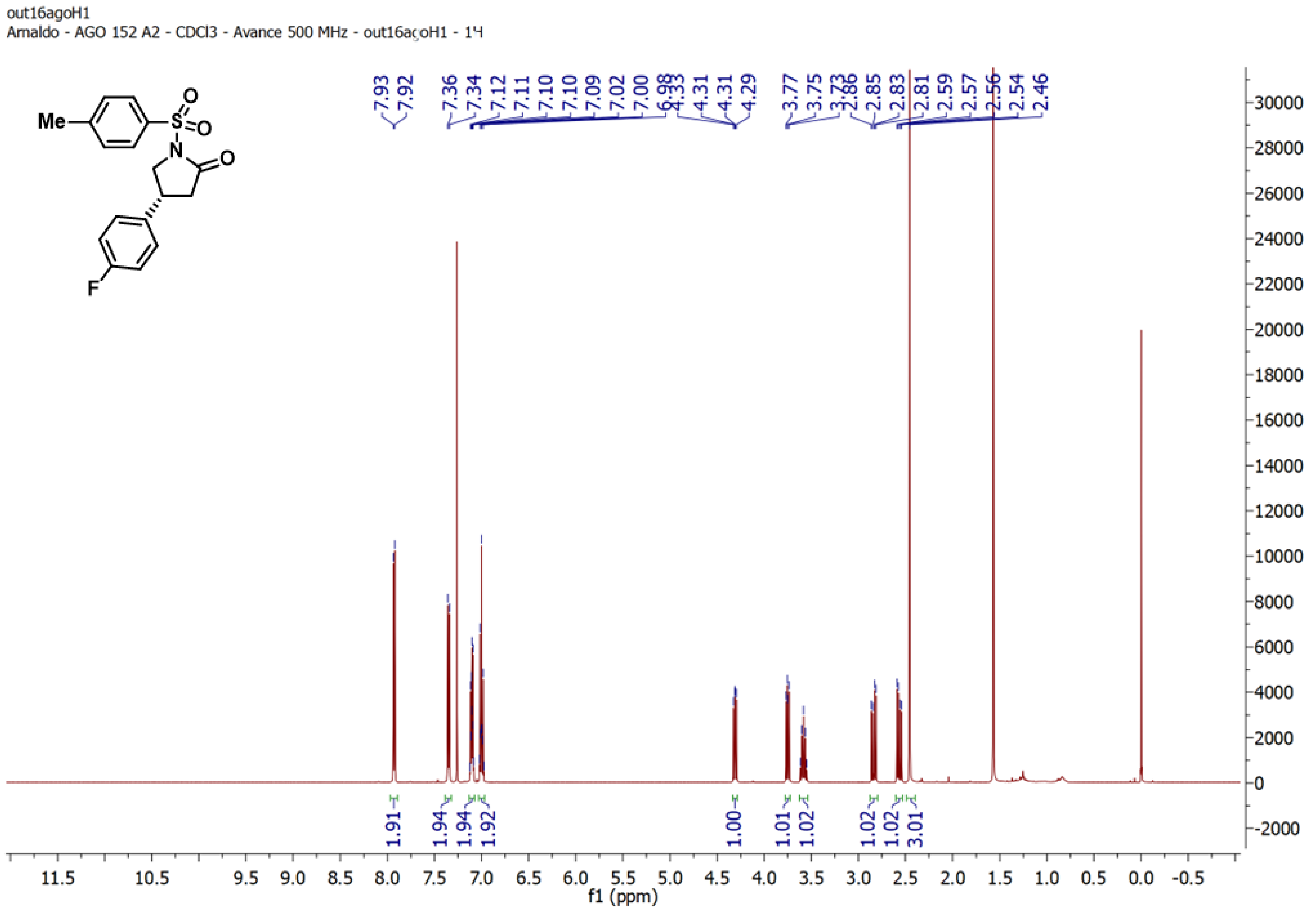

Figure SI25: ^{1}H NMR of compound **4bf**.

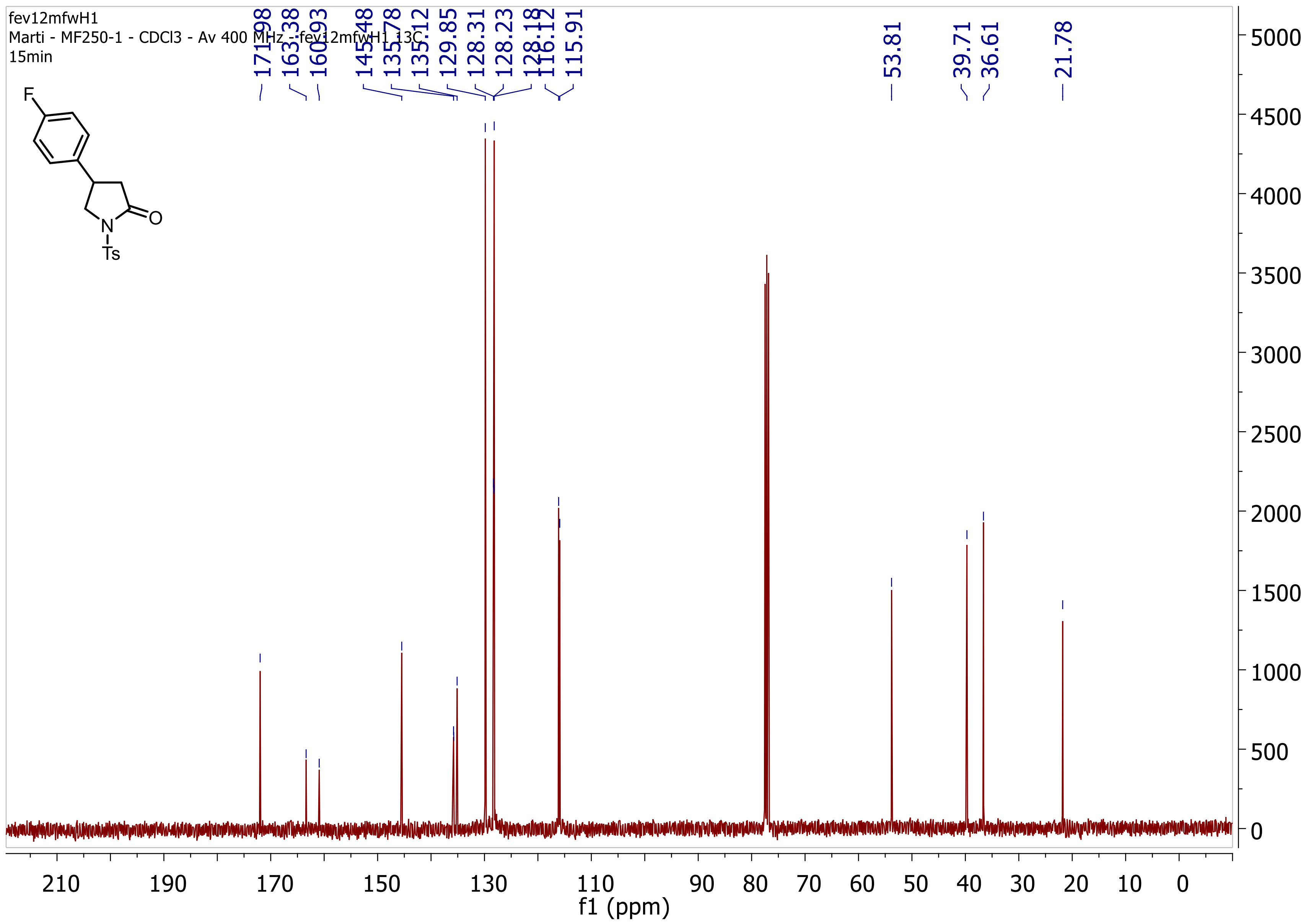

Figure SI26: ^{13}C NMR of compound **4bf**.

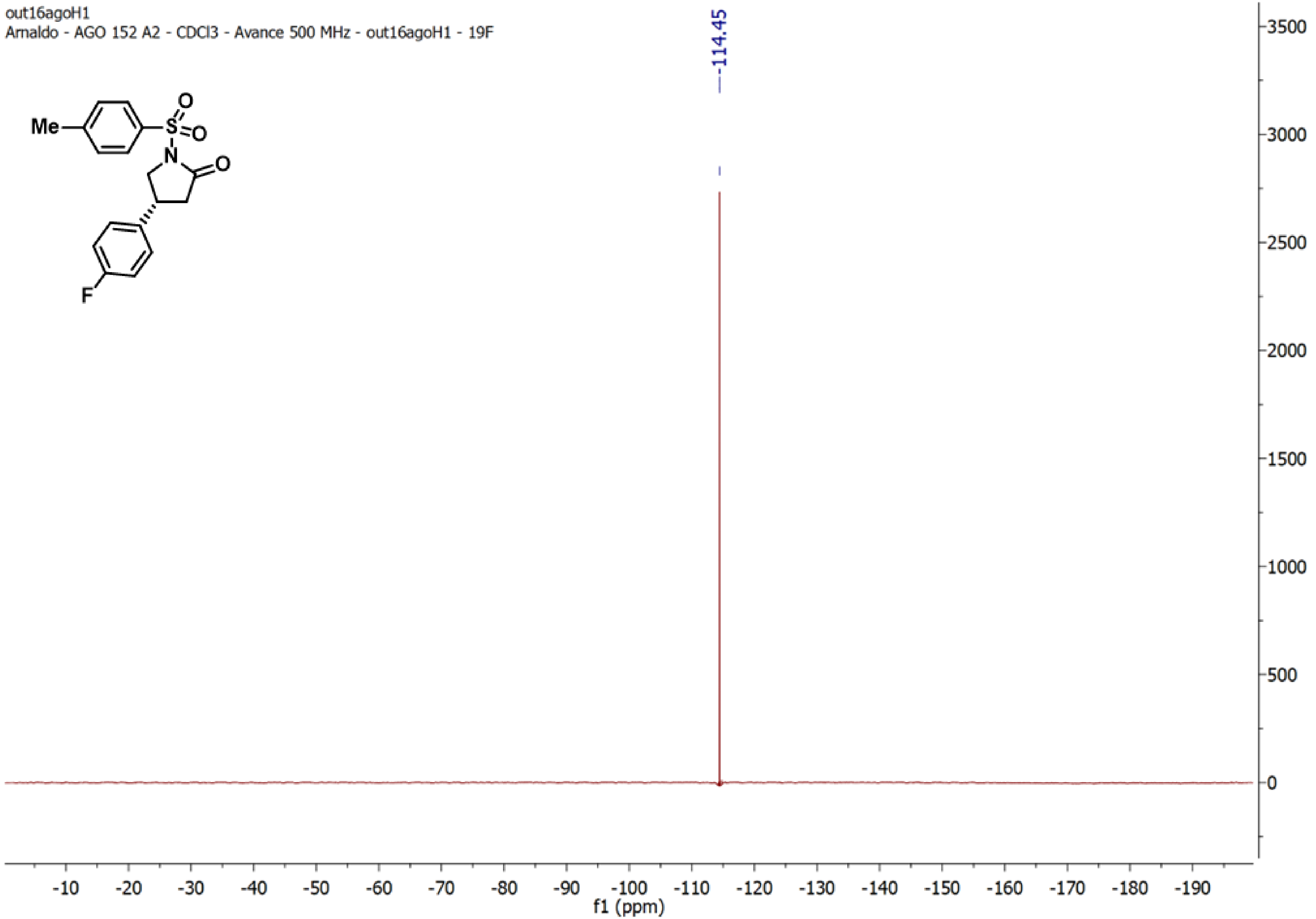

Figure SI27: ^{19}F NMR of compound **4bf**.

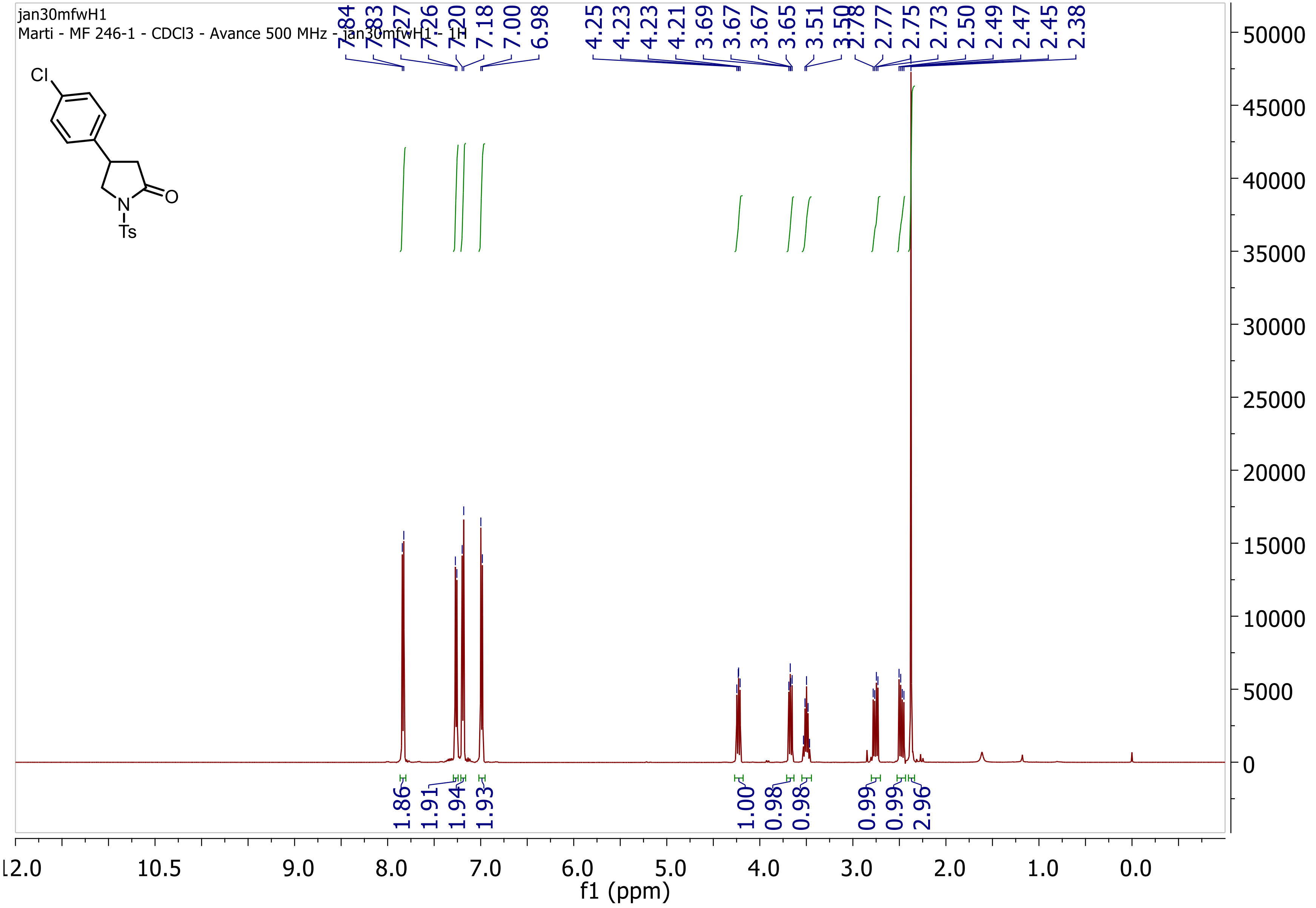

Figure SI28: ^{1}H NMR of compound **4bg**.

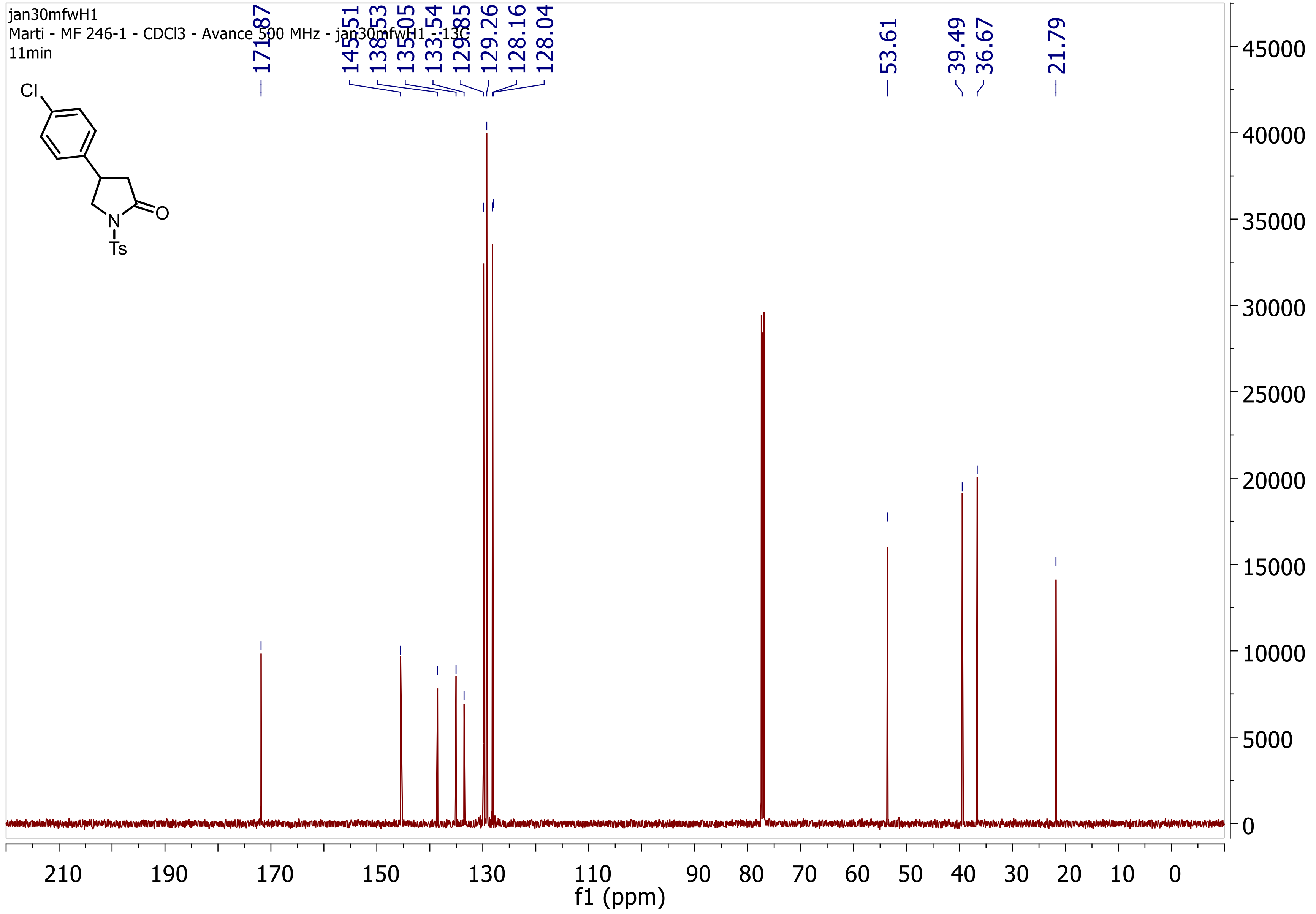

Figure SI29: ^{13}C NMR of compound **4bg**

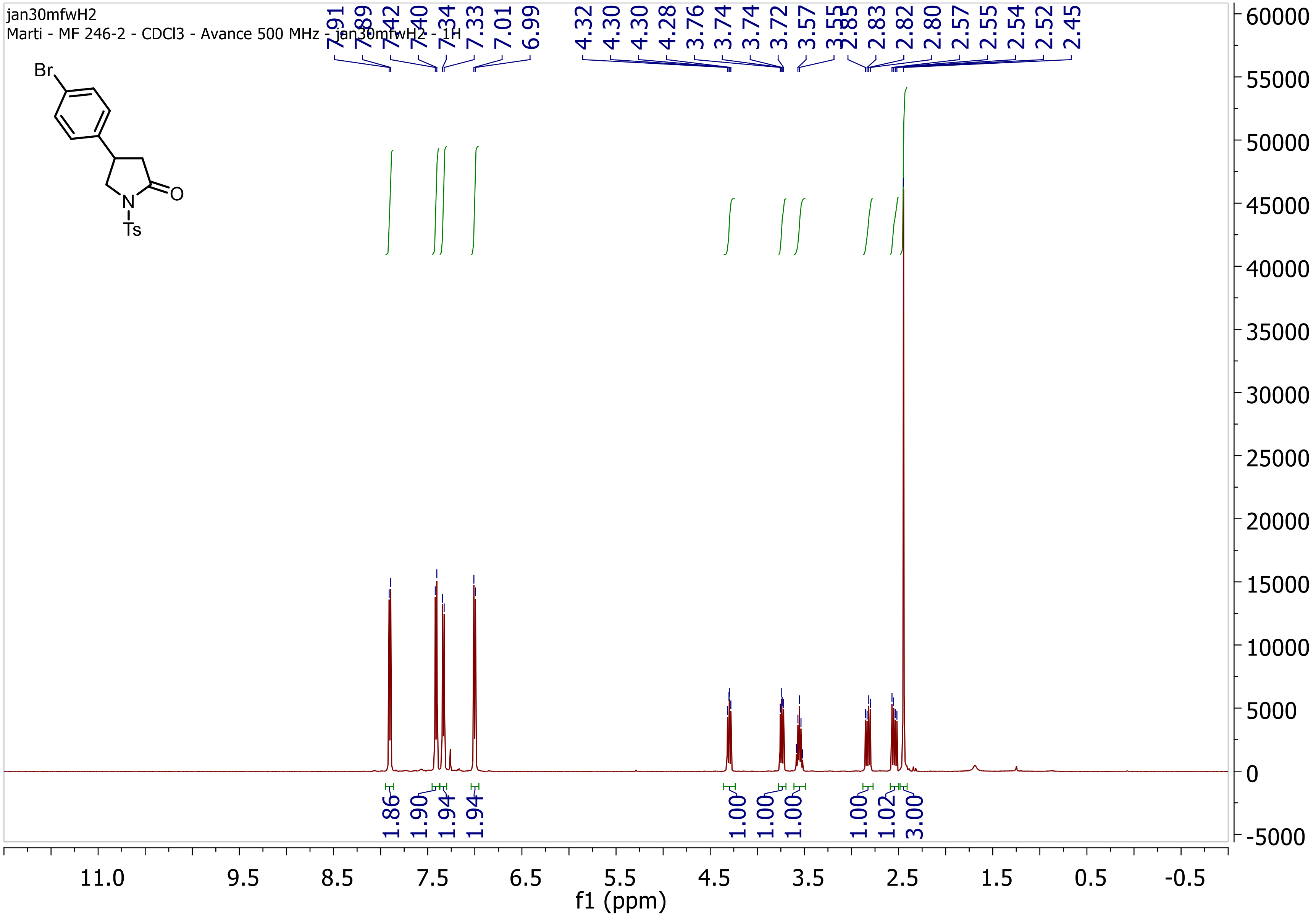

Figure SI30: ^{1}H NMR of compound **4bh**.

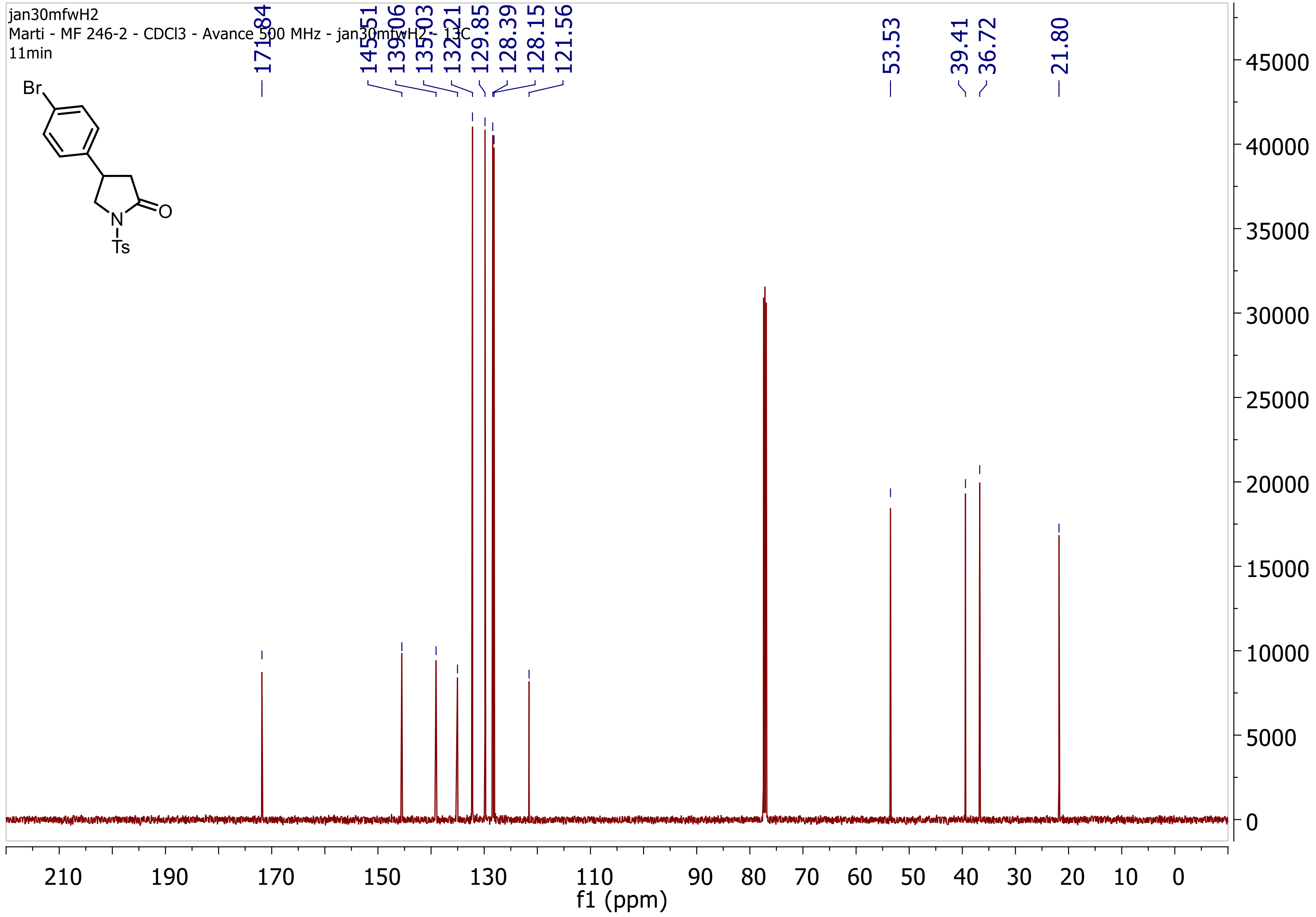

Figure SI31: ^{13}C NMR of compound **4bh**.

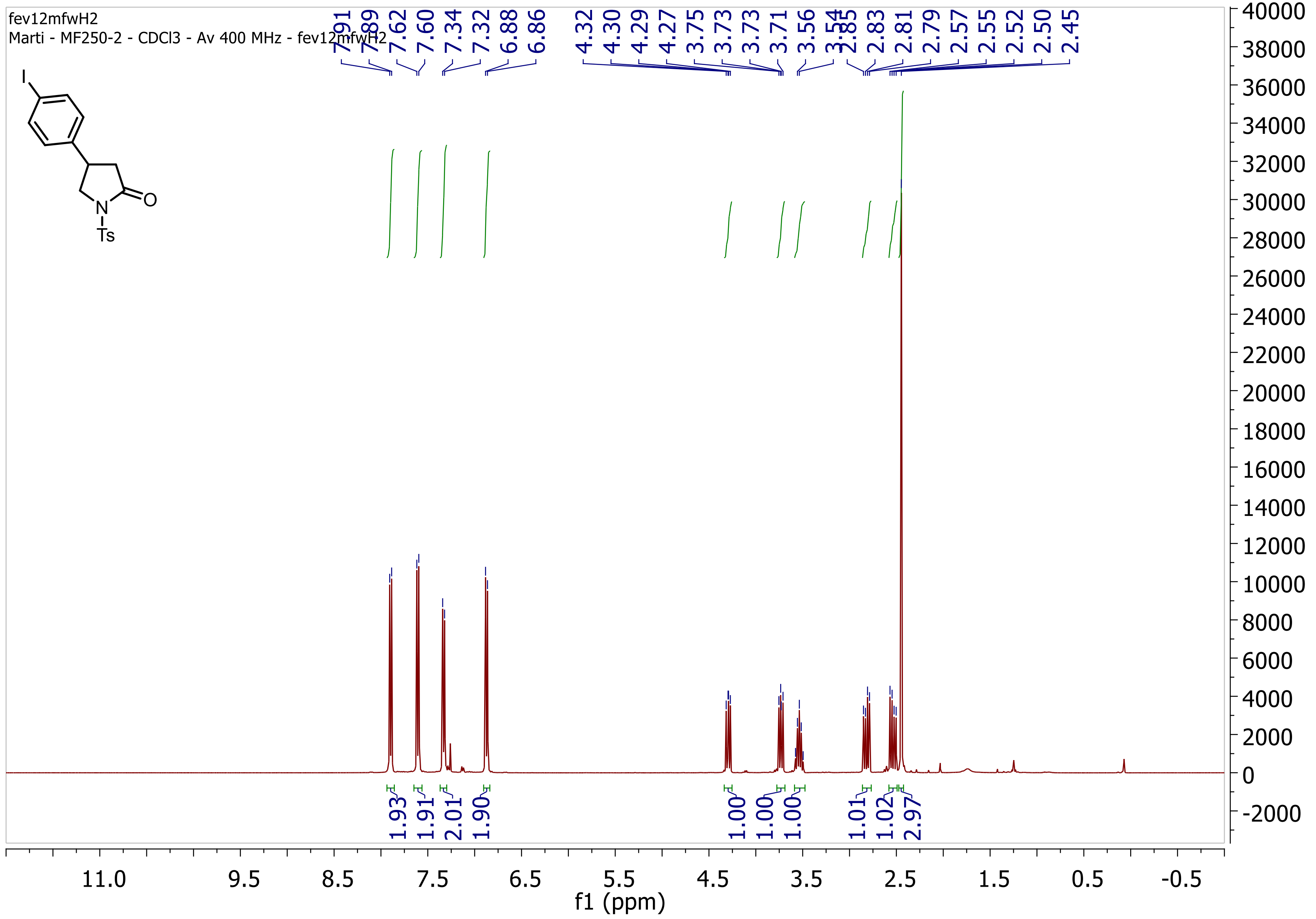

Figure SI32: ^{1}H NMR of compound **4bi**.

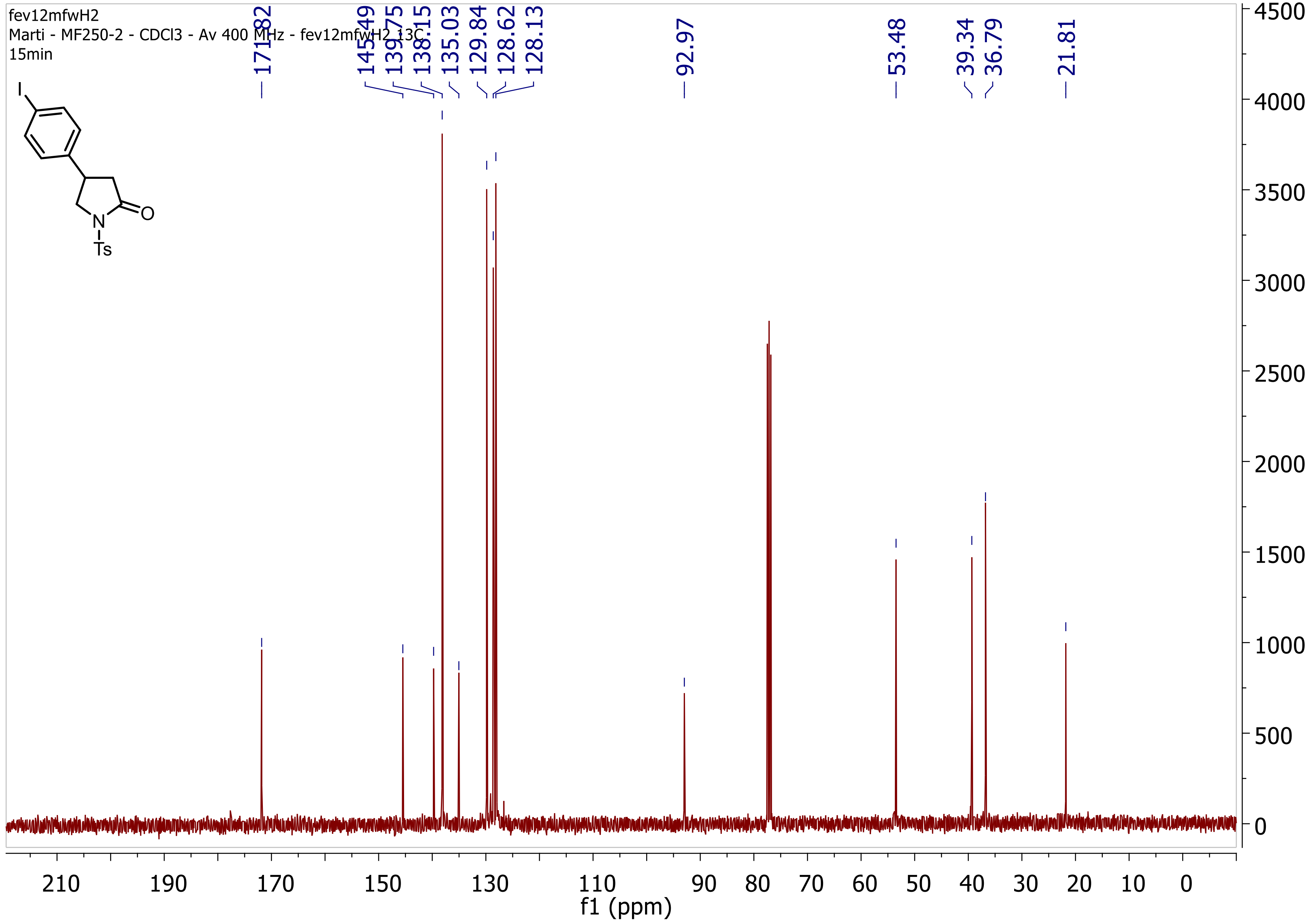

Figure SI33: ^{13}C NMR of compound **4bi**.

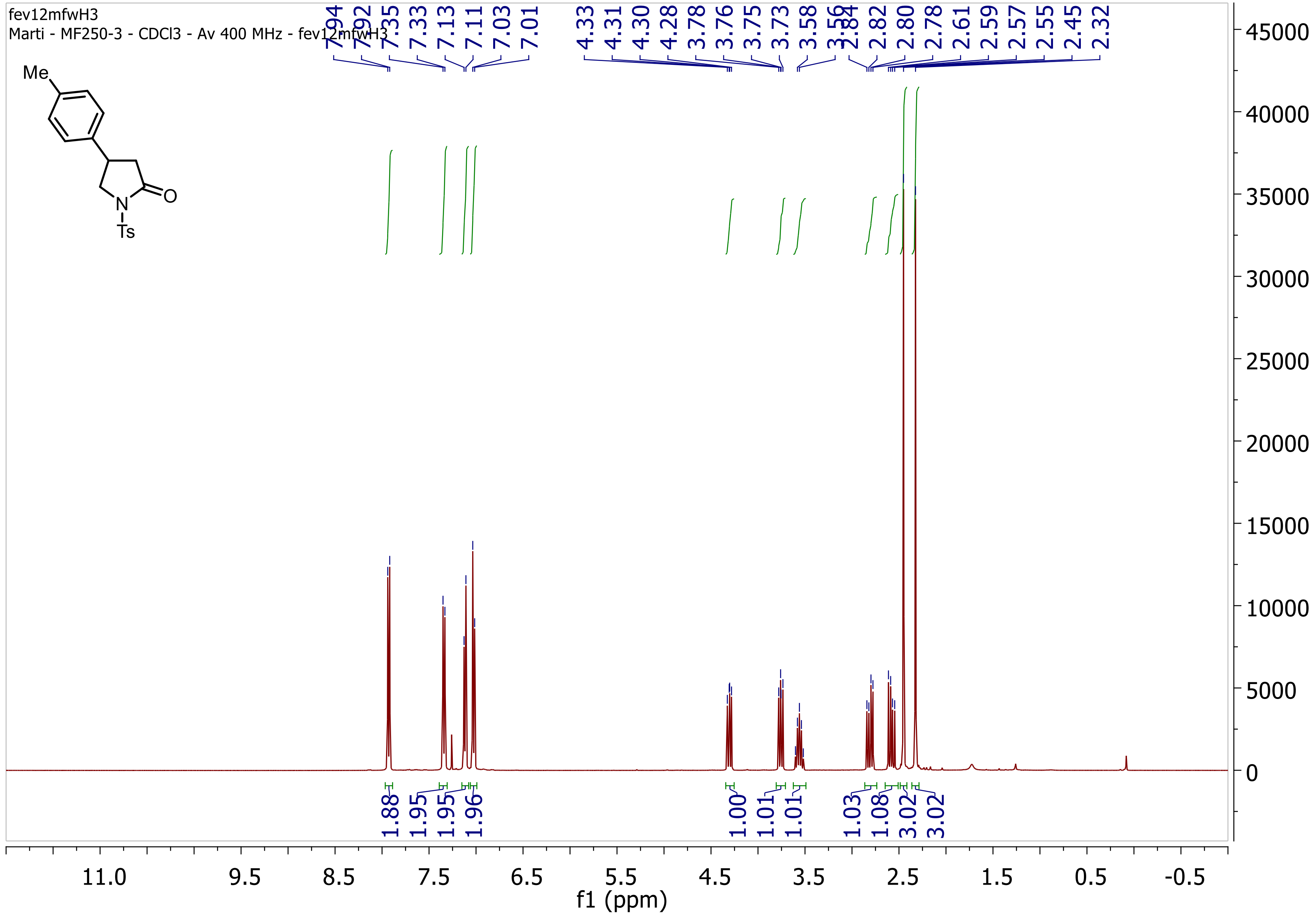

Figure SI34: ^{1}H NMR of compound **4bj**.

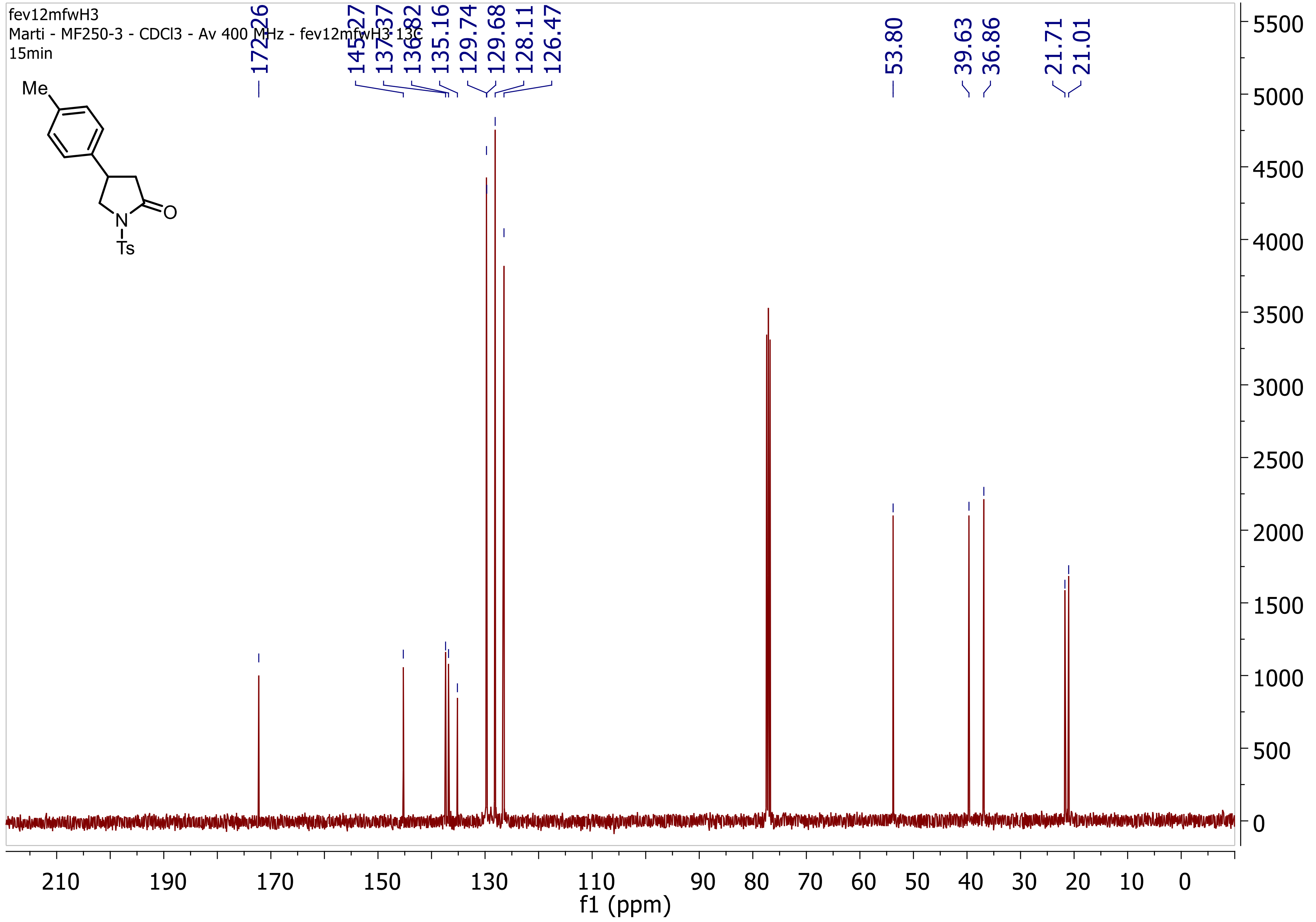

Figure SI35: ^{13}C NMR of compound **4bi**.

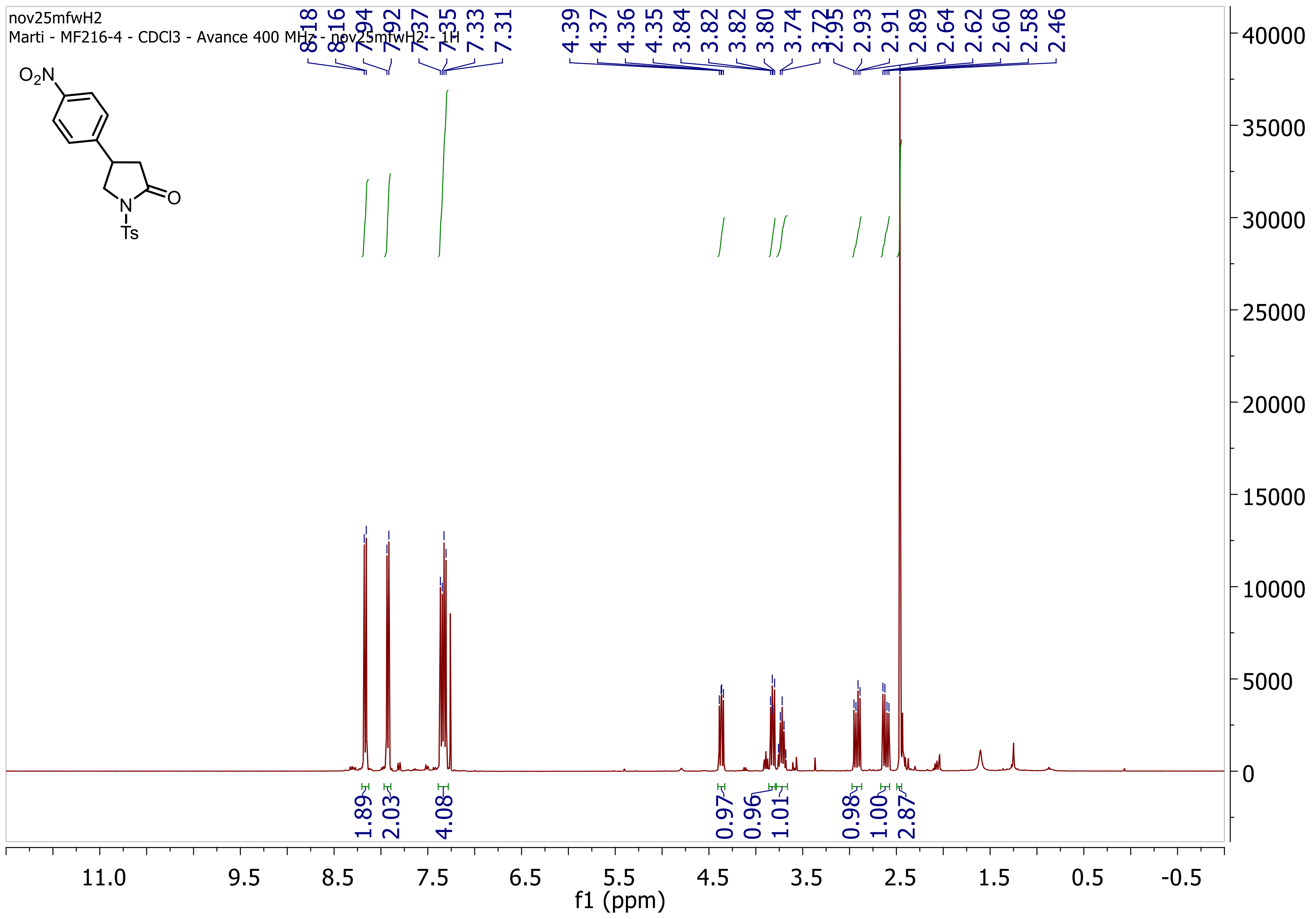

Figure SI36: ^{1}H NMR of compound **4bk**.

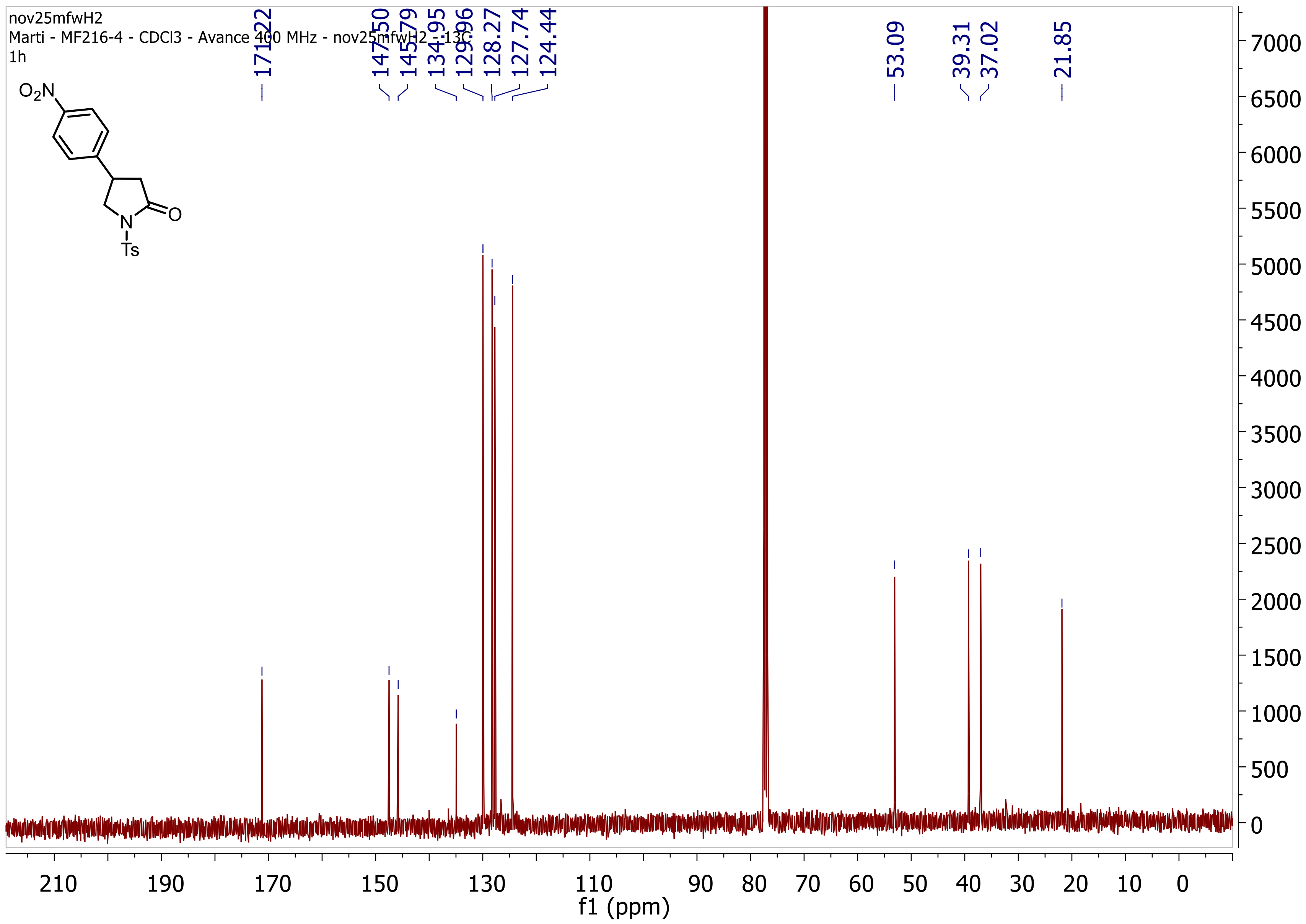

Figure SI37: ^{13}C NMR of compound **4bk**.

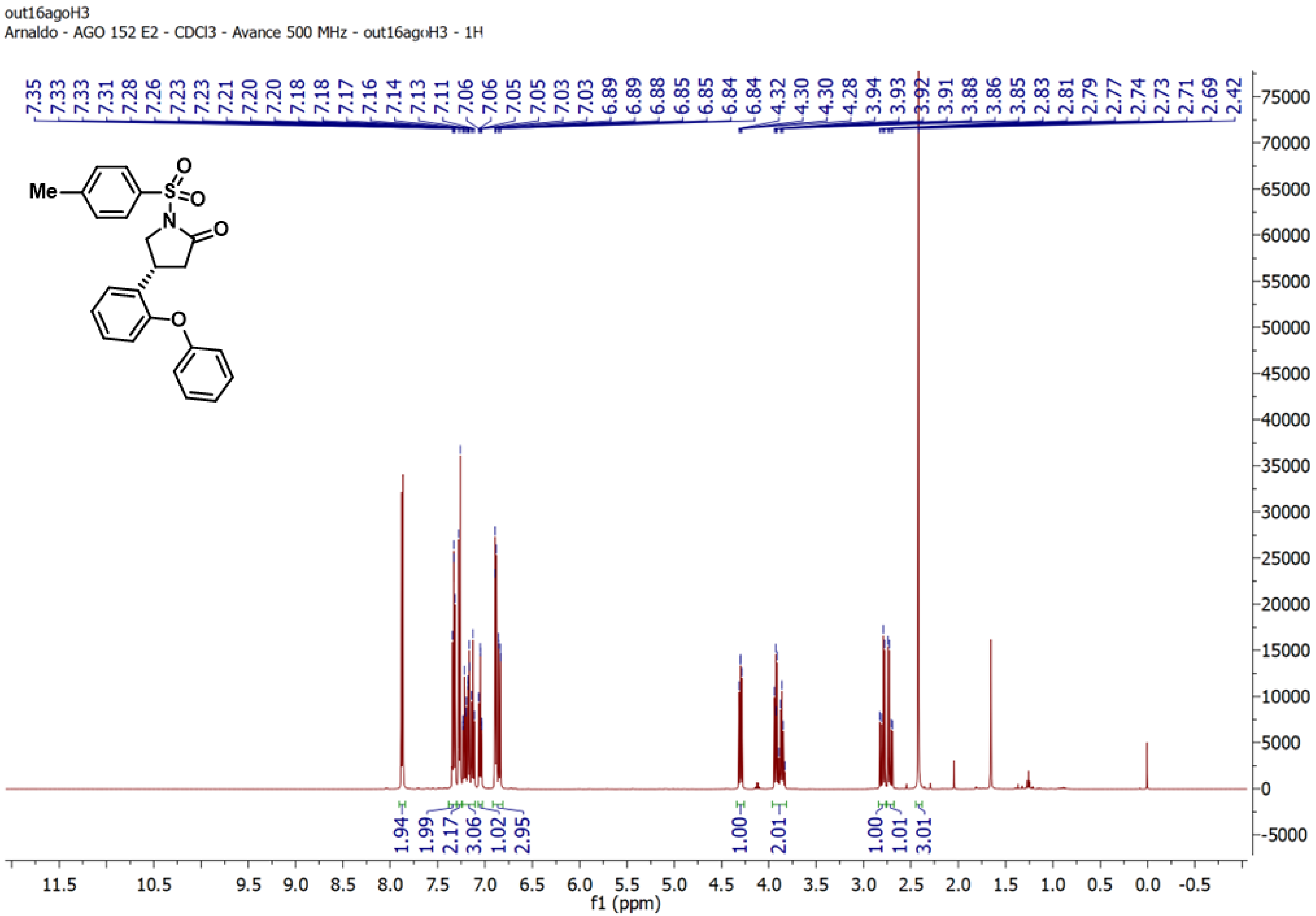

Figure SI38: ^{1}H NMR of compound **4bl**.

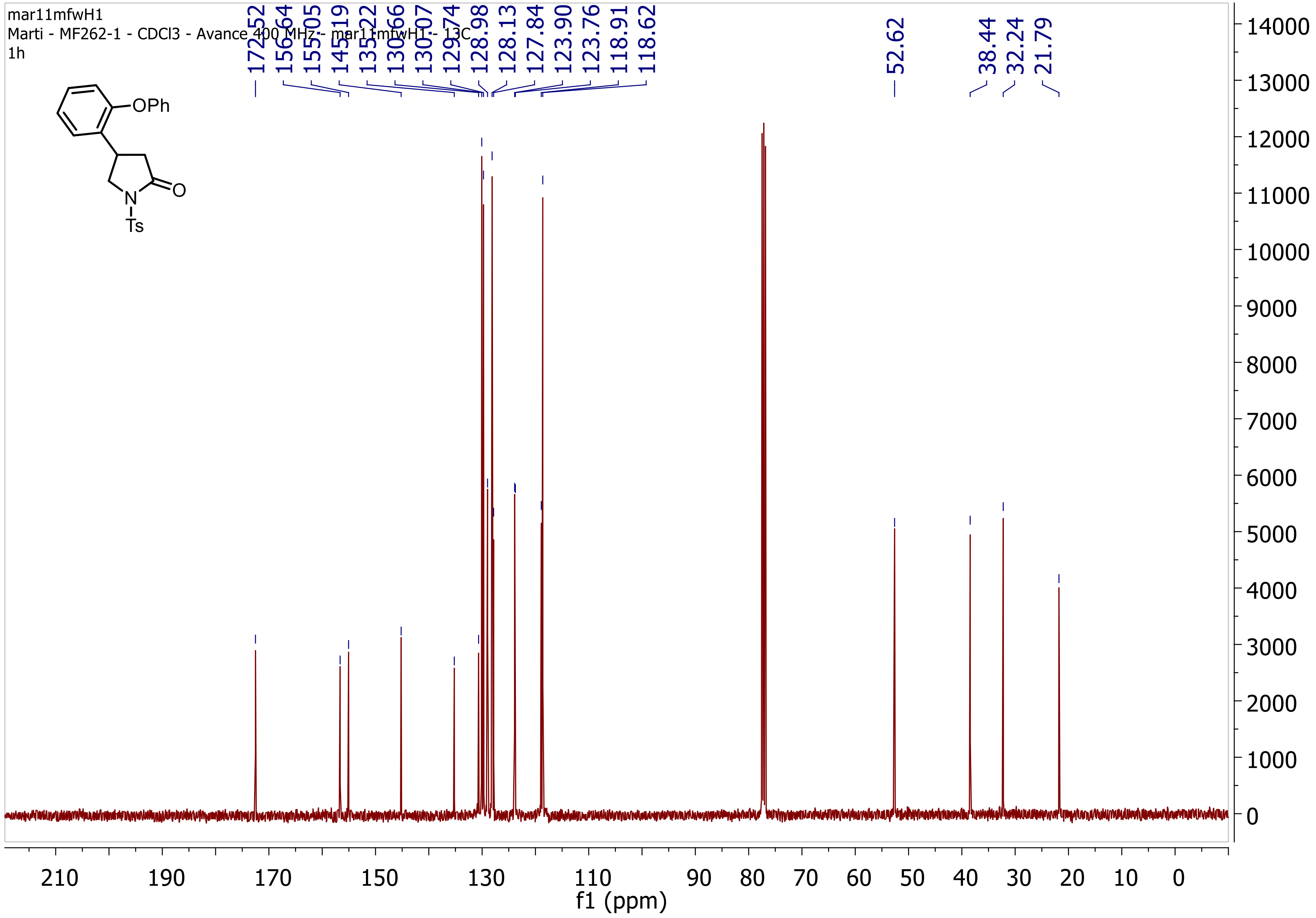

Figure SI39: ^{13}C NMR of compound **4bl**.

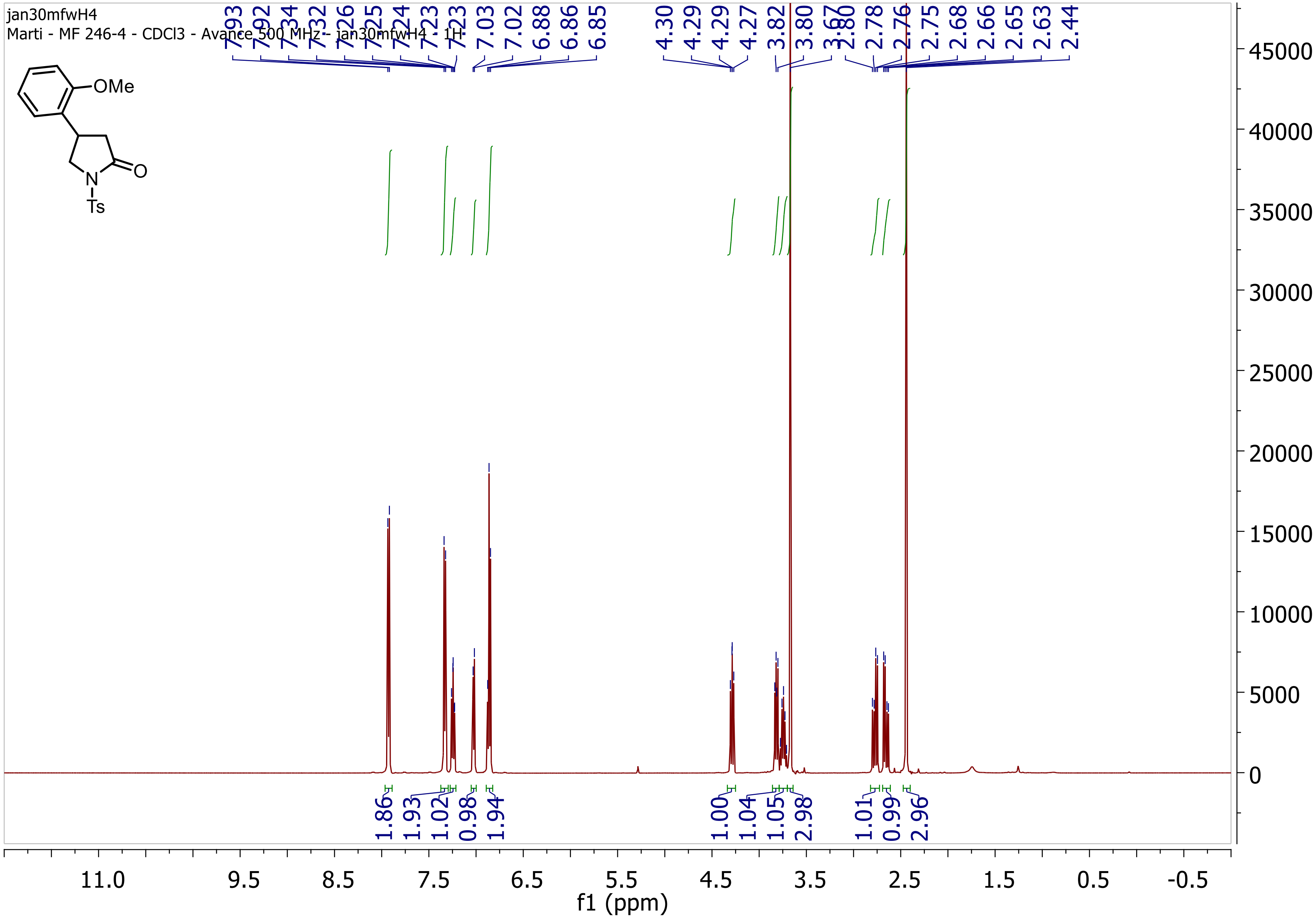

Figure SI40: ^{1}H NMR of compound **4bm**.

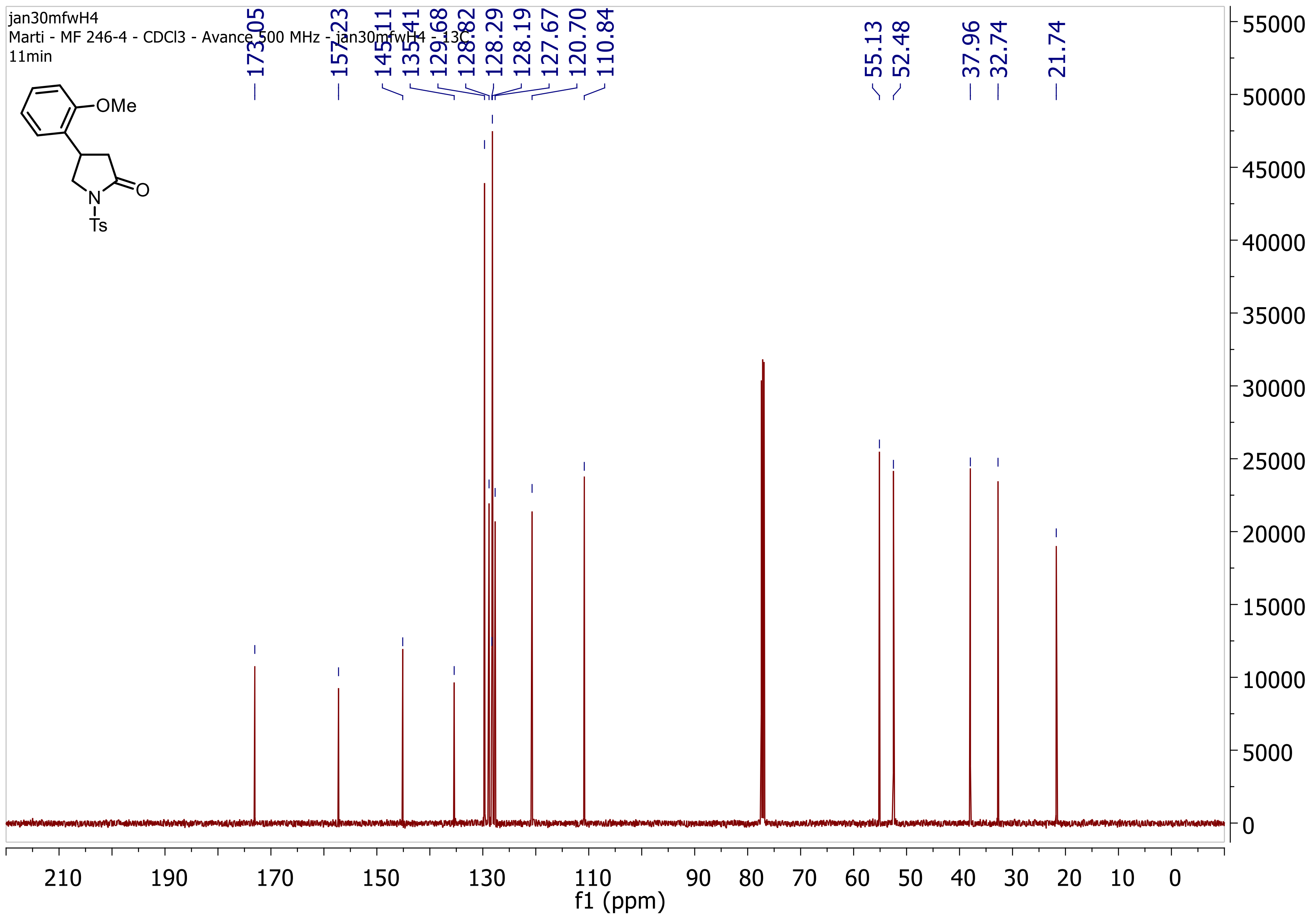

Figure SI41: ^{13}C NMR of compound **4bm**.

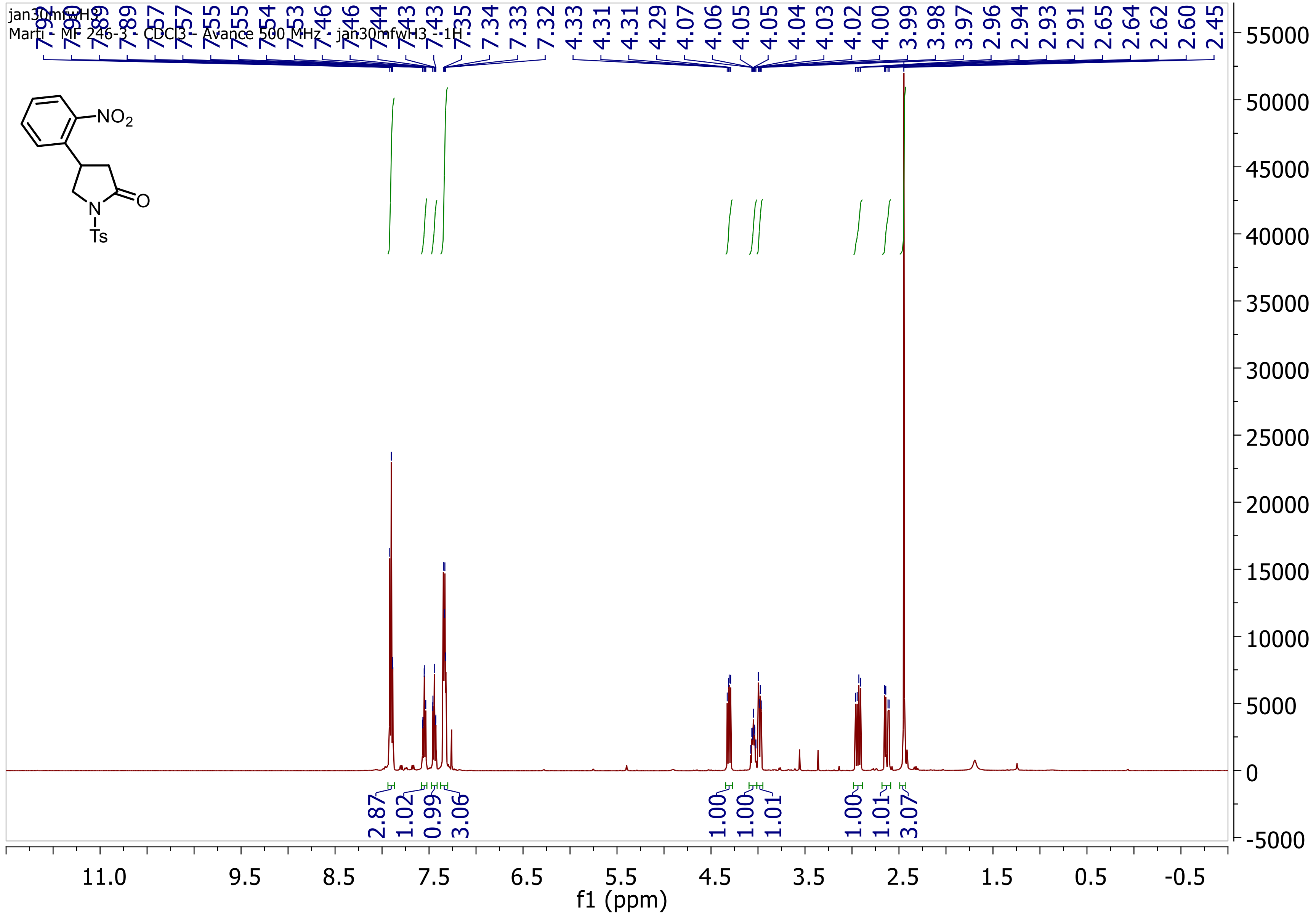

Figure SI42: ^{1}H NMR of compound **4bn**.

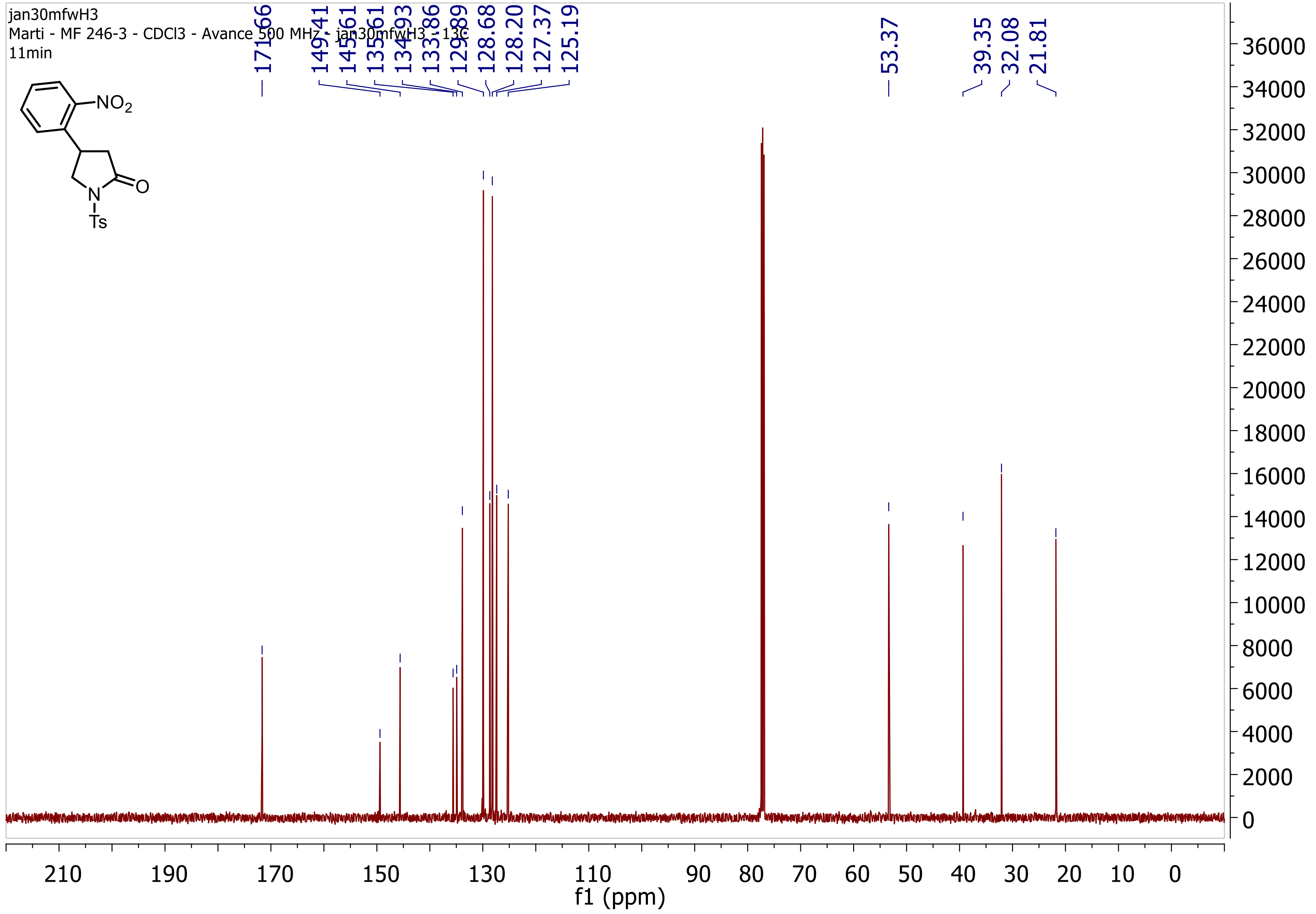

Figure SI43: ^{13}C NMR of compound **4bn**.

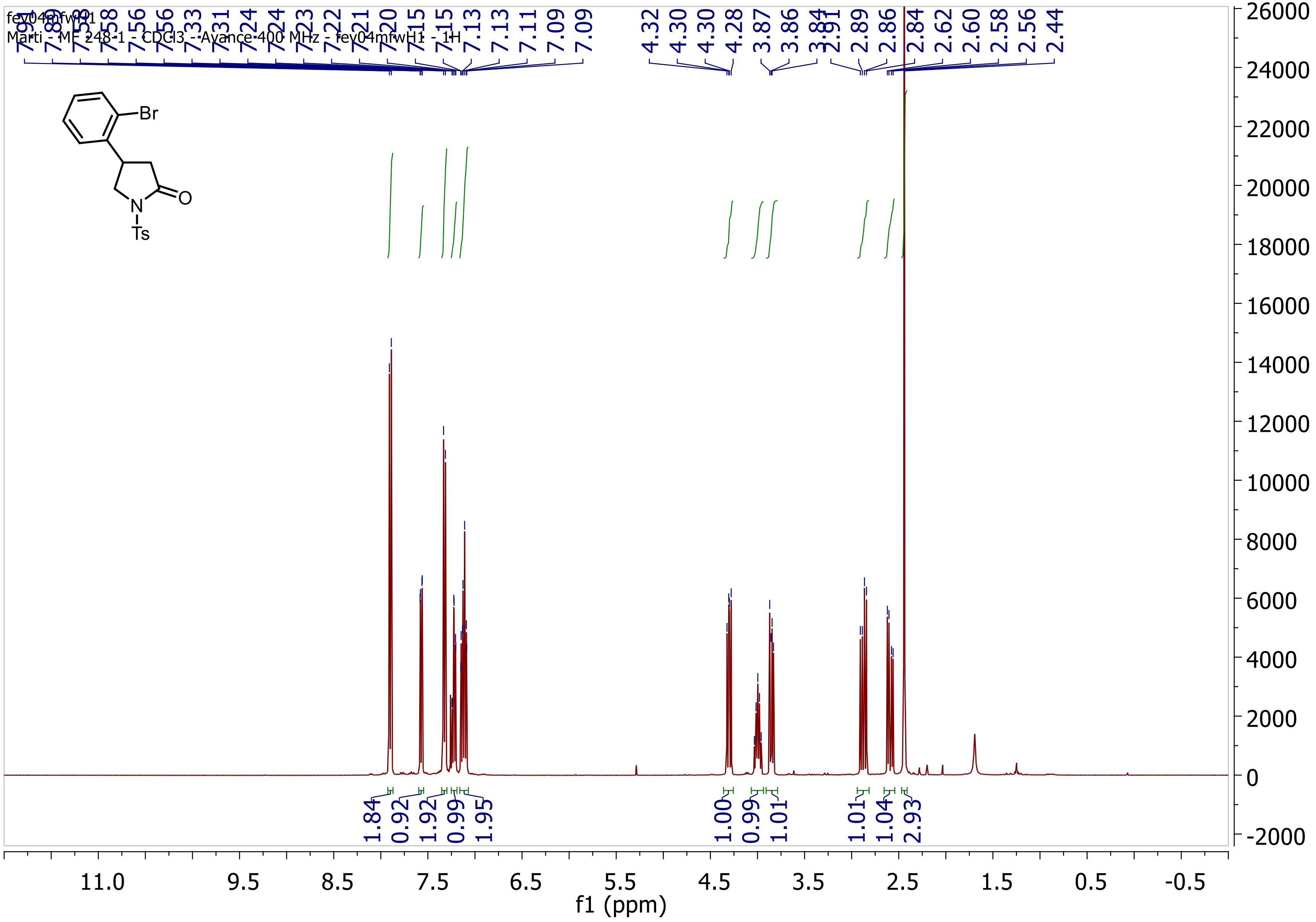

Figure SI44: ^{1}H NMR of compound **4bo**.

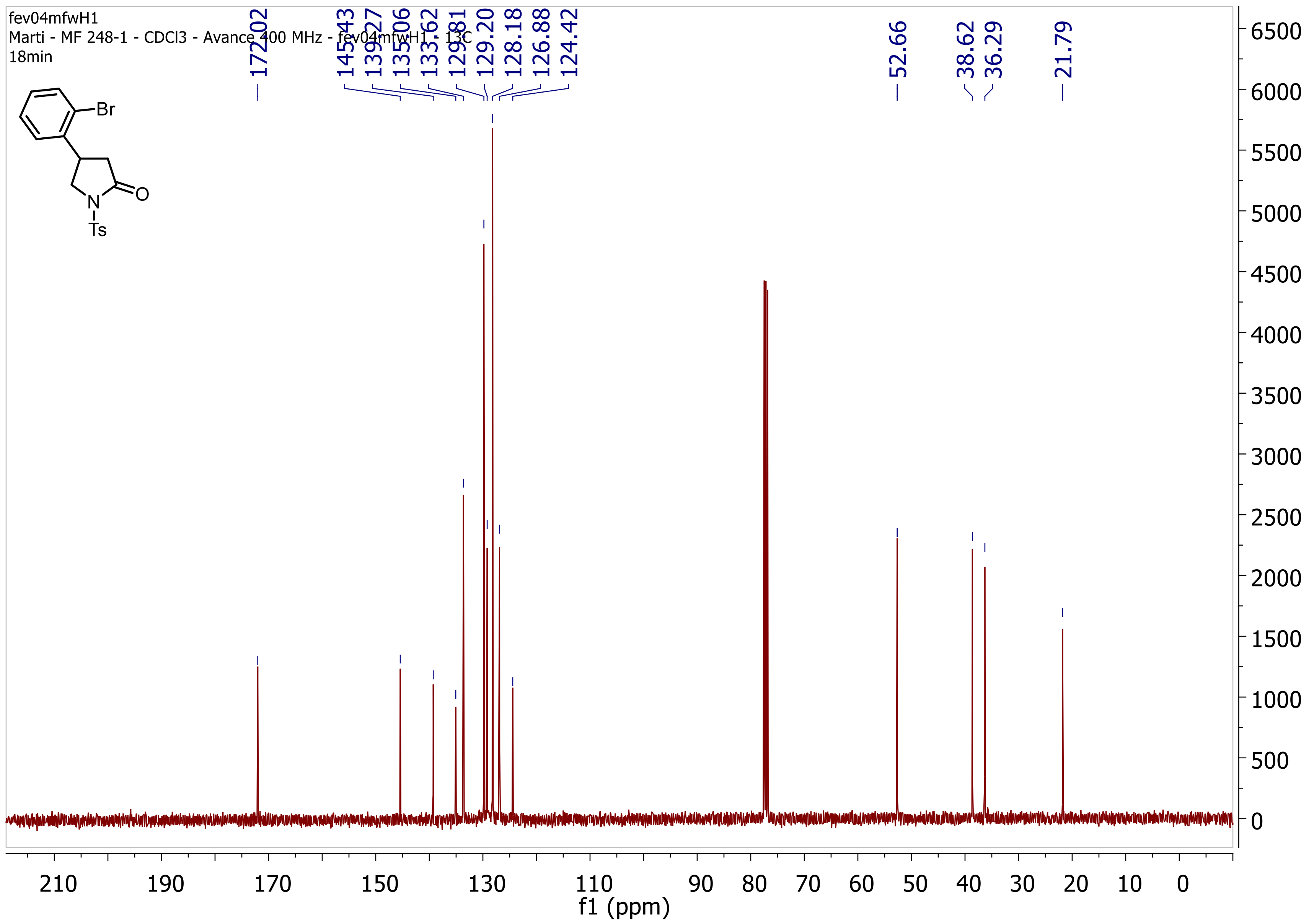

Figure SI45: ^{13}C NMR of compound **4bo**.

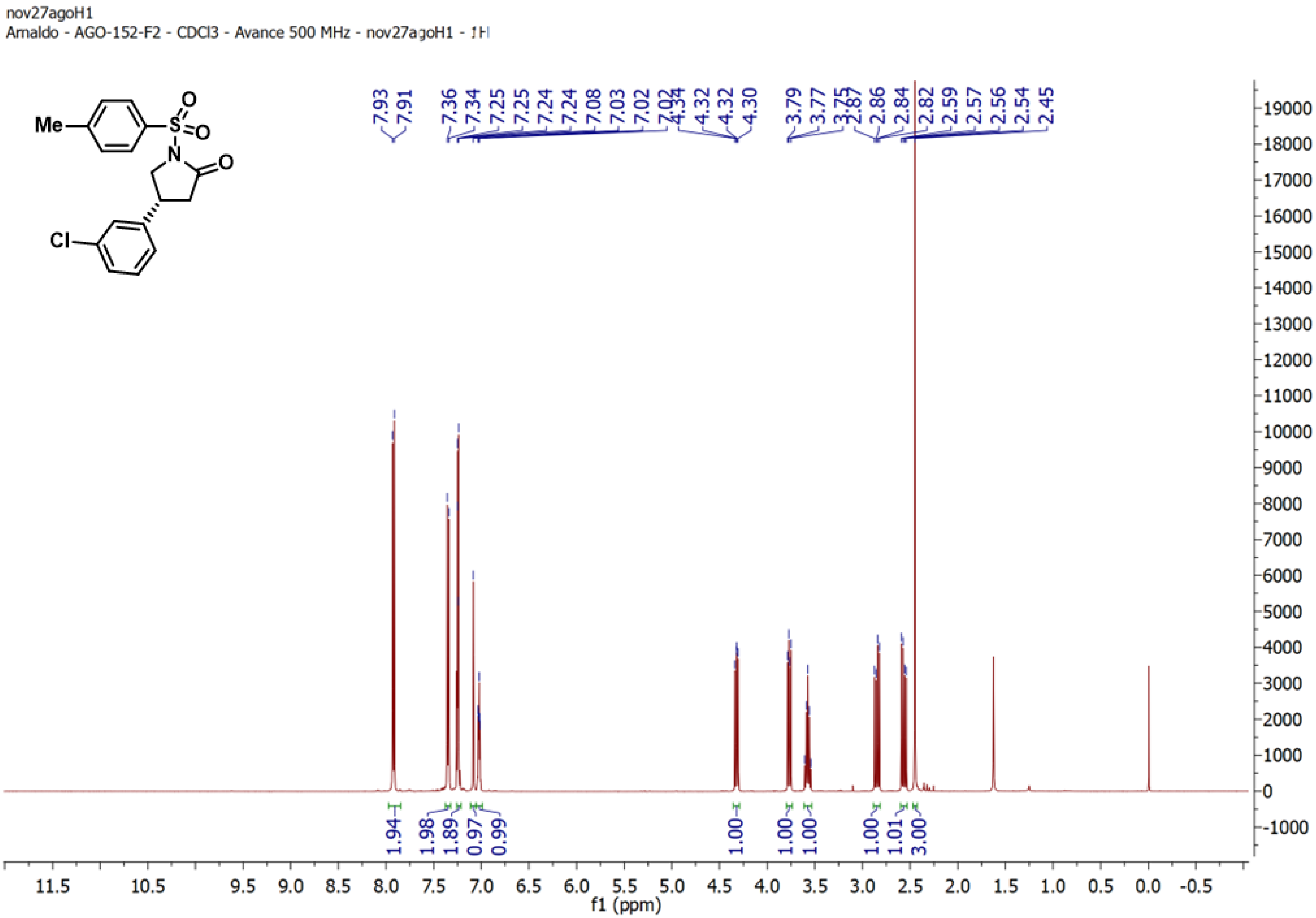

Figure SI46: 1H NMR of compound **4bp**

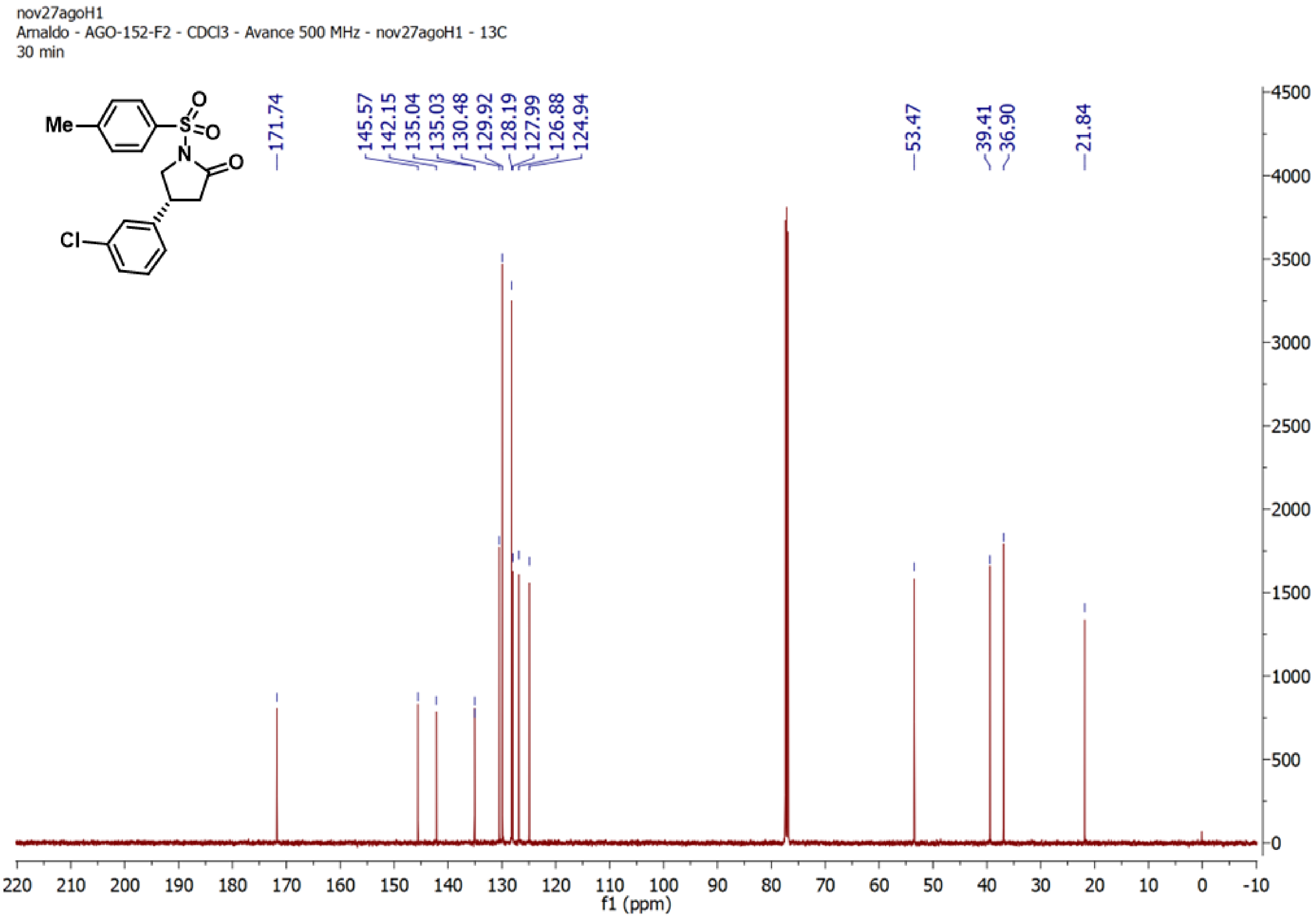

Figure SI47: ^{13}C NMR of compound **4bp.**

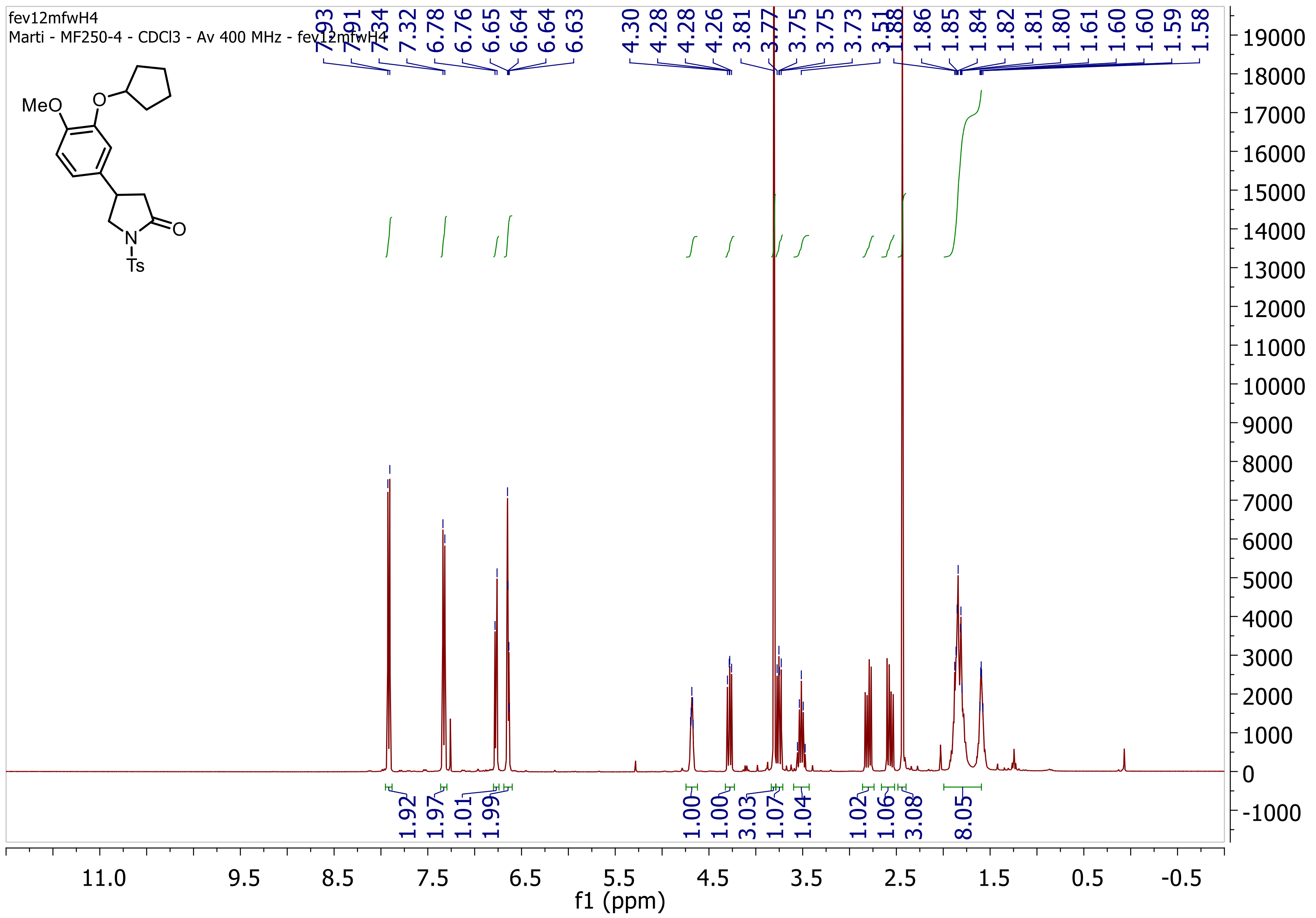

Figure SI48: ^{1}H NMR of compound **4bq**.

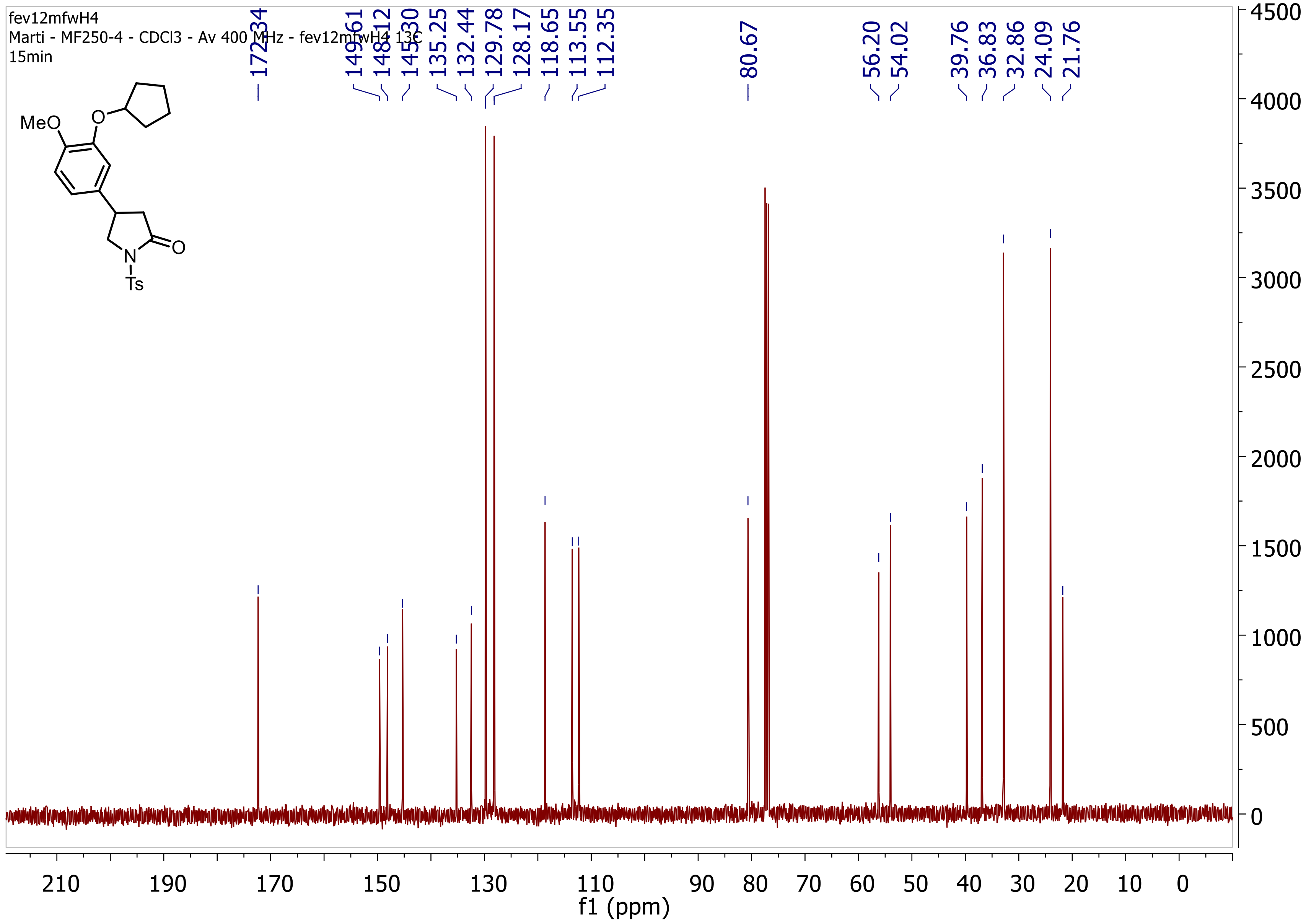

Figure SI49: ^{13}C NMR of compound **4bq**.

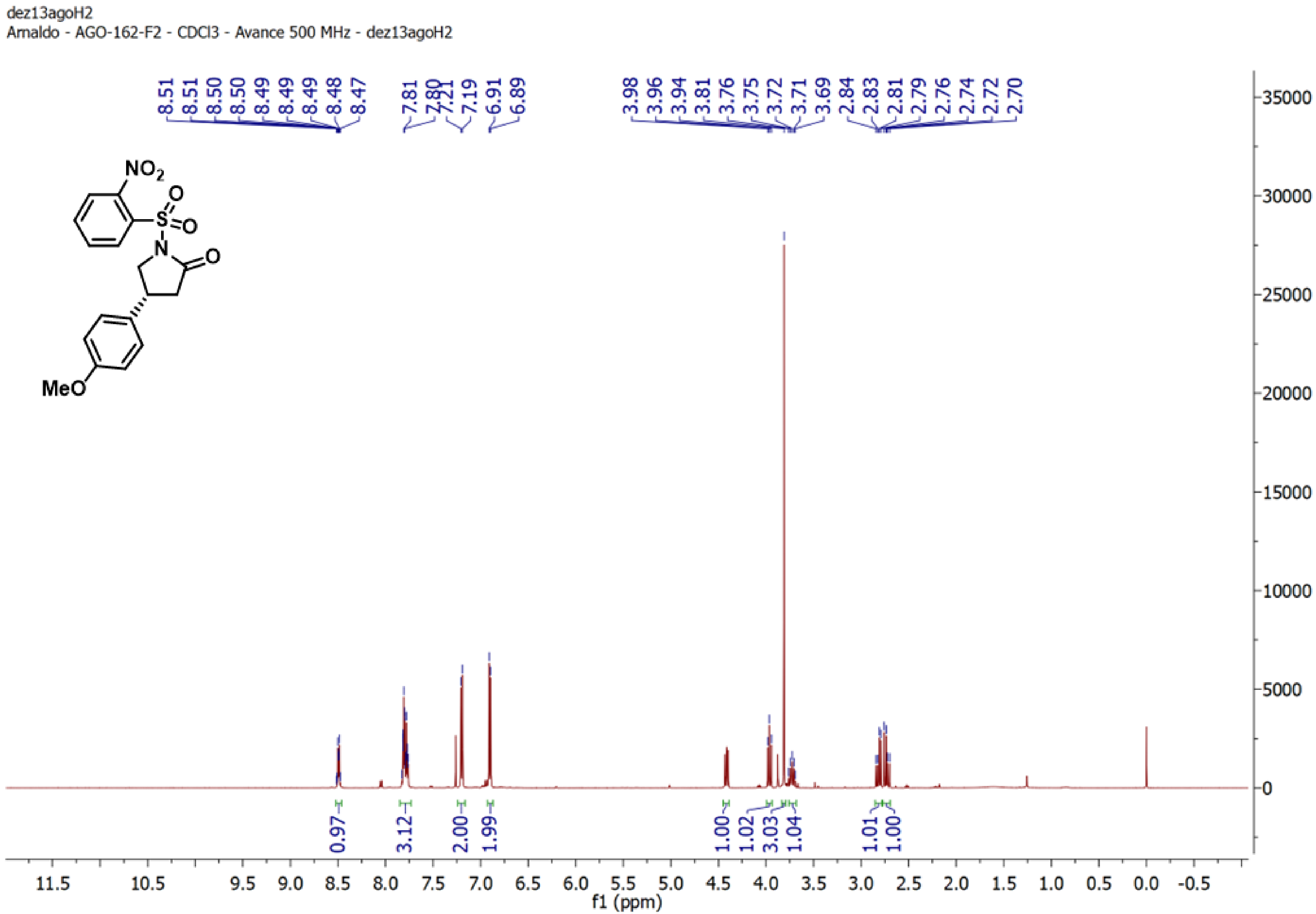

Figure SI50: ^{1}H NMR of compound **4ca**.

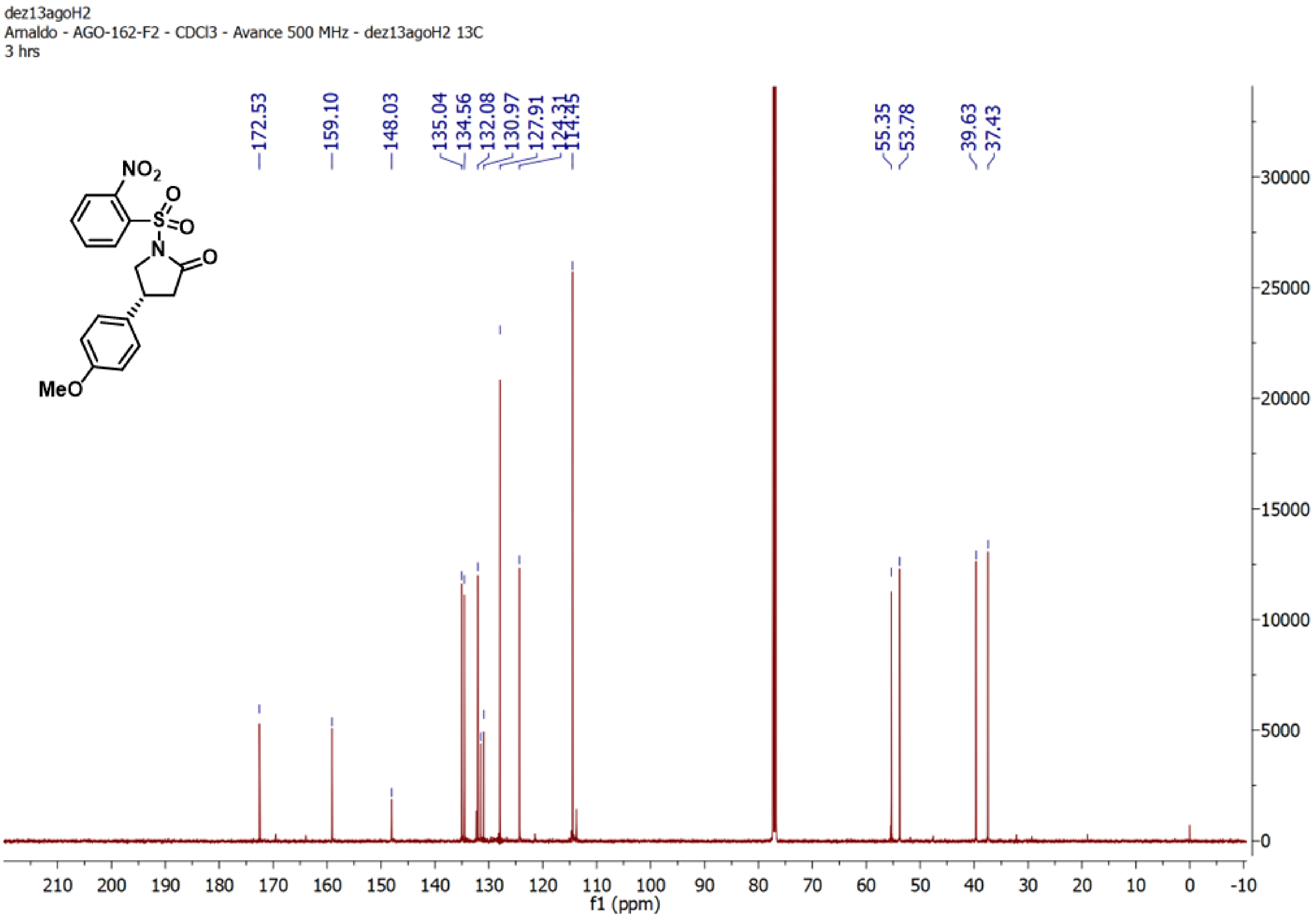

Figure SI51: ^{13}C NMR of compound **4ca**.

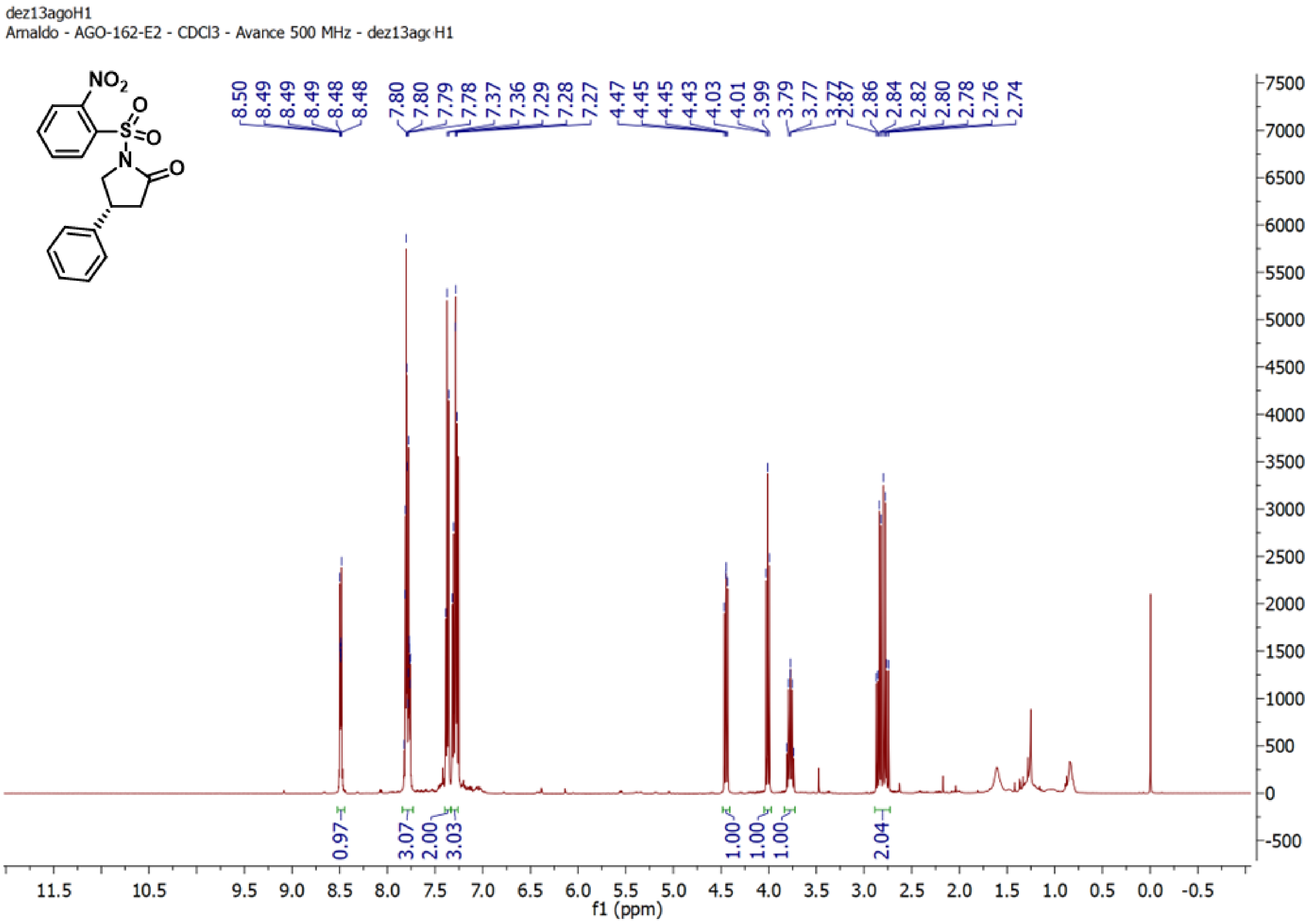

Figure SI52: ^{1}H NMR of compound **4cb**.

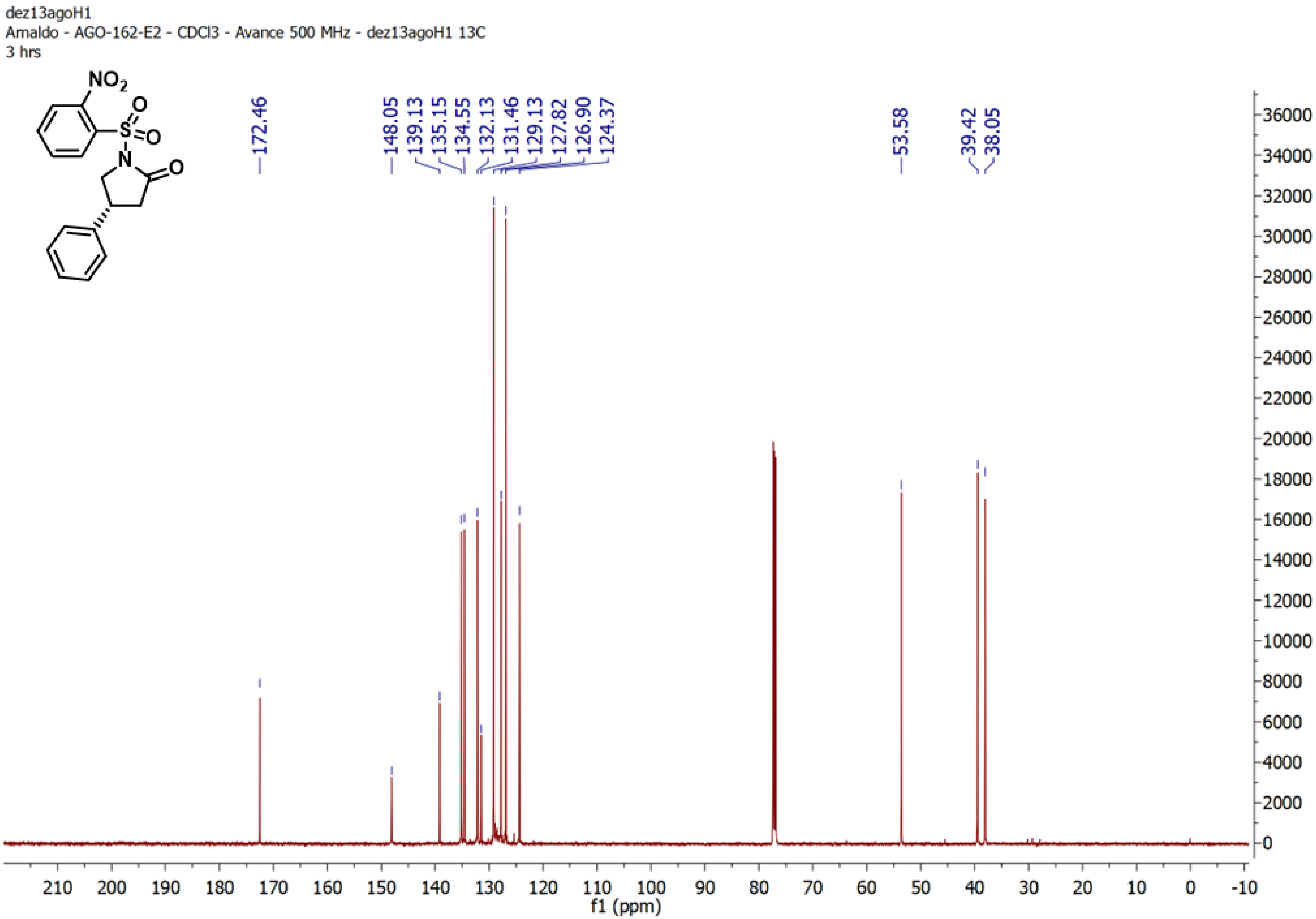

Figure SI53: ^{13}C NMR of compound **4cb**.

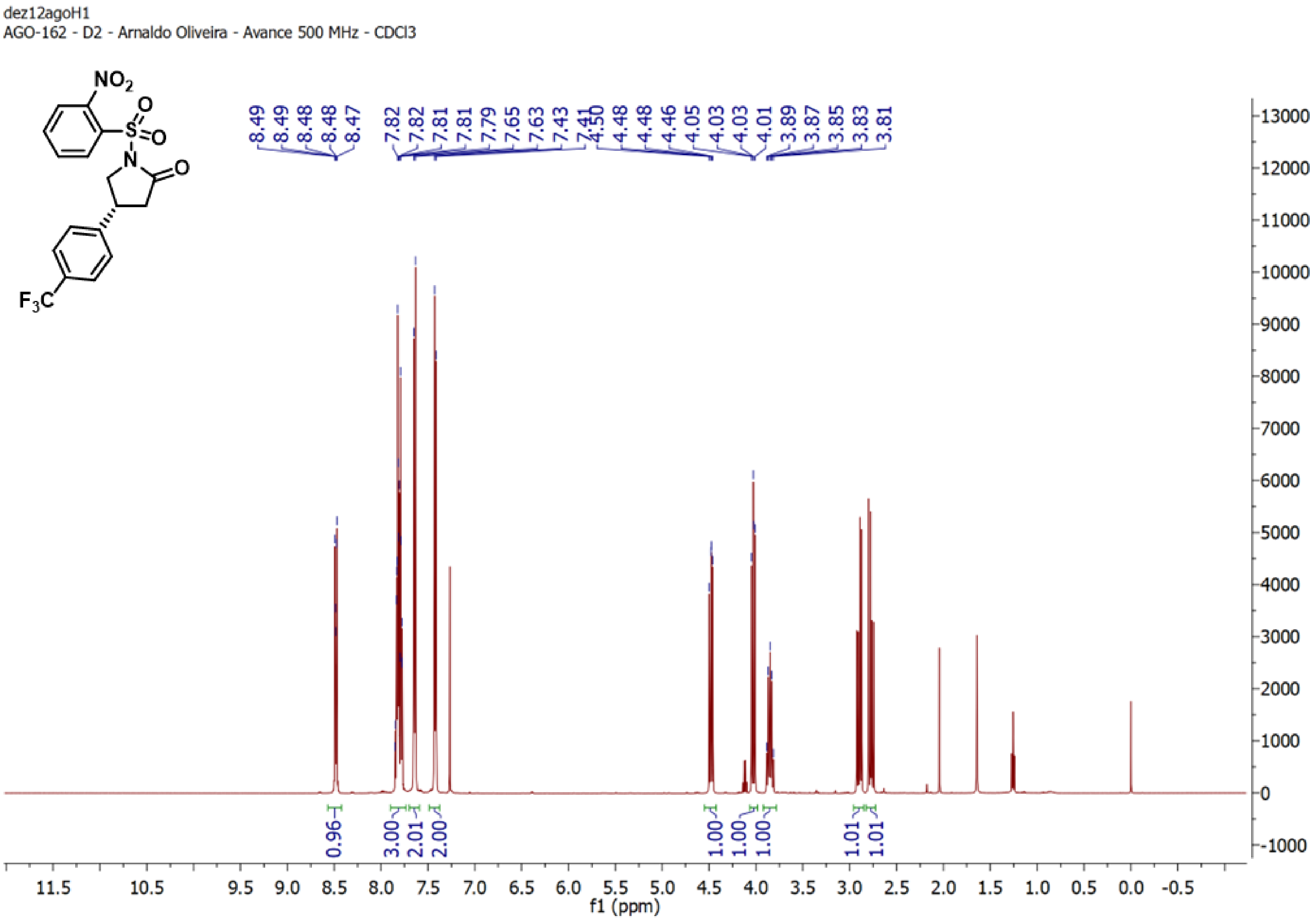

Figure SI54: ^{1}H NMR of compound **4cc**.

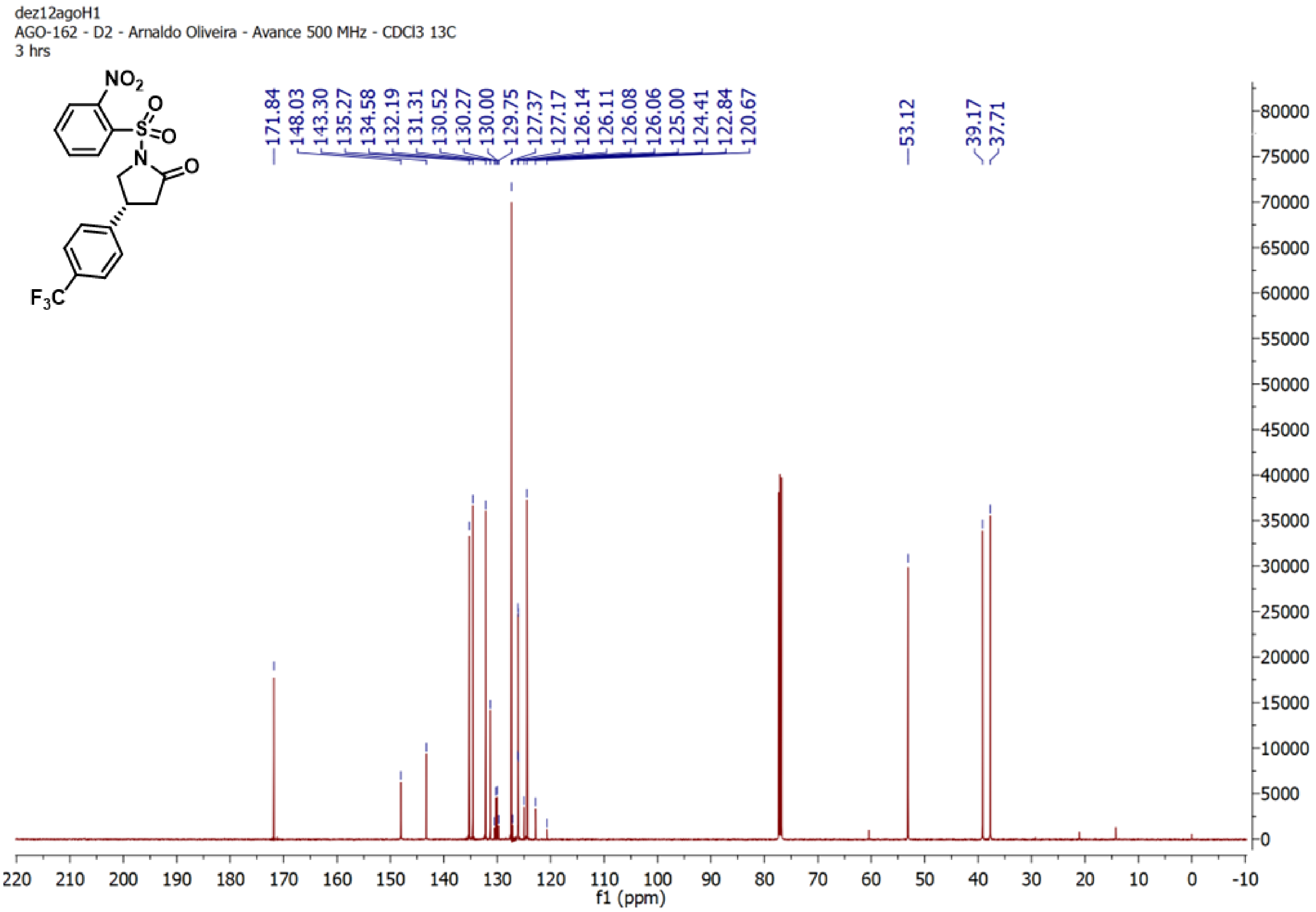

Figure SI55: ^{13}C NMR of compound **4cc**.

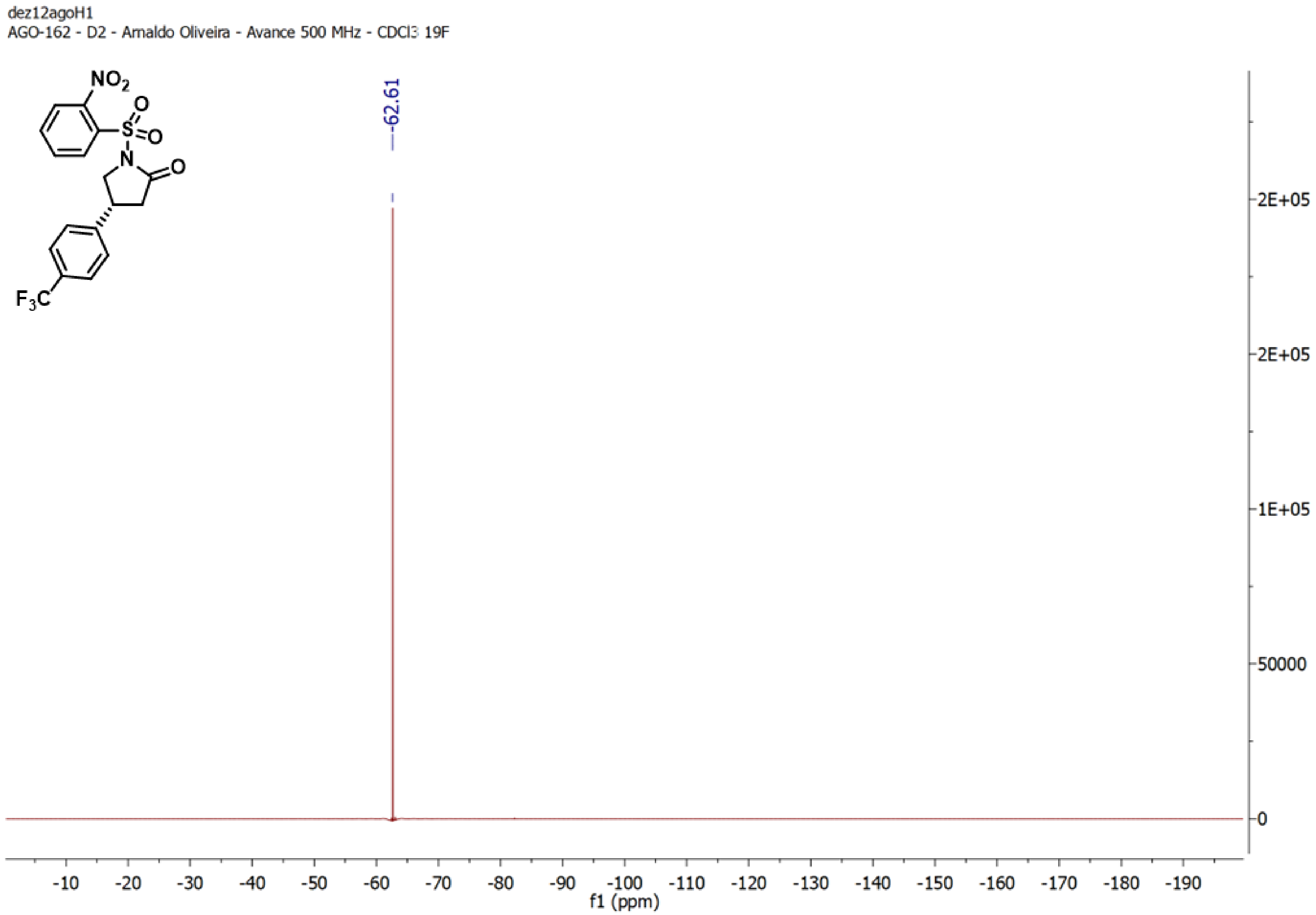

Figure SI56: ^{19}F NMR of compound **4cc**.

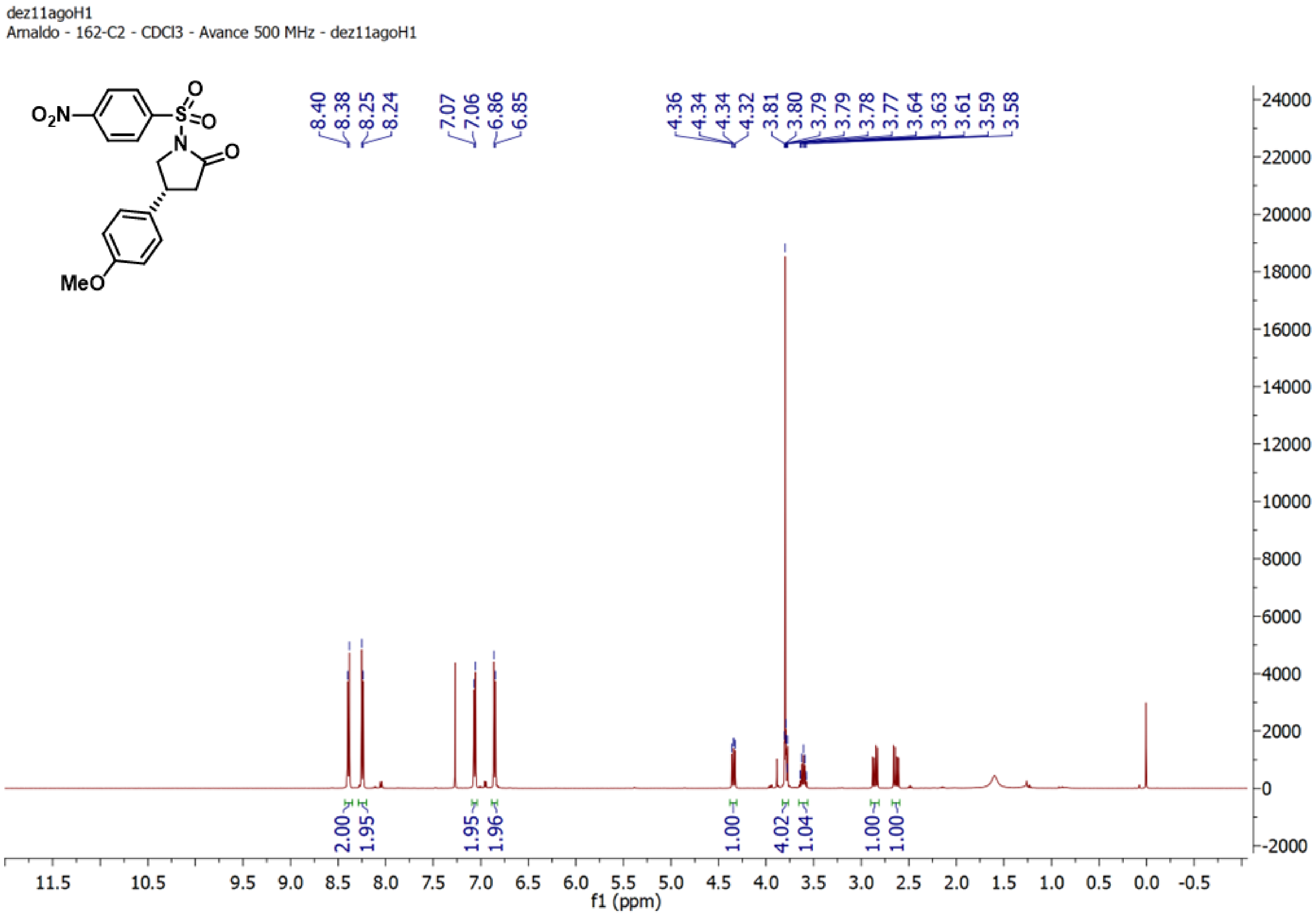

Figure SI57: ^{1}H NMR of compound **4da**.

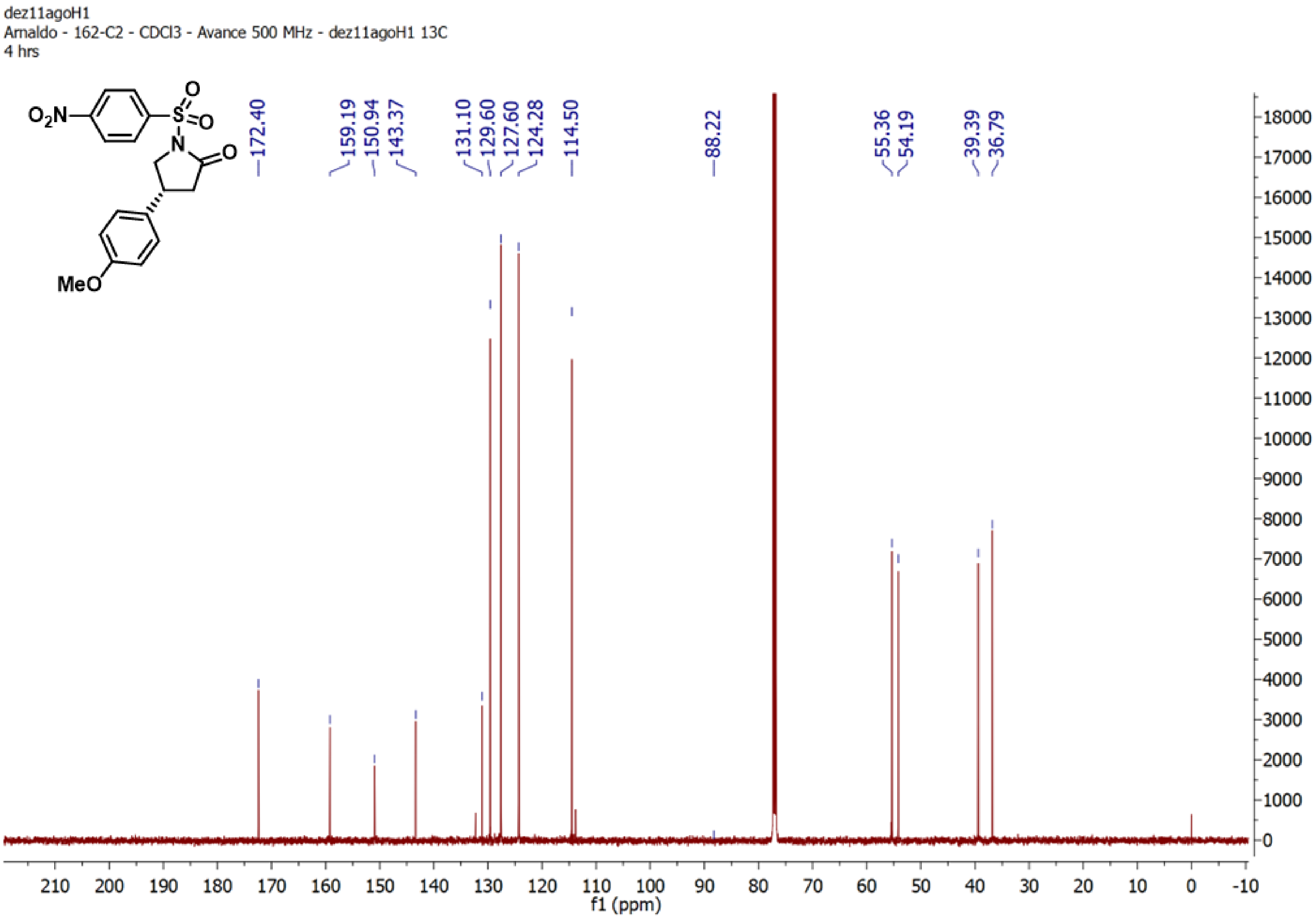

Figure SI58: ^{13}C NMR of compound **4da**.

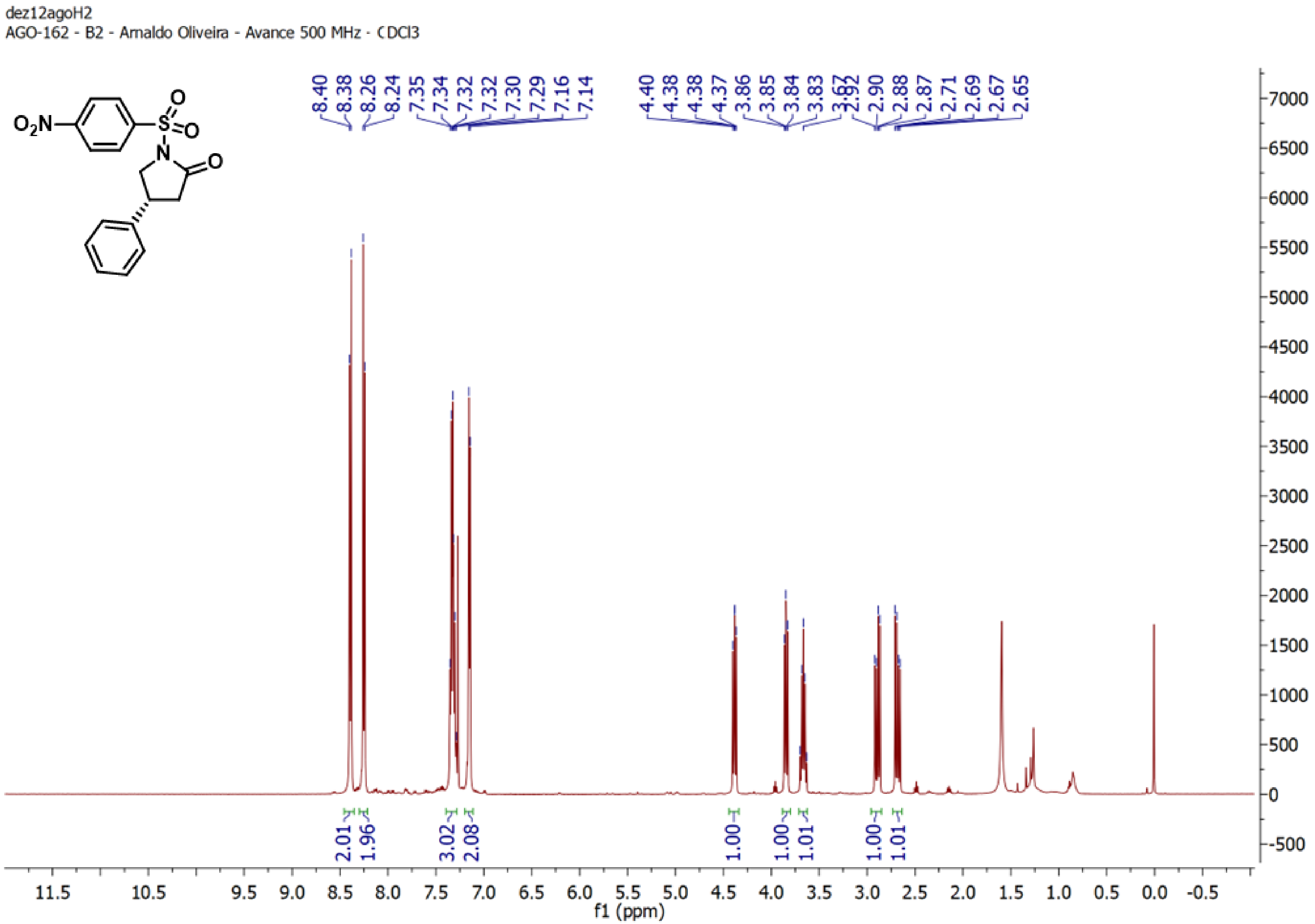

Figure SI59: ^{1}H NMR of compound **4db**.

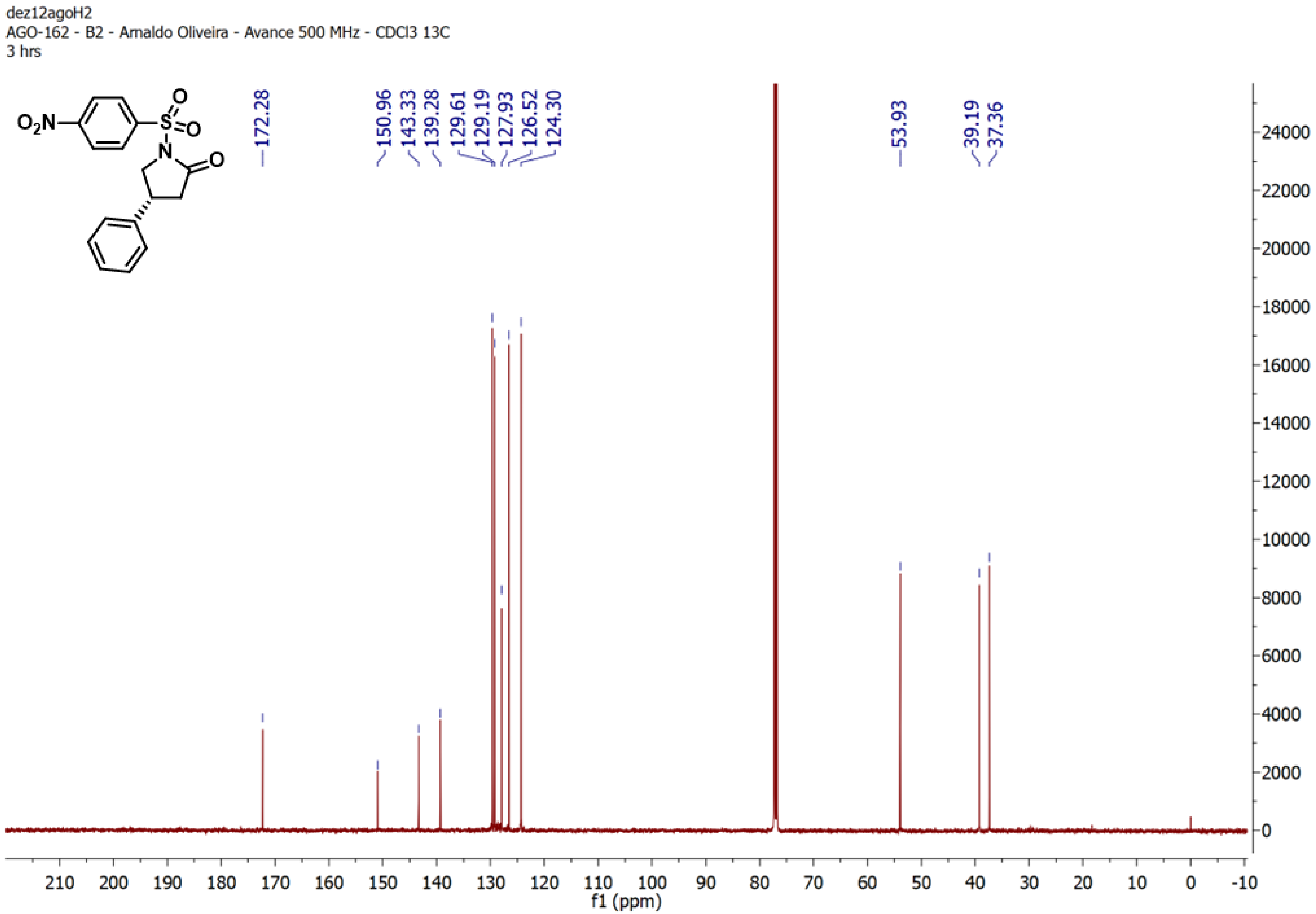

Figure SI60: ^{13}C NMR of compound **4db**.

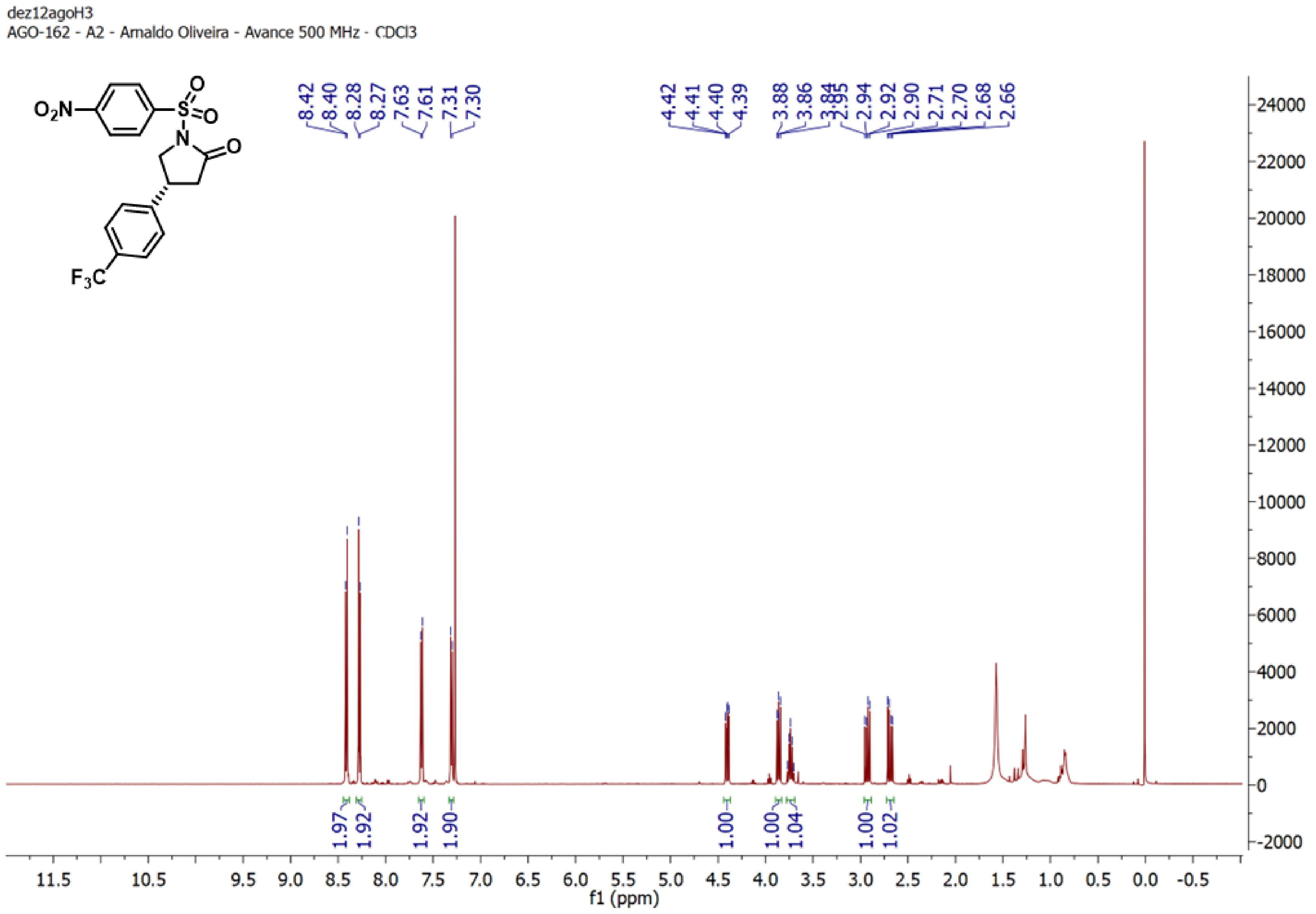

Figure SI61: ^{1}H NMR of compound **4dc**.

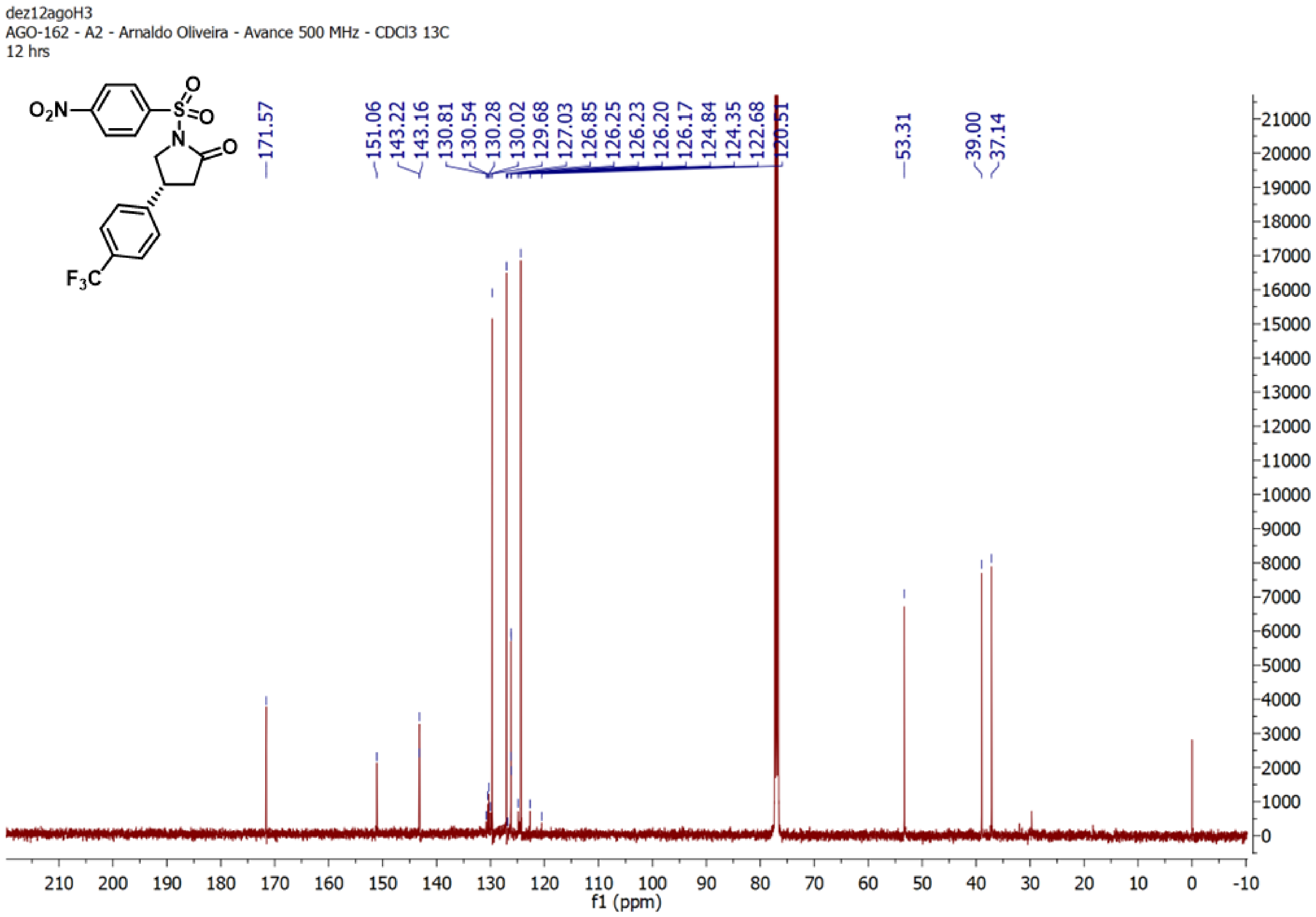

Figure SI62: ^{13}C NMR of compound **4dc**.

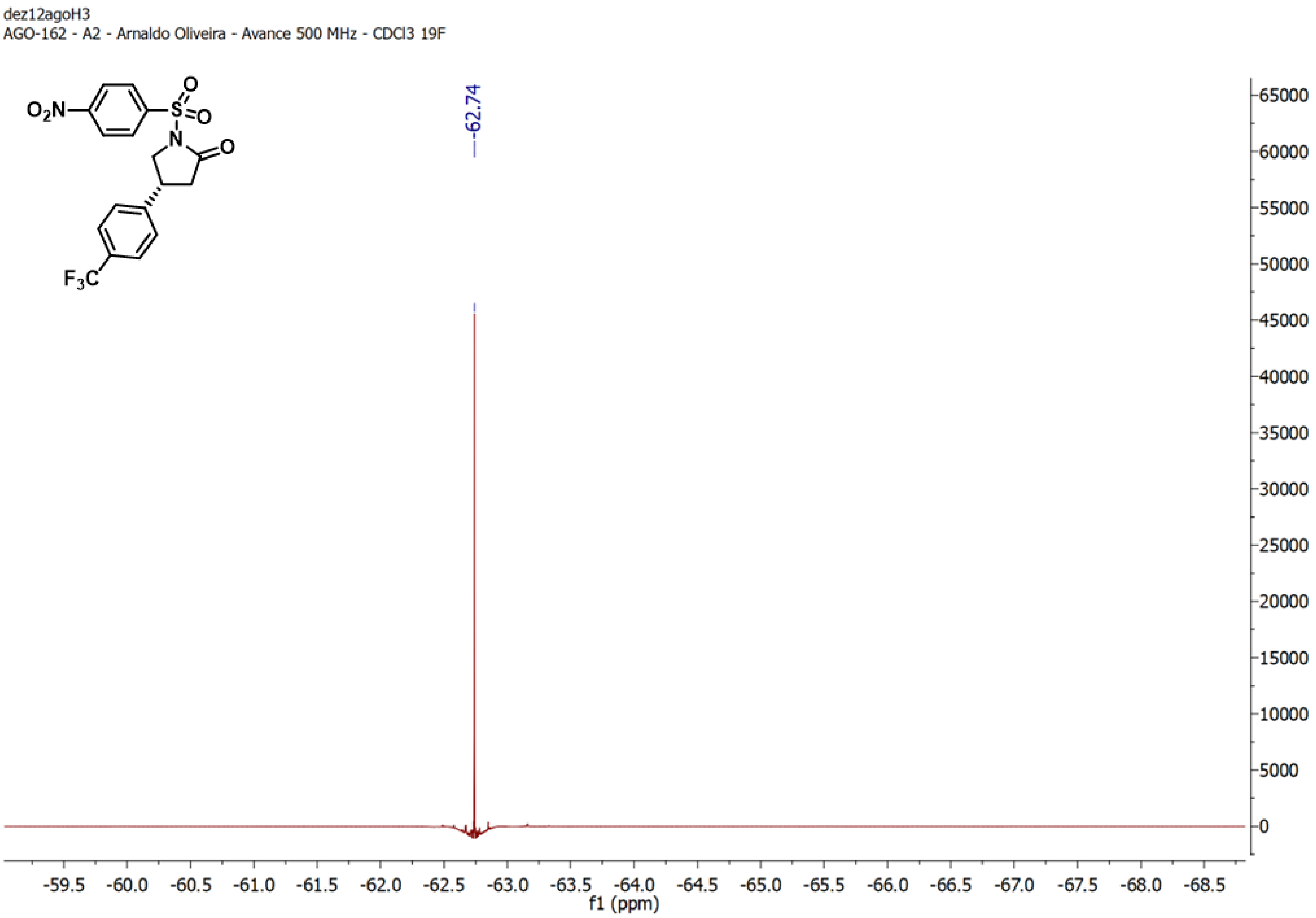

Figure SI63: ^{19}F NMR of compound **4dc**.

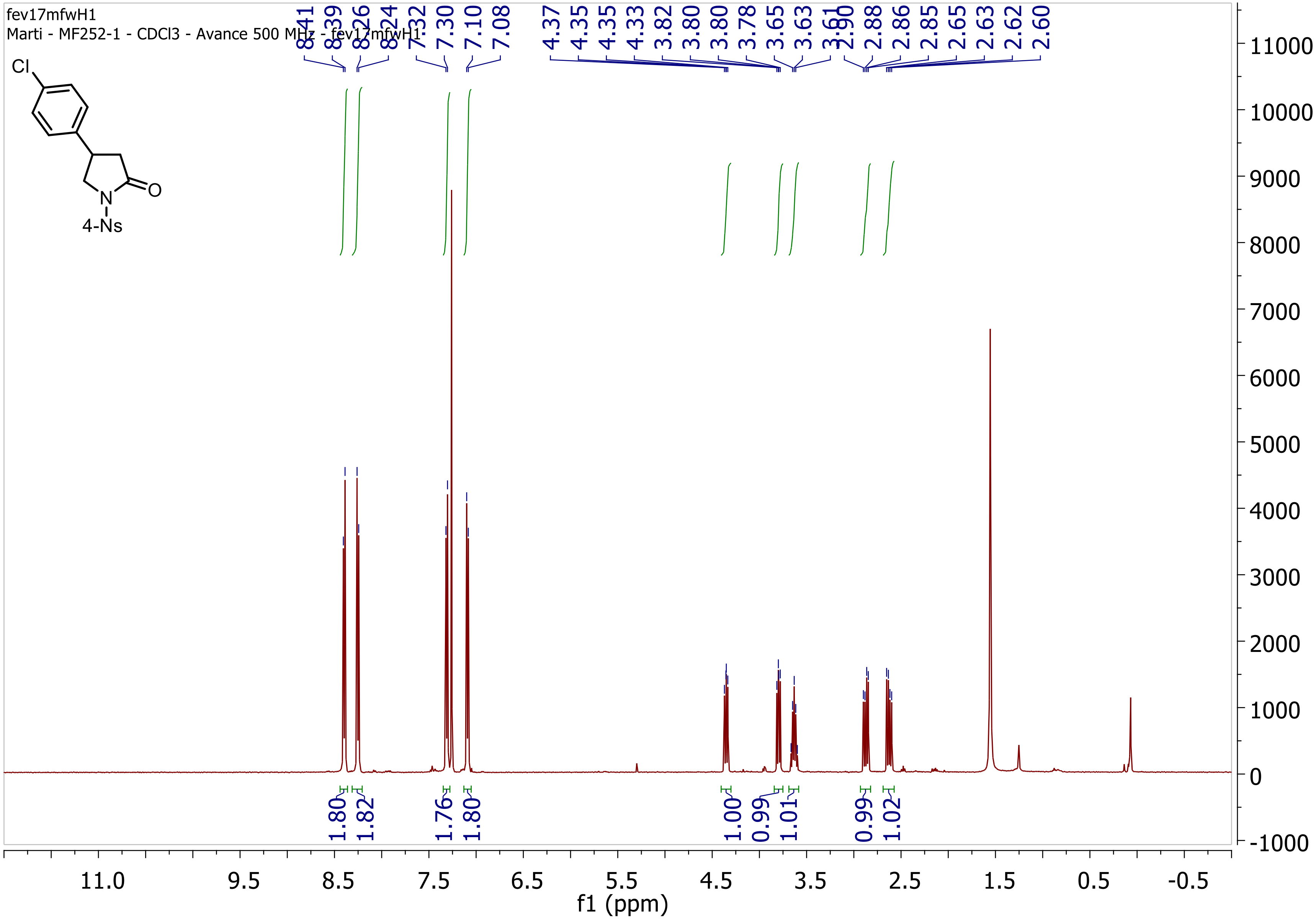

Figure SI64: ^{1}H NMR of compound **4dd**.

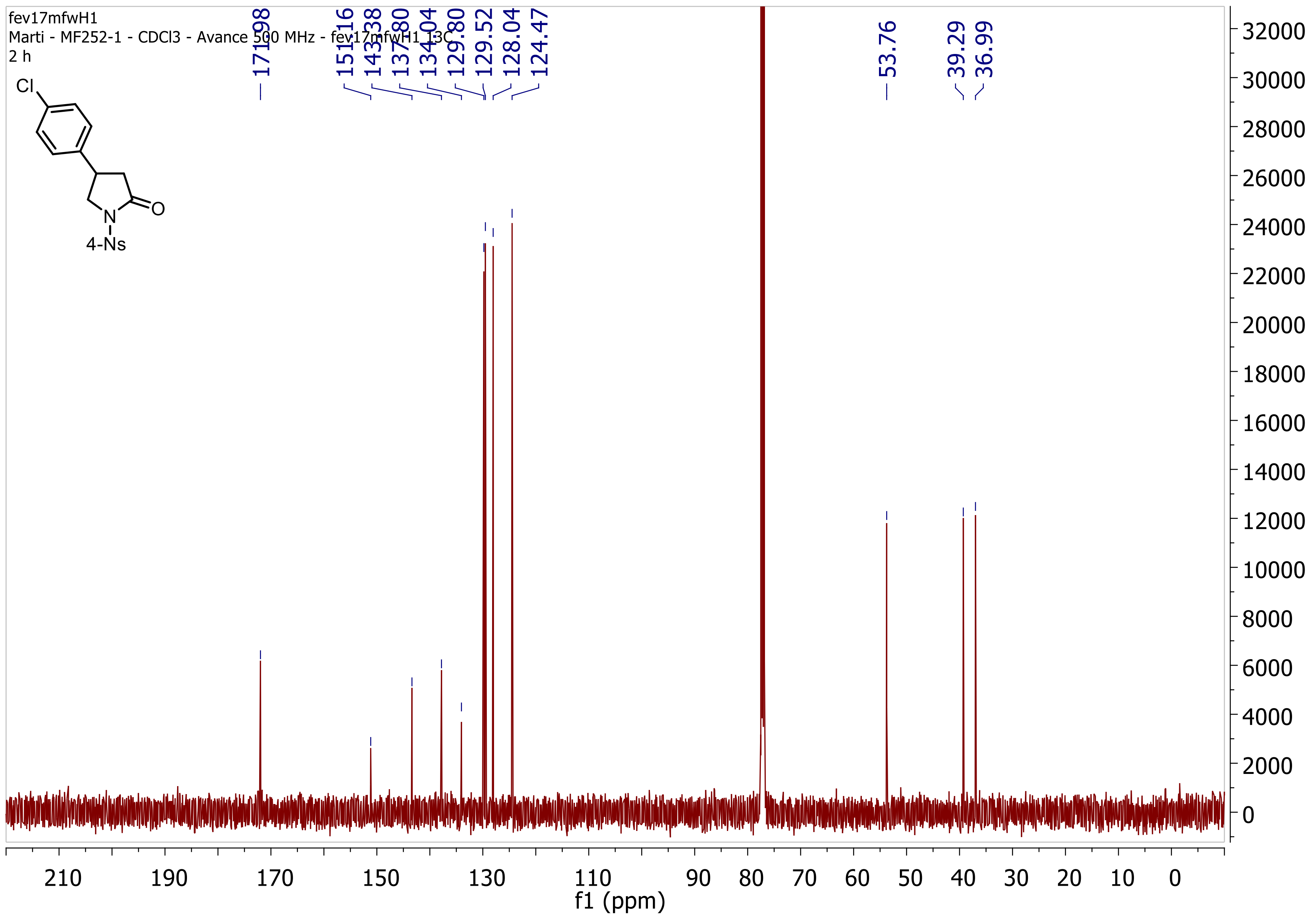

Figure SI65: ^{13}C NMR of compound **4dd**.

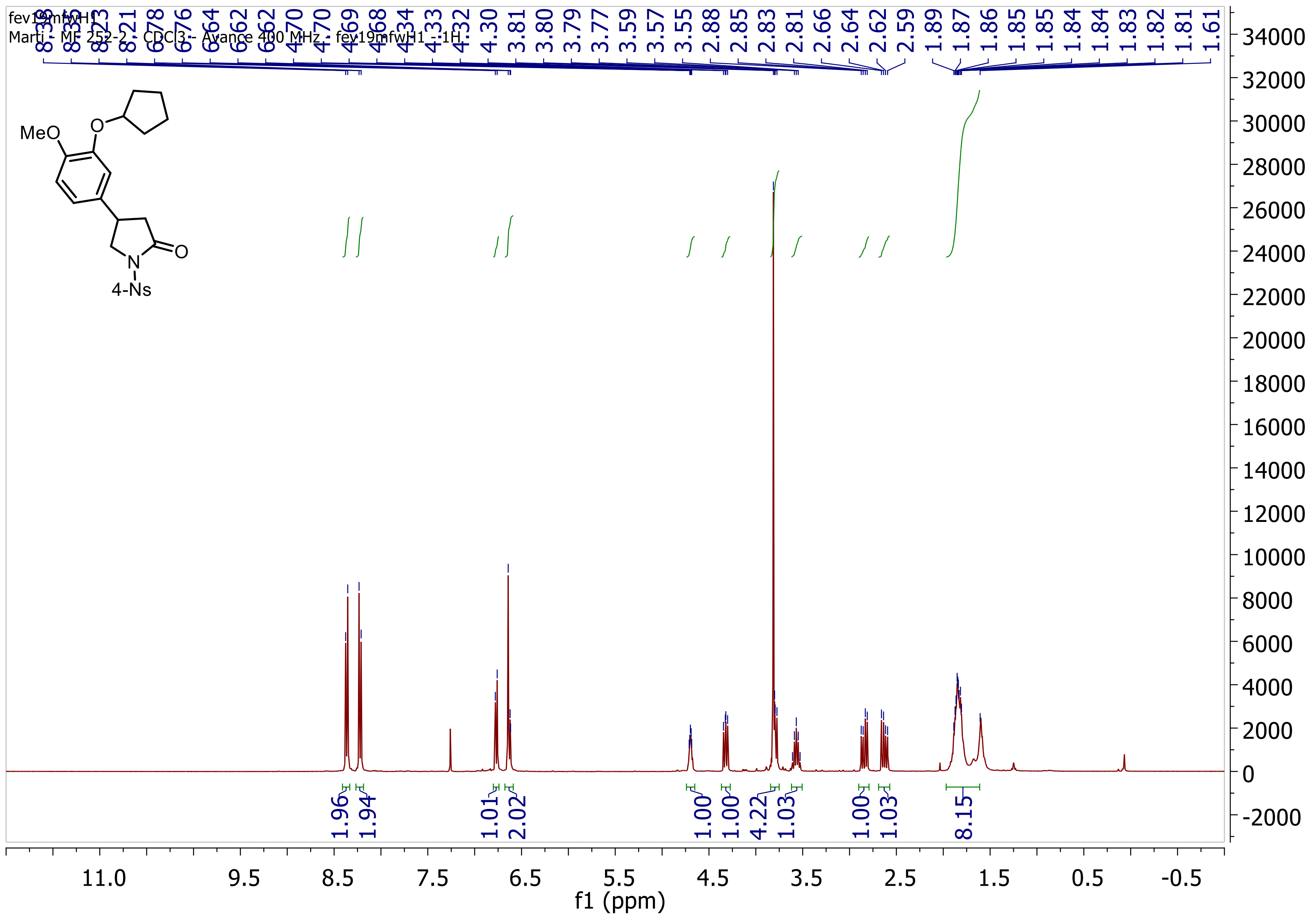

Figure SI66: ^{1}H NMR of compound **4de**.

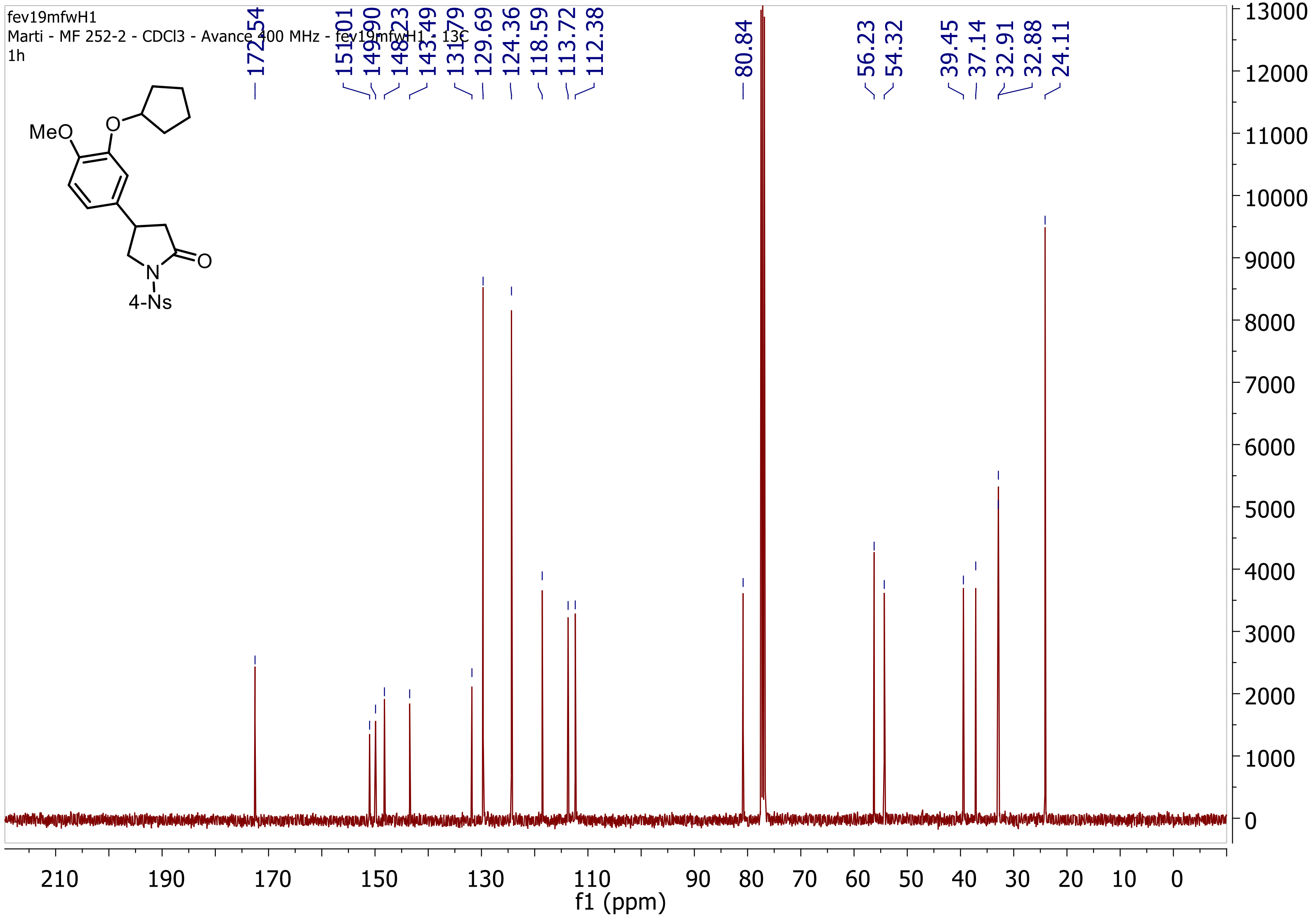

Figure SI67: ^{13}C NMR of compound **4de**.

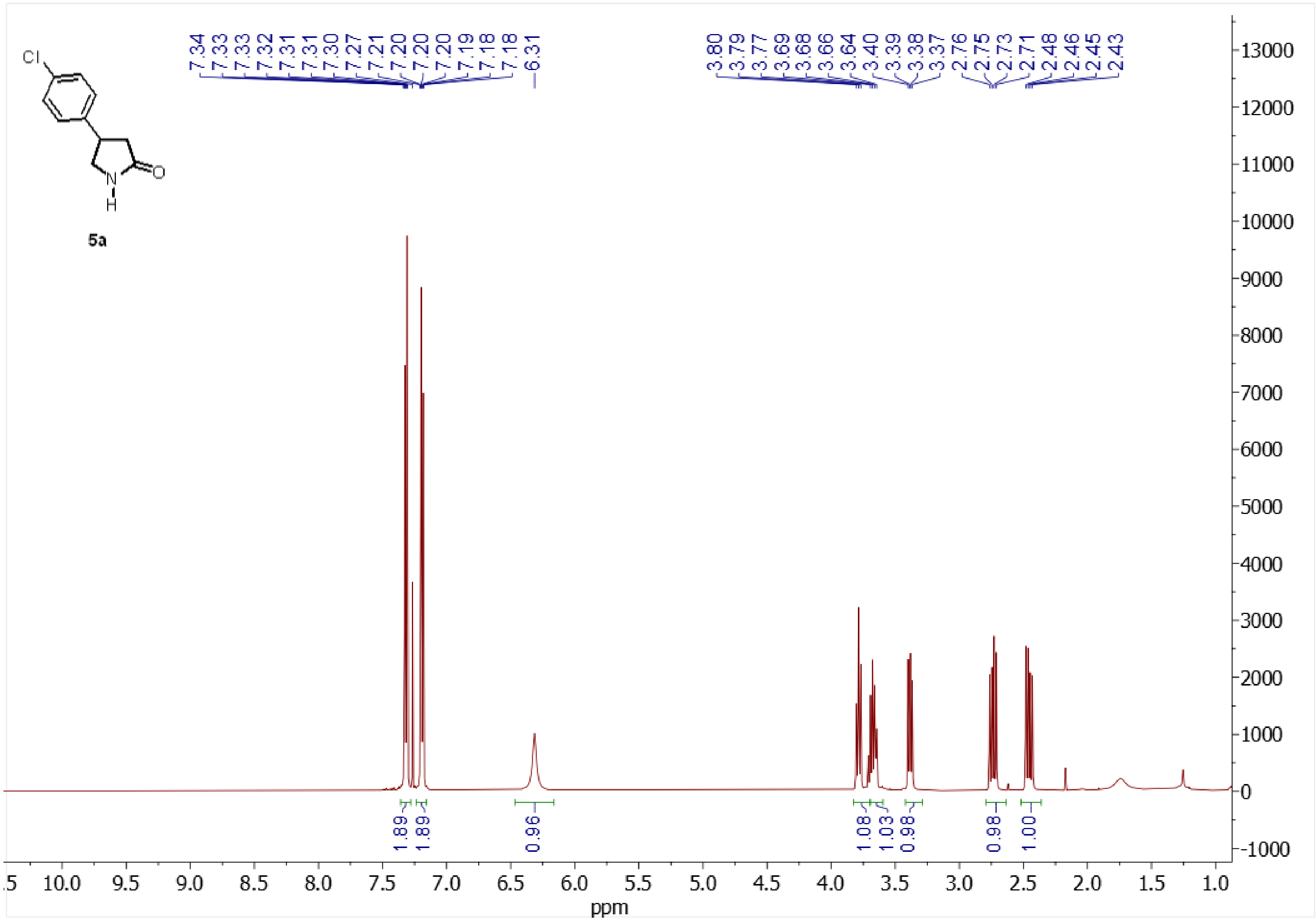

Figure SI68: ^{1}H NMR of compound **5a**.

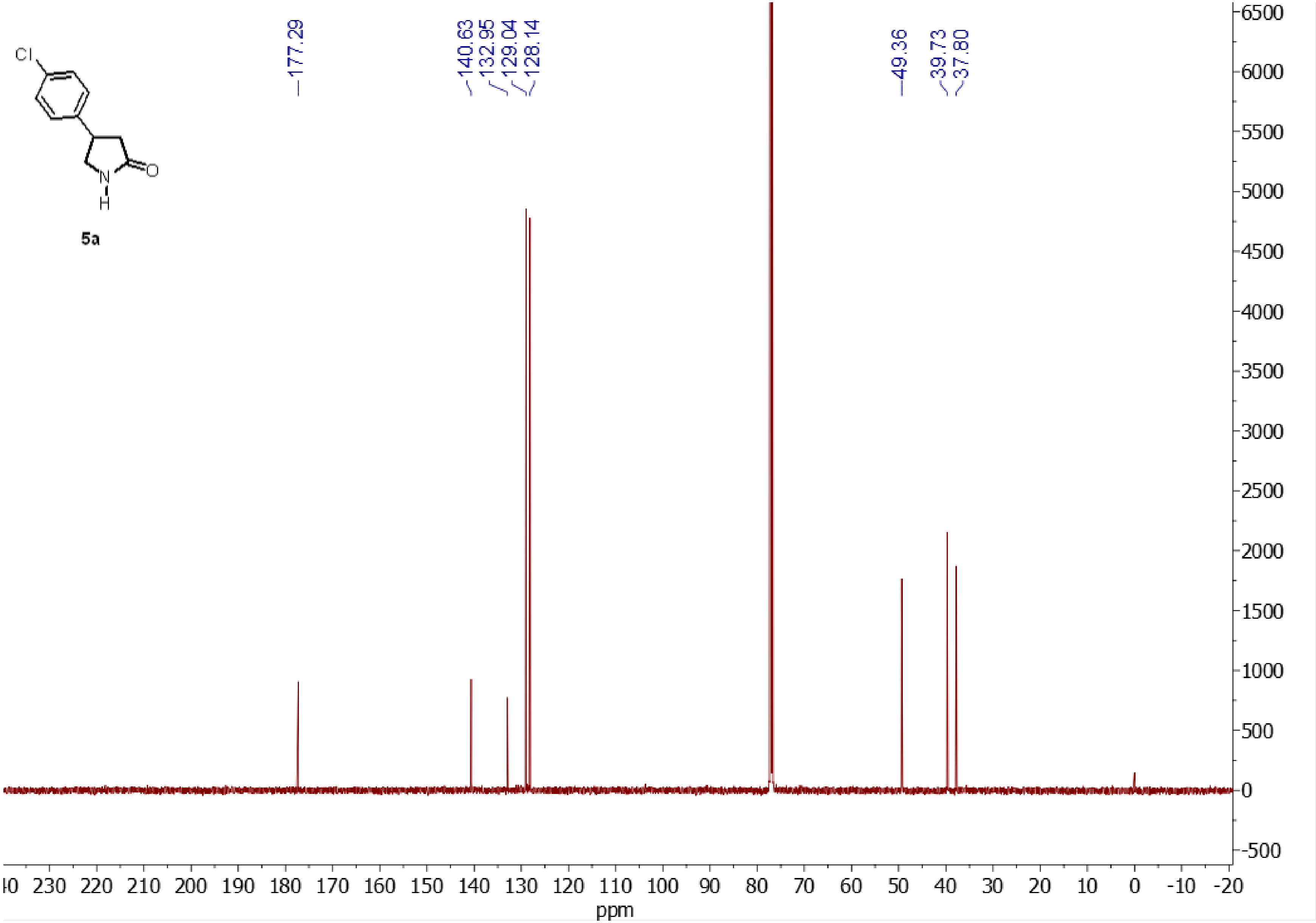

Figure SI69: ^{13}C NMR of compound **5a**.

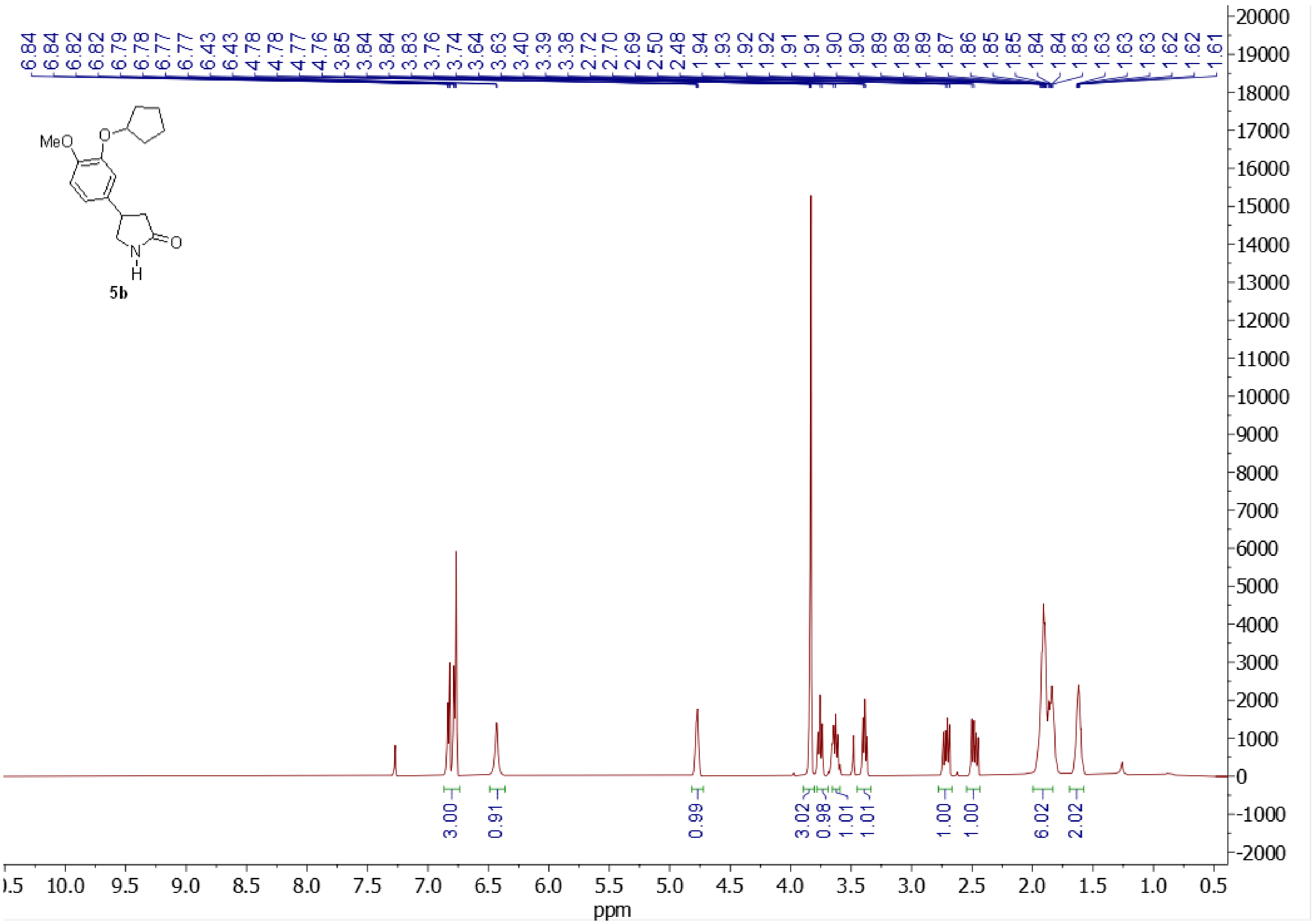

Figure SI70: ^{1}H NMR of compound **5b**.

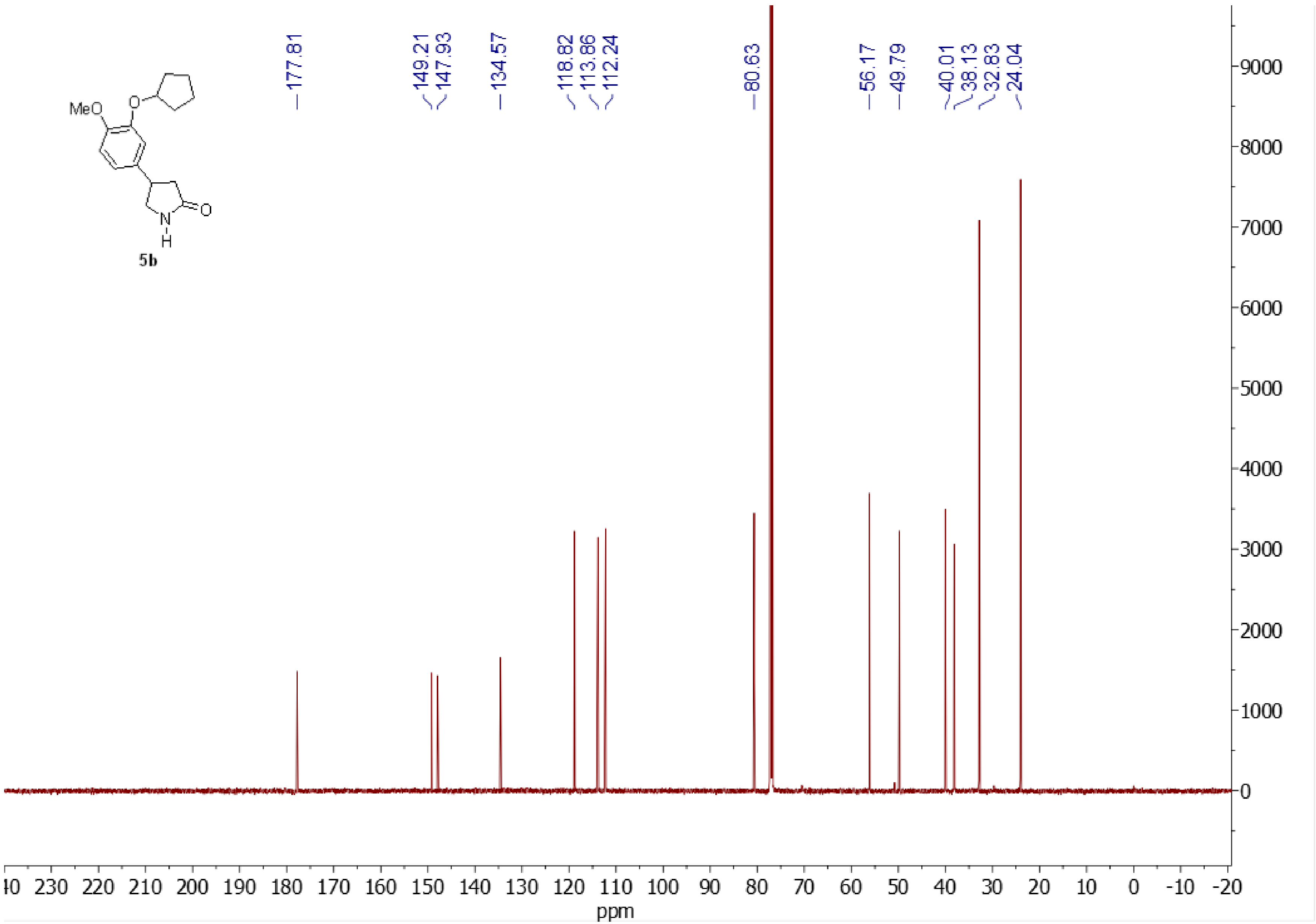

Figure SI71: ^{13}C NMR of compound **5b**.

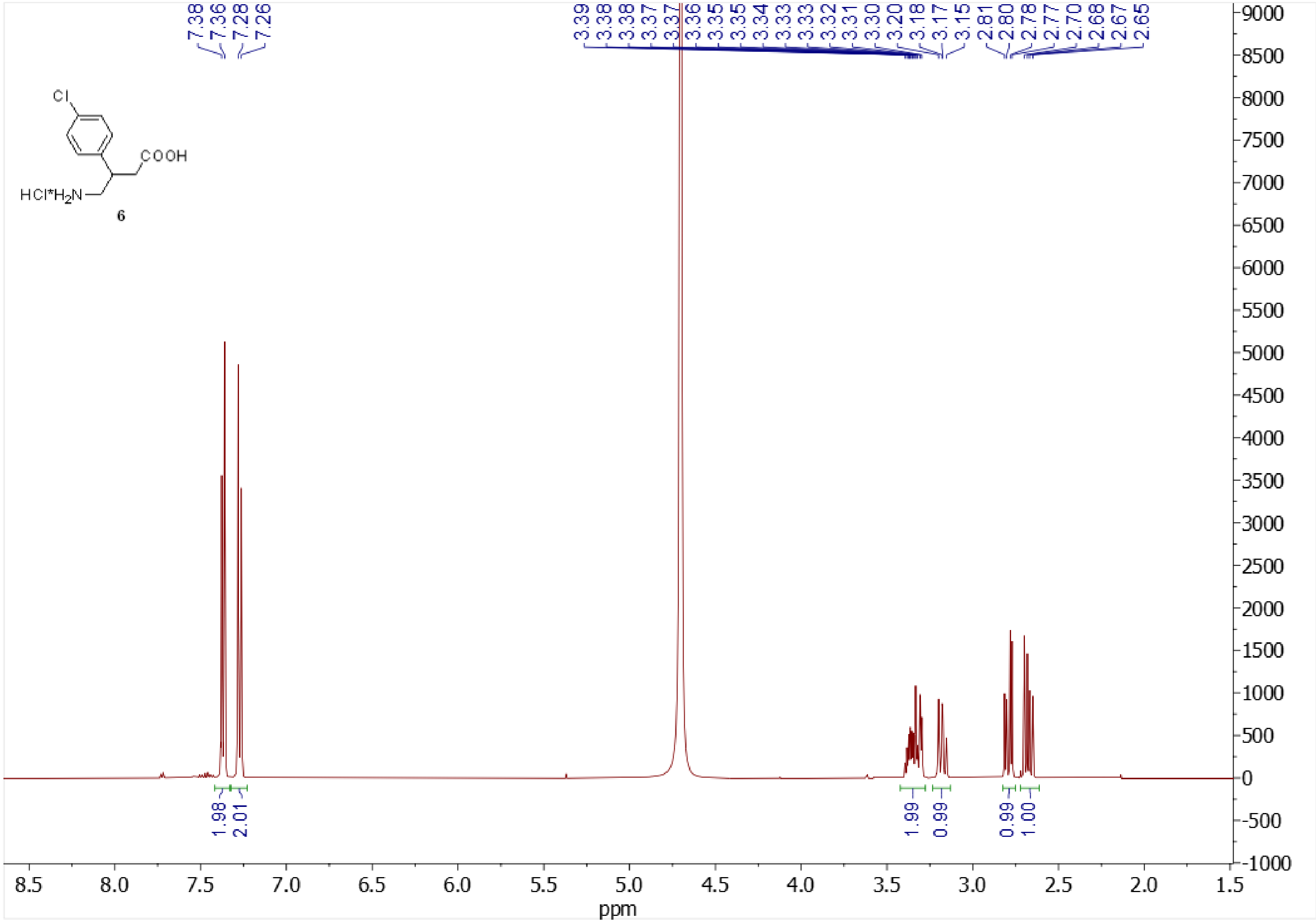

Figure SI72: ^{1}H NMR of compound **6**.

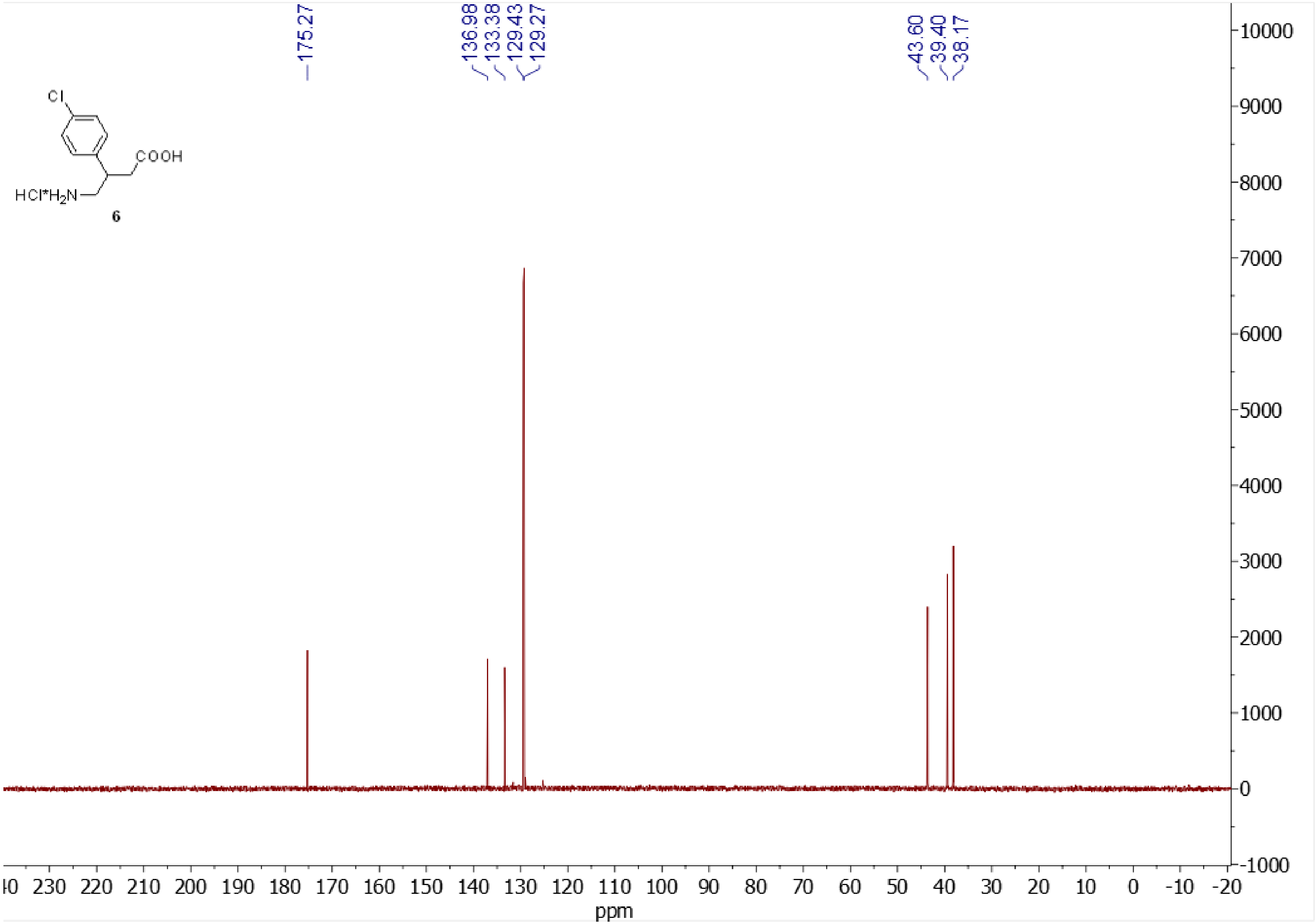

Figure SI73: ^{13}C NMR of compound **6**.

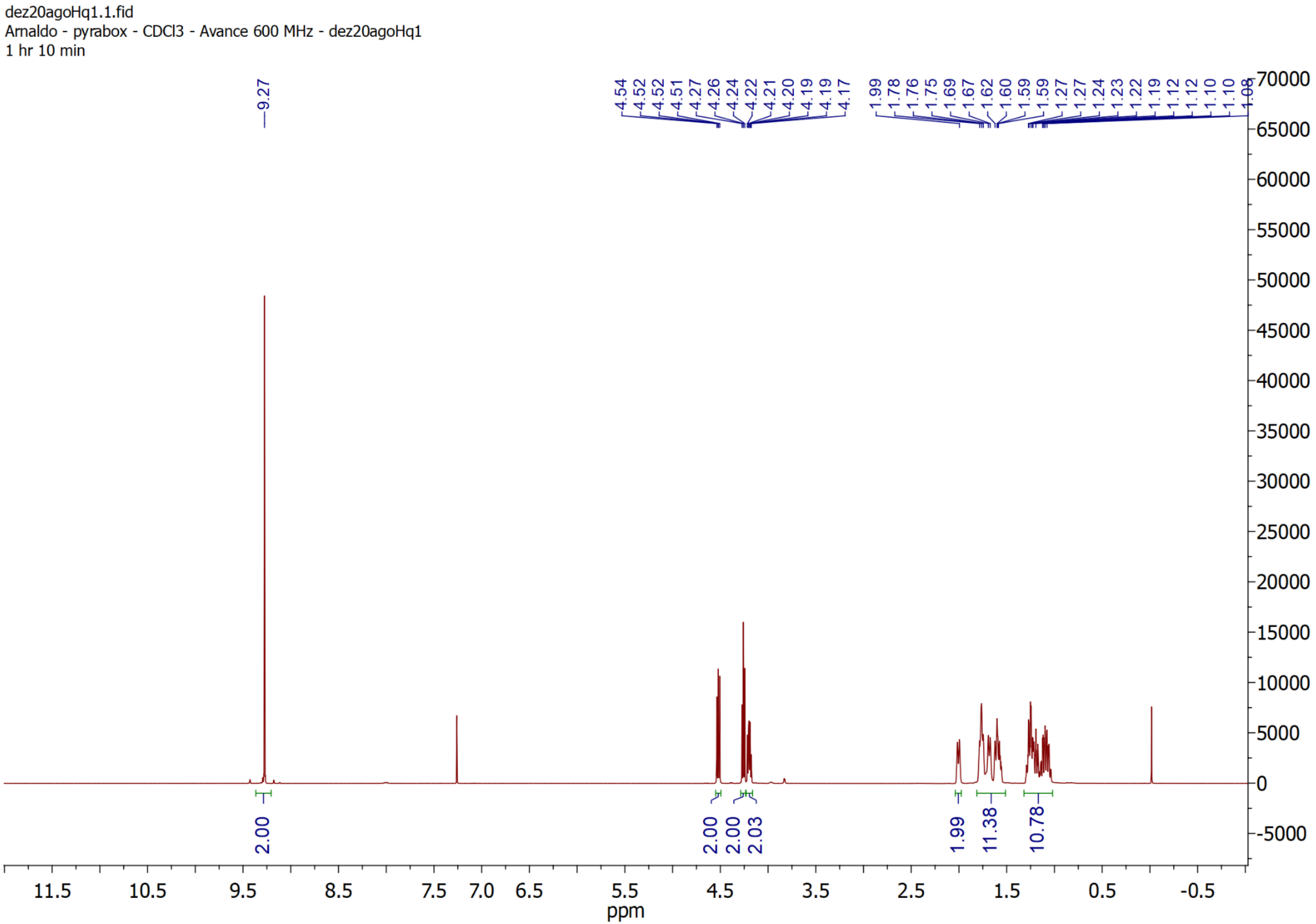

Figure SI74: ^{1}H NMR of ligand **L2**.

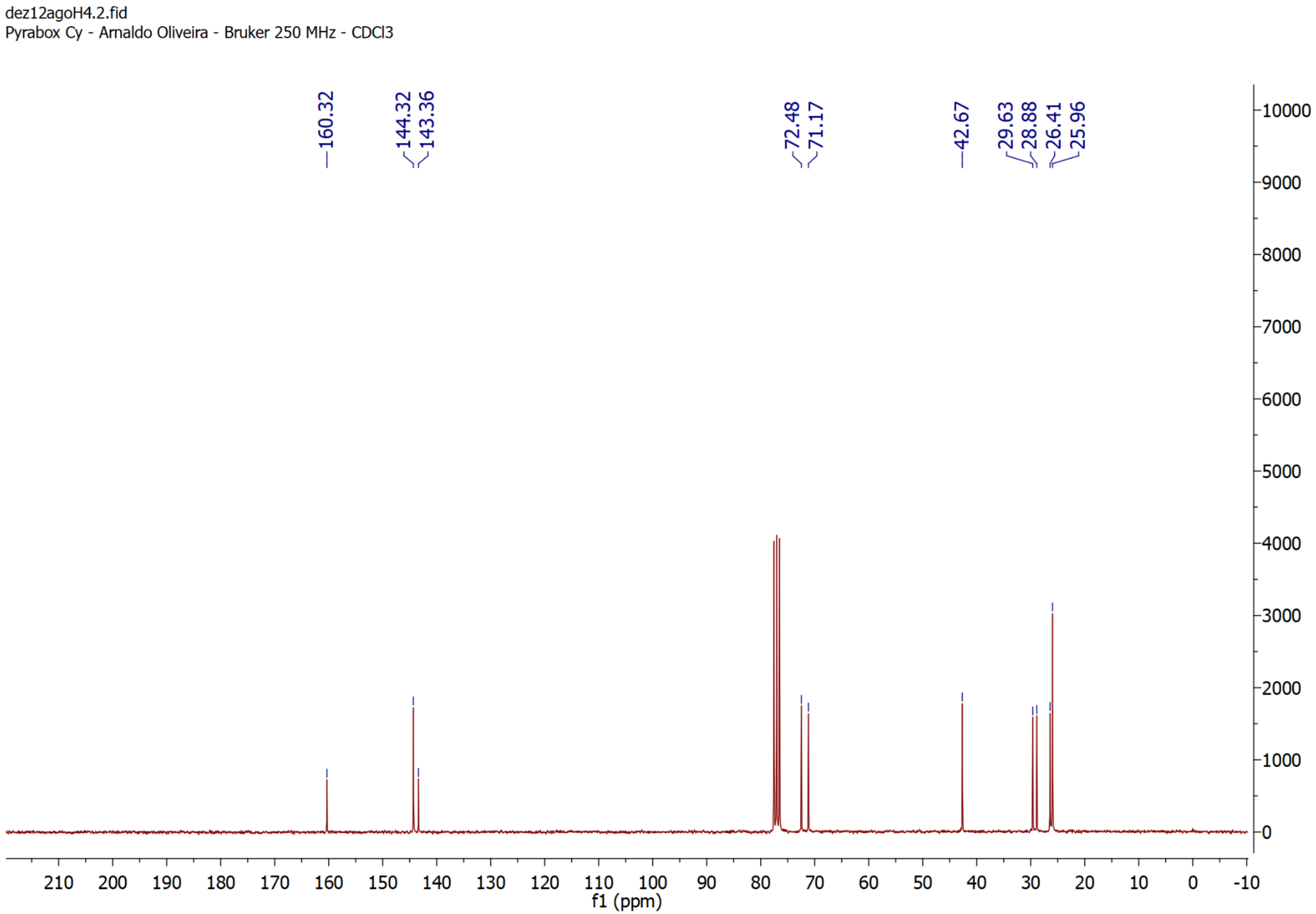

Figure SI75: ^{13}C NMR of ligand **L2**.

7. References

(1) Oliveira, C. C.; Pfaltz, A.; Correia, C. R. D. *Angew. Chem. Int. Ed* **2015**, *54*, 4036–14039.

(2) Li, Y.; Li, Y.; Peng, L.; Wu, D.; Zhu, L.; Yin, G. *Chem. Sci.* **2020**, *11*, 10461–10464.

(3) Werner, E. W.; Mei, T. S.; Burckle, A. J.; Sigman, M. S. *Science (1979)* **2012**, *338*, 1455–1458.

(4) Sandford, C.; Fries, L. R.; Ball, T. E.; Minteer, S. D.; Sigman, M. S. *J Am Chem Soc* **2019**, *141*, 18877–18889.

(5) Cerezo, S.; Cortés, J.; Moreno-Mañas, M.; Pleixats, R.; Roglans, A. *Tetrahedron* **1998**, *54*, 14869–14884.

(6) So, C. M.; Kume, S.; Hayashi, T. *J. Am. Chem. Soc.* **2013**, *135*, 10990–10993.

(7) Longshaw, A. I.; Adanitsch, F.; Gutierrez, J. A.; Evans, G. B.; Tyler, P. C.; Schramm, V. L. *J Med Chem* **2010**, *53*, 6730–6746.

(8) Do, J.-L.; Mottillo, C.; Tan, D.; Strukil, V.; Frišcic, T. C.; *J. Am. Chem. Soc.* **2015**, *137*, 7, 2476.

(9) Shao, C.; Yu, H. J.; Wu, N. Y.; Tian, P.; Wang, R.; Feng, C. G.; Lin, G. Q. *Org Lett* **2011**, *13*, 788–791.
